# Supplementary material for: Decision to self-isolate during the COVID-19 pandemic in the UK: a rapid scoping review
Source: BMJ Open. 2024 Mar 29;14(3):e084437. doi: 10.1136/bmjopen-2024-084437 (PMC10982762; doi:10.1136/bmjopen-2024-084437)
Supplement: Supplementary data [file bmjopen-2024-084437supp001.pdf]

BMJ Publishing Group Limited (BMJ) disclaims all liability and responsibility arising from any reliance placed on this supplemental material which has been supplied by the author(s)

Decision to self-isolate during the COVID-19 pandemic in the UK: a rapid scoping study

Supplemental tables and figures

Supplemental Table 1. Search categories and examples of search terms. Search strings were adapted to each database. 2
Supplemental Table 2. Justifications and rationale for databases searched 3
Supplemental Table 3. Data extraction template 4
Supplemental Table 4. Included sources in the study examining self-isolation behaviour during the COVID-19 pandemic in the UK 5
Supplemental Table 5. Definitions of the stages of the pandemic 34

Supplemental Figure 1. Infographic to define the stages of the pandemic, with details 35

Keene CM, *et al. BMJ Open* 2024; 14:e084437. doi: 10.1136/bmjopen-2024-084437

Supplemental Table 1. Search categories and examples of search terms. Search strings were adapted to each database.

Category	Search terms
**COVID-19**	COVID* OR corona OR coronavirus OR SARS-CoV-2 OR "SARS CoV 2" OR "SARS CoV-2" OR SARS-CoV2 OR SARSCoV2 OR "Severe Acute Respiratory Syndrome Coronavirus 2" OR "Severe Acute Respiratory Syndrome Corona Virus 2" OR 2019-nCoV
	**AND**
**Key activities**	test* OR screen* OR RT-PCR OR PCR OR "polymerase chain reaction" OR "lateral flow" OR "lateral flow device*" OR "lateral flow assay*" OR LFD OR self-test* OR "test and trace" OR "contact trac*" OR surveillance OR POCT OR report* OR self-report* OR selfreport* OR "test positive" OR "testing positive" OR result* OR "self-isolation" OR "self isolation" OR isolat* OR containment OR reopening OR re-opening OR mitigat* OR flatten*
	**AND**
**Behaviour, barriers, and facilitators**	knowledge OR understand* OR attitude* OR perception* OR perceive OR belief* OR believ* OR expectation* OR trust OR willing* OR intention* OR behaviour* OR behavior* OR practice* OR enact* OR engag* OR adher* OR complian* OR comply OR experience* OR view* OR motivation* OR barrier* OR block* OR challeng* OR difficult* OR facilitat* OR enabl* OR access* OR feasib* OR accept* OR uptake
	**AND**
**Research methods**	qualitative* OR interview* OR FGD OR "focus group*" OR survey* OR questionnair* OR mixed-method* OR "mixed method*" OR ethnograph* OR theme OR thematic* OR "grounded theory" OR "content analysis" OR field-work OR "field work" OR selfreport* OR self-report* OR "self report*" OR view* OR experience* OR hermeneutic OR phenomenolog*
	**AND**
**Geographic setting**	"United Kingdom" OR UK OR England OR Ireland OR Irish OR Scot* OR Wales OR Britain OR British OR NHS OR "National Health Service*" OR UKHSA OR "United Kingdom Health Security Agency" OR "UK Health Security Agency" OR "Channel Island*" OR London OR Birmingham OR Liverpool OR Manchester OR Cardiff OR Belfast OR Edinburgh OR Glasgow

Supplemental Table 2. Justifications and rationale for databases searched

Database	Rational for inclusion in the search strategy
**PubMed**	As the primary database for biomedical and health behaviour literature, PubMed not only covers Medline but also offers a vast range of other relevant sources. This includes in-process citations, out-of-scope citations, ahead of print citations, and author manuscripts of NIH funded research, making it a pivotal source for comprehensive and timely information.[1]
**Scopus**	Scopus provides interdisciplinary coverage, encompassing nearly 100 million biomedical, physical, life, and social sciences research records from over 7000 publishers. Its extensive citation analysis capabilities also help to understand the breadth and depth of literature influence. Additionally, the database is curated by independent subject matter experts, recognised as leaders in their fields, adding to the credibility for information for this study.[2]
**The World Health Organization COVID-19 Research Database**	The literature cited in the WHO COVID-19 Research Database was updated daily (Tuesday through Saturday) during the pandemic from searches of bibliographic databases, hand searching, and the addition of other expert-referred scientific articles. This database represents a comprehensive multilingual source of current literature on the topic, with the backing and expertise of a global health authority. While it may not be exhaustive, new research was added regularly. Databases searched include MEDLINE, Scopus, EuropePMC, Web of Science, ProQuest Central, EMBASE, medRxiv, ICTRP, WHO COVID, ScienceDirect, and grey literature.[3]
**Google Scholar**	Google Scholar covers a broad range of literature types, including theses, conference proceedings, and articles from academic publishers. Its inclusion can identify research overlooked in more specialized databases, ensuring comprehensive coverage and filling obvious gaps.
**Review of bibliographies of included studies**	A classic snowballing method, reviewing bibliographies can unveil seminal and influential works that may not be captured by traditional database searches. This method ensures inclusion of studies deemed foundational or highly relevant by scholars in the field and identifies evidence deemed relevant by those sources included in the initial screening process.
**Stakeholder consultation**	A strength of scoping studies is the explicit involvement of stakeholders in not only framing the research question, sense-checking the results, and informing the interpretation but also in identifying critical sources of evidence that may otherwise have been missed.[4]<br>In this instance, the UK Health Security Agency (UKHSA) was consulted as a major stakeholder to identify research commissioned by UKHSA.

Supplemental Table 3. Data extraction template

Manuscript information																								
Publication											Methodology						Setting				Key messages	Notes	Bibliographic review	Operational insights
No.	Rayyan ID	Authors	Title	**Inclusion**	**Reason for exclusion**	**EXTRACTOR *[Name]***	Publication date *[year]*	Journal or Source	**Publication stage**	Notes	**Methods**	Participants *[Who were the interviews/ surveys with? Whose views and experiences are represented?]*	Number of participants	**Data collection period *NB!***	Obvious limitations *[copy and paste limitations from the limitations section]*	Notes *[e.g. if 'other' chosen, expand here]*	**Country**	**Scope**	**Site/ Setting**	Notes	***Summary of the findings*** *- bullet points - big picture overview of the key points for OUR question (not the aims of the paper) - add own summary of the key points (paraphrase and summarise) and can copy and paste the conclusions if they summarise the key findings we need*	*Anything that doesn't fit in the categories, explanations etc <As well as your thoughts and notes and interpretations>*		
1																								
2																								
3																								
4																								
5																								

	Isolation Behaviour - copy and paste results and quotes																					
	Isolation								Person												Other	
	Perceived attributes of the isolation itself						Trust		Knowledge				Individual and context									
	**Perceived PHYSICAL factors of isolation itself** *[e.g. physical burden of the isolation itself like (physical) isolation fatigue, discomforts of staying in isolation]*		**Perceived MENTAL factors of isolation itself** *[e.g. like anxiety around isolation, isolation fatigue, "dread" of isolation (again)]*		**Perceived RESOURCE factors of isolation itself** *[e.g. resource factors like financial costs to the user of the isolation process, time etc]*		**Trust in the process of isolation and system** *[e.g. credibility and trust in the institutions delivering/providing the isolation or guidance on isolation)*		**Understanding of isolation requirements** *[i.e. does the individual understand if and when they SHOULD isolate? e.g. when, which type of isolation, where to isolate and for how long, including for what symptom]*		**Perceived capability to isolate** *[i.e. does the individual understand HOW to isolate? e.g. understanding of how to do it (in theory) and feeling able to take the steps to isolate]*		**Logistics of isolation** *[i.e. ACCESS to isolation e.g. convenience, ease of access, ease of isolating, resources, ability to isolate within their home and separate from other family members (separate rooms, space etc)]*		**Personal factors influencing isolation** *[e.g. mental health, burnout, resilience, perceptions of risk of contracting COVID, perceptions of risk of consequences if contract COVID etc]*		**Perception of value in isolation** *[e.g. to oneself, to others, to society, as a duty etc, perceived responsibility towards others to isolate, perceptions of COVID threat level, perceptions of necessity to isolate]*		**Perceived consequences of isolation** *[e.g. e.g. not being to work, or having sick leave or having to look after others, impact on finances, education, work, career, caring responsibilities etc]*		**Social influences related to isolation** *[e.g. stigma in taking part/not taking part, perceived freedom to say no/yes to isolation)*		**Other** *[not captured by other categories]*	
	Barriers	Enablers	Barriers	Enablers	Barriers	Enablers	Barriers	Enablers	Barriers	Enablers	Barriers	Enablers	Barriers	Enablers	Barriers	Enablers	Barriers	Enablers	Barriers	Enablers	Barriers	Enablers	Barriers	Enablers
1																						
2																						
3																						
4																						
5																						

Supplemental Table 4. Included sources in the study examining self-isolation behaviour during the COVID-19 pandemic in the UK

No	Publication	Methodology			Setting			
	Reference	Methods	Description of the sample	Data collection period	Pandemic stage (early, mid, late)	Country	Scope	Context
**General**								
**Literature search**								
1	Al-Oraibi A, Fothergill L, Yildirim M, Knight H, Carlisle S, O'Connor M, Briggs L, Morling JR, Corner J, Ball JK, Denning C, Vedhara K, Blake H. Exploring the Psychological Impacts of COVID-19 Social Restrictions on International University Students: A Qualitative Study. Int J Environ Res Public Health. 2022;19(13).	Interview and focus group	A total of eight online focus groups were conducted, comprising 29 international students, with group sizes varying between three to five students. Seventeen one-to-one interviews were held with university staff members.<br>International students - focus groups<br>University staff - interviews	January - February 2021	Mid pandemic	International (including UK)	Isolation	Testing in universities - Focus groups held with international university students, and interviews held with university staff.
2	Blake H, Carlisle S, Fothergill L, Hassard J, Favier A, Corner J, Ball JK, Denning C. Mixed-methods process evaluation of a residence-based SARS-CoV-2 testing participation pilot on a UK university campus during the COVID-19 pandemic. BMC Public Health. 2022;22(1):1470.	Survey, interview, and focus group	University students living onsite<br>Student survey - 152<br>Staff interviews - 13<br>Student focus groups - 30	April - June 2021	Mid pandemic	England	Testing and isolation	Convergent parallel mixed methods evaluating intervention fidelity and barriers/enablers to implementation of an asymptomatic testing programme in university.
3	Blake H, Knight H, Jia R, Corner J, Morling JR, Denning C, Ball JK, Bolton K, Figueredo G, Morris DE, Tighe P, Villalon AM, Ayling K, Vedhara K. Students' Views towards Sars-Cov-2 Mass Asymptomatic Testing, Social Distancing and Self-Isolation in a University Setting during the COVID-19 Pandemic: A Qualitative Study. Int J Environ Res Public Health. 2021;18(8).	Focus groups	25 university students from 1 institution	October 2020	Mid pandemic	England	Testing and isolation	Focus group discussion with university students on testing and isolation during the pandemic. Thematic analysis conducted on output of focus groups.

4	Burns J, Mc Goldrick N, Sigerson D, Edwards M, Culshaw S, Clark C, Watling C, Braid R, O'Keefe E, Gorman M, Conway DI. A Health Inequalities Impact Assessment of the surveillance of COVID-19 in asymptomatic patients attending dental settings in Scotland. Community Dent Health. 2022;39(4):254-9.	Survey	35 dental team members from across Scotland participated in the consultation.	October 2020	Mid pandemic	Scotland	Testing and isolation	A Health Inequalities Impact Assessment (HIIA) was conducted to understand the differential impacts the programme would have on the population and to improve the accessibility of the programme
5	Denford S, Martin AF, Love N, Ready D, Oliver I, Amlot R, Yardley L, Rubin GJ. Engagement With Daily Testing Instead of Self-Isolating in Contacts of Confirmed Cases of SARS-CoV-2: A Qualitative Analysis. Front Public Health. 2021;9:714041. Epub 20210803. doi: 10.3389/fpubh.2021.714041.	Interviews	52 participants, with 35 who had taken part in a feasibility study to evaluate daily contact testing, and 17 who had declined the offer and opted to self-isolate	11 - 23 December 2020 and 4 - 12 January 2021	Mid pandemic	UK	Testing and isolation	Daily contact testing rather than asymptomatic testing – focus on attitudes towards contact testing as an alternative to contacts of positive cases having to isolate. Study ran before Universal Testing roll out.
6	Denford S, Martin AF, Towler L, Mowbray F, Essery R, Bloomer R, Ready D, Love N, Amlot R, Oliver I, Rubin GJ, Yardley L. A qualitative process analysis of daily contact testing as an alternative to self-isolation following close contact with a confirmed carrier of SARS-CoV-2. BMC Public Health. 2022;22(1):1373.	Interviews	60 - People who had been in close contact with a confirmed SARS-CoV-2 carrier and had consented to take part in the trial.	24 June - 8 July 2021	Mid pandemic	England	Testing & Isolation	Daily contact testing rather than asymptomatic testing – focus on attitudes towards contact testing as an alternative to contacts of positive cases having to isolate. Study covers before and after introduction of Universal Testing.
7	Dennis A, Robin C, Carter H. The social media response to twice-weekly mass asymptomatic testing in England. BMC Public Health.	Other – review of	5783 comments – members of public	5 April - 28 May 2021	Mid pandemic	England	Testing & Isolation	Analysis of social media comments from members of

	2022;22(1):182. Epub 20220127. doi: 10.1186/s12889-022-12605-2.	social media						the public in England about twice-weekly asymptomatic testing. Study is from period when Universal Testing was first introduced.
8	Eraso Y, Hills S. Self-Isolation and Quarantine during the UK's First Wave of COVID-19. A Mixed-Methods Study of Non-Adherence. Int J Environ Res Public Health. 2021;18(13). Epub 20210630. doi: 10.3390/ijerph18137015.	Survey, interview, and focus group	residents in six North London boroughs<br>681 - Survey<br>16 – interviews	1 - 31 May 2020	Mid pandemic	England	Isolation	Study conducted before Universal Testing was available
9	Hanley S, Raybould G, Baxter E, Gray J, Sharkey D, Walker KF. Maternity services' responses to the COVID-19 pandemic: how Public Health England guidance was implemented in practice. Journal of Hospital Infection. 2022;129:214-8.	Surveys	44 maternity healthcare workers from 33 NHS hospitals in England, including 14 midwives, 3 senior midwives, 2 senior trainee obstetricians, 21 consultant obstetricians, 1 clinical fellow, 1 service director, 1 labour ward coordinator and 1 clinical midwifery manager	November 2020 - July 2021	Mid pandemic	England	Testing and isolation	Patient participation in testing programme.
10	Isherwood KR, Kyle RG, Gray BJ, Davies AR. Challenges to self-isolation among contacts of cases of COVID-19: a national telephone survey in Wales. J Public Health (Oxf). 2022.	Surveys	2027 - Individuals were eligible for inclusion if they: (i) had been successfully contacted by TTP after forward contact tracing (ii) were a close contact of a confirmed case of COVID-19; (iii) were aged 18 years or over; (iv) resident in Wales: and (v) had completed their self-isolation period at the time of telephone survey. Contacts were excluded from the study if they were: (i) under the age of 18; (ii) currently self-isolating; (iii) not a resident in Wales	11 November - 1 December 2020	Mid pandemic	Wales	Isolation	Daily contact testing rather than asymptomatic testing – focus on attitudes towards contact testing as an alternative to contacts of positive cases having to isolate. Before Universal Testing
11	Jayes L, Bogdanovica I, Johnston E, Chattopadhyay K, Morling JR, Devine S, Richmond N, Langley T. Perspectives of attenders and non-attenders to SARS-CoV-2 asymptomatic community testing in England: a qualitative interview study. BMJ Open. 2022;12(9):e064542.	Interviews	With 18 members of the public who attended a community testing centre and 15 who had not	February 2021 - May 2021	Mid pandemic	England	Isolation	Community asymptomatic testing. Study period covers before and after the start of introduction of Universal Testing.

12	Knight H, Carlisle S, O'Connor M, Briggs L, Fothergill L, Al-Oraibi A, Yildirim M, Morling JR, Corner J, Ball J, Denning C, Vedhara K, Blake H. Impacts of the COVID-19 Pandemic and Self-Isolation on Students and Staff in Higher Education: A Qualitative Study. Int J Environ Res Public Health. 2021;18(20). Epub 20211012. doi: 10.3390/ijerph182010675.	Interview and focus groups	26 university staff interviews and 11 university students participating in focus groups	January 2021 - March 2021	Mid pandemic	England	Testing and isolation	University testing programme. Before the introduction of Universal Testing.
13	Mahmood F, Acharya D, Kumar K, Paudyal V. Impact of COVID-19 pandemic on ethnic minority communities: a qualitative study on the perspectives of ethnic minority community leaders. BMJ Open. 2021;11(10):e050584.	Interviews	19 - Community leaders recruited through organisations representing ethnic minority communities and religious places of worship	October - November 2020	Mid pandemic	England	Testing, reporting and isolation	Community testing focussing on ethnic minority communities. Before Universal Testing.
14	Martin AF, Denford S, Love N, Ready D, Oliver I, Amlot R, Rubin GJ, Yardley L. Engagement with daily testing instead of self-isolating in contacts of confirmed cases of SARS-CoV-2. BMC Public Health. 2021;21(1):1067.	Surveys	319 people who had agreed to daily testing 205 who were not offered daily testing - adult contacts of confirmed COVID-19 cases who consented to daily testing, and a comparison group of contacts who were not offered testing and instead self-isolated	11 – 23 December 2020 and 4 - 12 January 2021	Mid pandemic	England	Testing and isolation	Daily contact testing rather than asymptomatic testing – focus on attitudes towards contact testing as an alternative to contacts of positive cases having to isolate. Before Universal Testing.
15	Mowbray F, Woodland L, Smith LE, Amlot R, Rubin GJ. Is My Cough a Cold or Covid? A Qualitative Study of COVID-19 Symptom Recognition and Attitudes Toward Testing in the UK. Front Public Health. 2021;9:716421.	Interviews	40 people (21 members of the general population, 19 students)	30 November - 11 December 2020	Mid pandemic	England	Testing	Focus on symptom recognition to test with a mixture of general public and university students. Before Universal Testing.
16	Robin C, Symons C, Carter H. Local Community Response to Mass Asymptomatic COVID-19 Testing in Liverpool, England: Social Media Analysis. JMIR Form Res. 2022;6(8):e34422.	Other – review of media and social media comments	Overall, 1096 comments were sampled: 219 newspapers comments, 472 Facebook comments, and 405 tweets. - Members of Liverpool local community	2 November 2020 - 8 November 2020	Mid pandemic	England	Isolation	Social media analysis of responses and views towards community asymptomatic testing. Before Universal Testing.

17	Smith LE, Amlot R, Lambert H, Oliver I, Robin C, Yardley L, Rubin GJ. Factors associated with adherence to self-isolation and lockdown measures in the UK: a cross-sectional survey. Public Health. 2020;187:41-52.	Surveys	Adults in the UK recruited online - 217 with experience of isolation of 2240 total respondents	6 - 7 May 2020	Early pandemic	UK	Isolation	Focus on adherence to isolation rather than on testing. Relatively early in the pandemic – first lockdown. Before Universal Testing.
18	Smith LE, Potts HWW, Amlot R, Fear NT, Michie S, Rubin GJ. Intention to adhere to test, trace, and isolate during the COVID-19 pandemic (the COVID-19 Rapid Survey of Adherence to Interventions and Responses study). Br J Health Psychol. 2022;27(3):1100-18.	Surveys	12,976 - Selected only participants who lived in England due to differing restrictions across the four UK nations. and were eligible for the study if they were aged 16 years or over and lived in the United Kingdom. Quotas were applied based on age and gender (combined), and reflected targets based on data from the Office for National Statistics	27 April 2020 – 27 January 2021	Early- Mid pandemic	UK	Testing & Isolation	Before Universal Testing.
19	Smith LE, Potts HWW, Amlôt R, Fear NT, Michie S, Rubin GJ. Adherence to the test, trace, and isolate system in the UK: results from 37 nationally representative surveys. BMJ. 2021;372:n608.	Surveys	74 699 responses from 45 957 people living in the UK, aged 16 years or older	2 March 2020 to 27 January 2021	Early- Mid pandemic	UK	Testing, Reporting and Isolation	Levels of adherence to test, trace, and isolate are low, although some improvement has occurred over time. Practical support and financial reimbursement are likely to improve adherence. Targeting messaging and policies to men, younger age groups, and key workers might also be necessary.
20	Street A, Lee SJ, Bevan I. The hidden burden of medical testing: public views and experiences of COVID-19 testing as a social and ethical process. BMC Public Health. 2022;22(1):1837.	Interviews	70 - members of the general public (aged 19–85) living in the Lothian region of Scotland	7 July - 24 September 2020	Early- Mid pandemic	Scotland	Isolation	Before Universal Testing.

21	van der Scheer JW, Ansari A, McLaughlin M, Cox C, Liddell K, Burt J, George J, Kenny R, Cousens R, Leach B, McGowan J, Morley K, Willars J, Dixon-Woods M. Guiding organisational decision-making about COVID-19 asymptomatic testing in workplaces: mixed-method study to inform an ethical framework. BMC Public Health. 2022;22(1):1747.	Surveys and interviews	50 survey participants and 11 interviews with staff members from workplaces used as case studies	November 2020 - December 2020	Mid pandemic	England	Testing and isolation	Exploring how workers feel about workplace testing and the implications of testing and isolation on their work. Before Universal Testing.
**Google/ Bibliographic search**								
1	Atchison C, Bowman LR, Vrinten C, Redd R, Pristerà P, Eaton J, Ward H. Early perceptions and behavioural responses during the COVID-19 pandemic: a cross-sectional survey of UK adults. BMJ Open. 2021;11(1):e043577.	Surveys	Nationally representative sample of 2108 UK adults	17 March 2020 - 18 March 2020	Early pandemic	UK	Isolation	Study conducted within 48 hours of UK government advising public to stop non-essential contact with others and all unnecessary travel
2	Bevan I, Stage Baxter M, Stagg HR, Street A. Knowledge, Attitudes, and Behavior Related to COVID-19 Testing: A Rapid Scoping Review. Diagnostics (Basel). 2021;11(9).	Survey, interview, and focus groups	UK population (amongst other international samples)	March 2020 - January 2021	Early- Mid pandemic	UK	Testing and isolation	Rapid scoping review found existing research at time of review was limited in depth and scope.
3	Denford S, Towler L, Ali B, Treneman-Evans G, Bloomer R, Peto TE, Young BC, Yardley L. Feasibility and acceptability of daily testing at school as an alternative to self-isolation following close contact with a confirmed case of COVID-19: a qualitative analysis. BMC Public Health. 2022;22(1):742.	Interviews	Staff, students, and parents of students (n=63)	Not stated	Mid pandemic?	England	Testing, reporting and isolation	At time of study, close contacts of the case required to self-isolate at home for 10 days.
4	French C, Denford S, Brooks-Pollock E, Wheling H, Hickman M. Low uptake of COVID-19 lateral flow testing among university students: a mixed methods evaluation. Public Health. 2022; 204:54–62.	Surveys, interviews, and focus groups	University students. A total of 436 students completed the online survey, and 20 in-depth interviews were conducted	November - December 2020	Mid pandemic	England	Isolation	Based at one university assessing LFT testing uptake and the differences between demographic variations
5	Kyle RG, Isherwood KR, Bailey JW, Davies AR. Self-isolation confidence, adherence and challenges: Behavioural insights from contacts of cases of COVID-19 starting and completing	Survey, interviews, and focus group	General population with 13,531 responses from survey and 1011 telephone surveys	November 2020 - January 2021	Mid pandemic	Wales	Isolation	Report published by NHS Public Health Wales exploring factors associated to

	self-isolation in Wales. Cardiff, Wales: Iechyd Cyhoeddus Cymru/Public Health Wales, 2021.							adherence to isolation with the aim to inform content of public health messaging and support.
6	Lin L, Song Y, Wang Q, Pu J, Sun FY, Zhang Y, Zhou X, Larson HJ, Hou Z. Public Attitudes and Factors of COVID-19 Testing Hesitancy in the United Kingdom and China: Comparative Infodemiology Study. JMIR Infodemiology 2021;1(1): e26895	Other- Social listening	General population - 3856 UK tweets and 9299 Chinese Sina Weibo posts	1 June - 15 July 2020	Early pandemic	International (including UK)	Testing, reporting and isolation	Comparative analysis between UK and China to assess public attitudes and uptake regarding COVID-19 testing.
7	Love N, Ready D, Turner C, Yardley L, Rubin J, Hopkins S, et al. The acceptability of testing contacts of confirmed COVID-19 cases using serial, self-administered lateral flow devices as an alternative to self-isolation. medRxiv. 2021.	Interviews	General population (n=882)	11 - 23 December 2020 And 4 - 12 January 2021	Mid pandemic	England	Testing, reporting & isolation	Investigating a 'test to enable' approach as part of England's tracing strategy dur to suboptimal adherence to self-isolation guidance.
8	Marshall GC, Skeva R, Jay C, Silva MEP, Fyles M, House T, Davis EL, Pi L, Medley GF, Quilty BJ, Dyson L, Yardley L, Fearon E. Public perceptions and interactions with UK COVID-19 Test, Trace and Isolate policies, and implications for pandemic infectious disease modelling. [version 1; peer review: awaiting peer review] F1000Research. 2022;11:1005.	Interviews	20 adults over the age of 18 living in the UK	Summer 2021	Mid pandemic	UK	Testing, reporting and isolation	Interviews took place during a policy change (19 July 2021), so the findings may be overrepresenting aspects of the interview context. Whilst efforts were made to recruit a diversity of views, participants were necessarily those who chose to take part in this study.
9	Smith LE, West R, Potts HWW, Amlot R, Fear NT, Rubin GJ, Michie S. Knowledge of Self-Isolation Rules in the UK for Those Who Have Symptoms of COVID-19: A Repeated Cross-Sectional Survey Study. Int J Environ Res Public Health. 2023;20(3).	Surveys	78,573 responses from 51,881 participants of the general population (UK adults)	November 2020 - February 2022	Mid- Late pandemic	UK	Isolation	At time of study adherence to self-isolation in people who develop key COVID-19

								symptoms is low in the UK.
10	Street A, Baxter MS, Christison S, Bevan I, Bauld L. Student views and experiences of asymptomatic COVID-19 testing at the University of Edinburgh. DiaDev Investigating Diagnostics in Global Health, The University of Edinburgh, 2021.	Interviews	24 University students who had completed a 10-minute survey	November - December 2020	Mid pandemic	Scotland	Testing and isolation	Main goal at the time of the mass testing programme was to minimise transmission of COVID19 when students travelled home for winter break.
**Stakeholder identified sources (UKHSA Secretariat)**								
1	ICF Consulting Services Limited. Evaluation of the Enduring Transmission Pilot in Peterborough, Fenland and South Holland. 2022.	Interviews	Untreated - 764,686<br>Treated - 228<br>Total - 764,914 - A total of 717 treated people were included in the pilot's MI (individuals supported by Rosmini from June to December 2021). Of these, 504 individuals could be linked to CTAS data based on their postcode, gender, ethnicity, and age (measured at assessment date). Among these 504 treated people, 276 could not be used for analysis for various reasons. Removing these 276 individuals from the sample of 504 treated people linked to the CTAS database resulted in a final sample of 228 treated people.<br><br>The sample of 764,686 untreated people was obtained by considering all individuals aged 18-67 who resided in LADs other than Fenland, Peterborough, and South Holland (after excluding all LADs where other Test & Trace pilots were rolled out), and with non-missing value on all the variables of interest (demographics and outcome).	23 June - 22 November 2021	Mid pandemic	England	Isolation	Evaluation was completed in two stages, a qualitative process evaluation and an impact evaluation stage.
2	Institute for Connected Communities. Newham Council welfare check-in call pilot - Learning from a rapid evaluation 2021–2022: University of East London; 2022. Available from: https://repository.uel.ac.uk/download/6d85e6855	Interviews	41 - The research participants were adult males and females, aged 20 to 75+, from Black, Asian, and ethnic minority<br>groups and White British, from	November - December 2021	Mid -Late pandemic	England	Isolation	'Other' settings.

	9f8c6330ed94896e2165009f6a7bd6bce232452200857a16a7ed2b9/2819325/Welfare%20Check-In%20Call%20Evaluation%20Report.pdf.		across the Borough, and UK nationals and non-nationals. Of the 1,282 who were consented to take part in the study by the Council, 571 were eligible for screening calls. The 41 interviewed participants were clustered around seven postcode areas within the Borough and represented the general demographic characteristics and proportions of Newham. All the research participants who took part in the study had tested positive for COVID-19.					
3	UK Health Security Agency (confidential internal document), Brand perceptions tracker - wave 1. 2020.	Surveys	2,029 adult - Adults aged 18+ in England	9 -17 December 2020	Mid pandemic	England	Testing & isolation	Universal Testing.
4	UK Health Security Agency (confidential internal document).Calls (not texts) work best to encourage self-isolation – results from a field experiment in the Test & Trace programme. 2020.	Other	6,812 trial participants (2652 control, 1219 texts only, 1503 calls only, 1438 texts + calls) - close contacts of confirmed case	17 August - 10 September 2020	Early- Mid pandemic	England	Isolation	Randomised controlled field trial who'd recently been in close contact with a confirmed case. At the time of study, 14-day isolation period required.
5	UK Health Security Agency (confidential internal document). Compliance with self-isolation for those within NHS Test and Trace: Summary of the Evidence. 2021.	Surveys	Exact number hasn't been stated - Surveys were conducted internally and externally as well as analysis of data collected through NHS Test and Trace. Data collection was done from the following sources:<br>1. Internal surveys<br>2. Test and trace data on contacts<br>3. ONS compliance with self-isolation surveys<br>4. Test and Trace compliance	1. Internal Survey<br>a) August - September 2020<br>b) November 2020 - March 2021<br>2) ONS	Early – Mid pandemic	England	Isolation	The issues in obtaining an accurate estimate of compliance include considering the complexities around what is and is not

			check-in calls data<br>5. Test and Trace compliance check-in calls - landline data	compliance surveys – February - March 2021				permitted, including changes over time; the understanding or misunderstanding of the requirements among those who are isolating and the impact this can have on their self-reports. Due to these complexities, it is useful to compare sources of data that use different approaches and methods for obtaining estimates as this can strengthen confidence in their reliability.
6	UK Health Security Agency (confidential internal document). Compliance with self-isolation: publishing findings of ONS commissioned surveys.2021.	Other	n/a - n/a	Not stated	?	England	Isolation	Summary document of the published findings of ONS commissioned surveys to understanding of how well people isolate and what drives compliance.

7	UK Health Security Agency (Confidential internal document), COVID-19 Test and Trace Contacts Behavioural Insights Study - Contacts not required to self-isolate; Wave 1. 2021.	Interviews	1100 adults- aged 18 years or over Respondents were sampled through the Contact Tracing and Advice (CTAS) database, held by NHS TT.	25 – 30 October 2021	Mid pandemic	England	Testing & isolation	Survey for adults who had been in contact with someone who had tested positive for COVID-19 and were not required to self-isolate due to vaccination status.
8	UK Health Security Agency (Confidential internal document), COVID-19 Test and Trace Contacts Behavioural Insights Study - Contacts not required to self-isolate; Wave 2. 2021.	Interviews	918 adults - Respondents were sampled through the Contact Tracing and Advice (CTAS) database, held by NHS TT.	22 – 27 November 2021	Mid pandemic	England	Testing & isolation	Survey for adults who had been in contact with someone who had tested positive for COVID-19 and were not required to self-isolate due to vaccination status.
9	UK Health Security Agency (Confidential internal document), COVID-19 Test and Trace Contacts Behavioural Insights Study - Contacts not required to self-isolate; Wave 3. 2022.	Interviews	1078 - Respondents were sampled through the Contact Tracing and Advice (CTAS) database, held by NHS TT.	10 - 15 January 2022	Late pandemic	England	Testing & isolation	Survey for adults who had been in contact with someone who had tested positive for COVID-19 and were not required to self-isolate due to vaccination status. Strongly advised to take daily LFD for 7 days or until 10 days since their last contact

Keene CM, *et al. BMJ Open* 2024; 14:e084437. doi: 10.1136/bmjopen-2024-084437

								with a positive case if earlier.
10	UK Health Security Agency (Confidential internal document). COVID-19 Test and Trace Contacts Behavioural Insights Study - Contacts not required to self-isolate; Wave 5. 2022.	Interviews	1112 - Respondents were sampled through the Contact Tracing and Advice (CTAS) database, held by NHS TT.	21 - 26 February 2022	Late pandemic	England	Testing & isolation	Survey for adults who had been in contact with someone who had tested positive for COVID-19 and were not required to self-isolate due to vaccination status. Strongly advised to take daily LFD for 7 days or until 10 days since their last contact with a positive case if earlier.
11	UK Health Security Agency (confidential internal document). Daily Testing for Contacts of COVID-19 Cases (DTCC) – Evaluation. 2022.	Surveys	c.9,838 respondents - Customers'	17 December 2021 - 19 January 2022	Late pandemic	England	Testing, reporting & isolation	Interim findings from the evaluation of the Daily Testing of contacts of COVID-19 (DTCC) Question 2: To what extent are people following the guidance to test and acting appropriately on the results of the tests?
12	UK Health Security Agency (confidential internal document). Does mass testing reduce compliance with self-isolation of confirmed positive cases of SARS-CoV-2? Evidence from the Liverpool mass testing pilot. 2021.	Surveys	In total 247 participants with a Liverpool address had participated in the mass testing pilot and 277 tested positive through normal, symptomatic channels. In addition, the study included 3 participants who had participated in the mass testing pilot but did not have a Liverpool address and 678 participants who did not have a Liverpool address and tested positive through normal,	Not stated	Unclear	England	Isolation	There are concerns that participation in asymptomatic community testing may undermine adherence to self-isolation compliance among those who test positive for

			symptomatic channels. - All participants had tested positive for SARS-CoV-2, had their details passed to NHS Test and Trace and had supplied a telephone contact number.					SARS-CoV-2. Regression analysis and propensity score matching was used to assess the impact of participation in the Liverpool mass testing pilot on compliance.
13	UK Health Security Agency (confidential internal document). DTCC (Daily Testing of Covid Contacts) - Voice of the Customer Insight update. 2022.	Surveys	9838 - <customers at the main stages of the test, trace, and isolate journey>	17 December 2021 - 19 January 2022	Late pandemic	England	Testing & isolation	Universal Testing.
14	UK Health Security Agency (confidential internal document). DTCC – Research Findings January 2022. 2022.	Interviews	Unclear - Online focus groups and 18-24 y/o, none following COVID regulations or only following very sporadically	10 January - 17 January 2022	Late pandemic	England	Testing, reporting & isolation	Universal Testing.
15	UK Health Security Agency (confidential internal document). Effect of test and trace support payments scheme on ease of self isolation and isolation compliance- Analysis of ONS Isolation Compliance Survey Data. 2021.	Surveys	12,486 respondents - The analysis uses data from surveys of confirmed positive cases and contacts conducted by ONS and is broken down by characteristics including age, gender, ethnicity, and receipt of any benefits.	March - July 2021	Mid pandemic	England	Isolation	12th October 2020, UK government launched Test and Trace Support Payment (TTSP) scheme (£500 support payment for those needing to isolate and on low incomes).
16	UK Health Security Agency (Confidential internal document). Evaluation of COVID-19 guidance and support for the migrant worker community delivered by voluntary and community sector organisations (VCSOs). 2022.	Interviews	23 service users and 8 stakeholders - Service users (migrant workers) and stakeholders (staff from voluntary and community sector organisations)	29 March - 7 April 2022	Late pandemic	England	Testing & isolation	Previous work by the UKHSA Evaluation team found that Voluntary and community sector organisations (VCSOs) play an important role in delivering support to 'disproportionately impacted' and 'under-served' communities

								such as migrant workers.
17	UK Health Security Agency (confidential internal document). Exploring Compliance with Self-Isolation among Deprived Communities: A Qualitative Research Protocol. 2021.	Interviews	1104 - Deprived communities (postcodes in bottom three deciles Index of Multiple Deprivation)	15 - 19 March 2021	Mid pandemic	England	Isolation	NHS Test and Trace refreshed its business plan in December 2020, reemphasising the importance of breaking the transmission of COVID-19 and highlighting that role contact tracing and self-isolation, of those who have COVID and their close contacts, plays in achieving this aim.
18	UK Health Security Agency (confidential internal document). Impact evaluation of a temporary raise in the wage eligibility threshold for the Test & Trace Support Payment in Bradford. 2021.	Other	Individuals who started a self-isolation period and for whom a compliance outcome is observed (postintervention)= approx. 15,000; individuals who started a self-isolation period = (postintervention) approx. 83,000; individuals who were reached by Test & Trace (postintervention) = approx. 22,000. - Residents of Bradford/other LADs (cases or contacts) who started self-isolating in the pre- or post-intervention period; or those who were reached by Test & Trace via phone in the pre- or post-intervention period; or shared the names of one/more contacts in the pre- or post-intervention period. Note: Only individuals aged between 18 and 67 (working-age population) and who reside in Bradford or one among the other 303 English LADs are included in the analysis	10 April - 19 May 2021 And 1 March - 9 April 2021<br><br>(the 40 days preceding the pilot).	Mid pandemic	England	Testing, reporting & isolation	Between 10th April and 19th May 2021, the wage eligibility threshold for the TTSP was raised in Bradford from £350 to £500 (people within this income range, defined newly eligible individuals, were the main target of the intervention). It was hoped that the intervention would help increase the proportions of individuals claiming and getting TTSP.
19	UK Health Security Agency (Confidential internal document). Isolation Survey Report. 2020.	Surveys	2783 respondents (1818 cases, 965 contacts)	16 November	Early pandemic	England	Isolation	The survey displays

				to 7 December 2020				difference of attitudes and risk perceptions, which were measured in 5 points scale, between self-isolation compliers and non-compliers. The variation can be seen at items how easy to self-isolate, confidence in self-isolation to reduce virus spread, trust of information about Covid-19 from government, and whether government has enacted right measures to protect public. In risk perception, there was a differing perceived risk intensity of Covid-19 to society.
20	UK Health Security Agency (confidential internal document). Kingston Hospital Tracing Pilot-Interim Findings.	Other	Baseline period (March 22nd-June 17th, 2021): 759<br><br>Pilot period (from June 18th-4th November): 2364 - Hospital patients	Baseline period: 22 March -17 June 2021<br><br>Pilot period: 18 June - 4 November 2021	Mid pandemic	England	Reporting & Isolation	In February 2021, we identified that 12% of cases (163 people) that had come through to LCT in Kingston had not completed contact tracing because they were in hospital. To address this gap in contact tracing and offer

								local welfare support to potentially vulnerable resident cases in hospital, Kingston started the Hospital Tracing pilot in June 2021.
21	UK Health Security Agency (confidential internal document). Kirklees Self-isolation Pilot: Evaluation Report. 2021.	Mixed methods	Not clear - Inclusion criteria for the pilot were that individuals:<br><br>1) were required to self-isolate following a positive COVID-19 result; or as a close contact of a confirmed COVID-19 case;<br>2) adequately demonstrated a loss in income due to self-isolation;<br>3) earned £26,000 or less per annum;<br>4) were unable to work from home.	27 May – 22 June 2021	Mid pandemic	England	Testing & isolation	The Kirklees self-isolation pilot is aimed at improving self-isolation outcomes, among other key metrics, using a financial mechanism. The pilot trials a revised version of the Test and Trace Support Payment Scheme (TTSP) with a broader set of eligibility criteria and an income replacement policy. The pilot ran for four weeks between 27 May and 22 June 2021.
22	UK Health Security Agency (confidential internal document). Lambeth Self-isolation Pilot: Impact Evaluation.	Other	not clear - Residents of Lambeth earning up to £30,000 per year were eligible to apply for financial support during self-isolation	Not stated	Unclear	England	Testing & isolation	Universal Testing.
23	UK Health Security Agency (confidential internal document). London Borough of Havering Isolation Outreach Pilot Evaluation.	Mixed methods	not clear - The pilot only visited index cases in the community and didn't visit close contact, those in hospital, social care provision or deceased.	7 June - 18 July 2021	Mid pandemic	England	Isolation	To support residents who were isolating due to testing positive with COVID-19, Havering Council embarked on a

								pilot to visit all positive cases within the community twice across the residents 10-day isolation period
24	UK Health Security Agency (confidential internal document). Office for National Statistics - COVID Test and Trace Contacts Insights Study. Wave 5. 2021.	Interviews	918 - Respondents were sampled through the Contact Tracing and Advice (CTAS) database, held by NHS TT.	4 – 8 May 2021	Mid pandemic	England	Testing & isolation	Who had been in contact with someone who has tested positive for coronavirus (COVID-19) and were at (or near) the end of their 10-day self-isolation period.
25	UK Health Security Agency (confidential internal document). Office for National Statistics - COVID Test and Trace Contacts Insights Study. Wave 6. 2021.	Interviews	968 - Respondents were sampled through the Contact Tracing and Advice (CTAS) database, held by NHS TT.	1 – 5 June 2021	Mid pandemic	England	Testing & isolation	Who had been in contact with someone who has tested positive for coronavirus (COVID-19) and were at (or near) the end of their 10-day self-isolation period.
26	UK Health Security Agency (confidential internal document). Office for National Statistics - COVID Test and Trace Contacts Insights Study. Wave 7a. 2021.	Interviews	990 - Respondents were sampled through the Contact Tracing and Advice (CTAS) database, held by NHS TT.	28 June- 3 July 2021	Mid pandemic	England	Testing & isolation	Who had been in contact with someone who has tested positive for coronavirus (COVID-19) and were at (or near) the end of their 10-day self-isolation period.
27	UK Health Security Agency (confidential internal document). Office for National Statistics – COVID Test and Trace Contacts Insights Study. Wave 7b. 2021.	Surveys	1122 - adults aged 18 or more who has tested positive for Covid-19 and were at the end of their 10-day self-isolation period.	8 - 13 March 2022	Late pandemic	England	Isolation	
28	UK Health Security Agency (confidential internal document). Office for National Statistics - COVID	Interviews	946 - Respondents were sampled through the Contact Tracing and	9 – 16 August 2021	Mid pandemic	England	Testing & isolation	Who had been in contact with someone who

	Test and Trace Contacts Insights Study. Wave 8. 2021.		Advice (CTAS) database, held by NHS TT.					has tested positive for coronavirus (COVID-19) and were at (or near) the end of their 10-day self-isolation period.
29	UK Health Security Agency (confidential internal document). Office for National Statistics - COVID Test and Trace Cases Insights Study: Wave 7. 2021.	Surveys	881- adults aged 18 or more who has tested positive for Covid-19 and were at the end of their 10-day self-isolation period.	27 September - 2 October 2021	Mid pandemic	England	Isolation	
30	UK Health Security Agency (confidential internal document). Office for National Statistics - COVID Test and Trace Cases Insights Study. Wave 8. 2021.	Surveys	976 - adults aged 18 or more who has tested positive for Covid-19 and were at the end of their 10-day self-isolation period.	1 - 6 November 2021	Mid pandemic	England	Isolation	Adults who had tested positive for coronavirus (COVID-19) and who were at the end of their 10-day self-isolation period.
31	UK Health Security Agency (confidential internal document). Office for National Statistics - COVID Test and Trace Self-Isolation Insights Study. Contacts required to Self-isolate. Waves 9 & 10. 2021.	Interviews	n/a - Respondents were sampled through the Contact Tracing and Advice (CTAS) database, held by NHS TT.	October - November 2021	Mid pandemic	England	Testing & isolation	COVID Test and Trace Cases Insights Study aims to understand behaviour of individuals required to self-isolate after testing positive. Universal Testing-survey aimed to exclude those who had received both doses of the vaccine or did not need to isolate.
32	UK Health Security Agency (confidential internal document). Office for National Statistics - COVID Test and Trace Contacts Insights Study. Wave 9. 2021.	Surveys	895 - adults aged 18 or more who has tested positive for Covid-19 and were at the end of their 10-day self-isolation period.	29 November - 4 December 2021	Late pandemic	England	Isolation	Understand the behaviour of individuals required to self-isolate after testing positive for coronavirus (COVID-19), as well as the effects of isolation on

								mental health, work, and finances.
33	UK Health Security Agency (confidential internal document). Office for National Statistics - COVID Test and Trace Cases Insights Study. Wave 11. 2022.	Surveys	1006 - adults aged 18 or more who has tested positive for Covid-19 and were at the end of their 10-day self-isolation period.	7 - 12 February 2022	Late pandemic	England	Isolation	Adults who had tested positive for coronavirus (COVID-19) and who were at the end of their self-isolation period.
34	UK Health Security Agency (confidential internal document). Office for National Statistics - COVID Test and Trace Self-Isolation Insights Study. Contacts required to Self-isolate. Wave 11. 2022.	Interviews	1184 - Respondents were sampled through the Contact Tracing and Advice (CTAS) database, held by NHS TT.	1 December 2021	Late pandemic	England	Testing & isolation	Survey for adults who were required to self-isolate after being in contact with someone who tested positive for COVID-19 and were at (or near) the end of their 10-day self-isolation period. This survey aimed to exclude those who had received both doses of the vaccine or did not need to self-isolate for another reason.
35	UK Health Security Agency (confidential internal document).Office for National Statistics - Indicators of Clinically Extremely Vulnerable Individuals' Responses to COVID-19 Outbreak. COVID High Risk Group Insights. 2021.	Interviews	2979 - Clinically extremely vulnerable populations	18 - 30 January 2021	Mid pandemic	England	Testing & isolation	COVID High Risk Group Insights survey was compiled rapidly in response to policy questions on whether the population who had been advised to shield were following shielding guidance and other information.

36	UK Health Security Agency (confidential internal document). Office for National Statistics - International Arrivals Insights Study: Waves 4 & 5. 2021.	Interviews	1067 - The sample included adults who arrived in the UK on 8 and 11 October (Wave 4) and 5 and 8 (Wave 5).	October - November 2021	Mid pandemic	England	Testing & isolation	Adults who had arrived in the UK from countries or territories that were not on the red list and reported that they were quarantining at an address in England.
37	UK Health Security Agency (confidential internal document). Office for National Statistics - Test and Trace Cases Insights Study. Wave 1: Pilot. 2021.	Surveys	2552 - adults aged 18 or more who has tested positive for Covid-19 and were at the end of their 10-day self-isolation period.	1 - 13 February 2022	Late pandemic	England	Isolation	COVID Test and Trace Cases Insights Study aims to understand behaviour of individuals required to self-isolate after testing positive. Universal Testing.
38	UK Health Security Agency (confidential internal document). Office for National Statistics -Test and Trace Cases Insights Study. Wave 3. 2021.	Surveys	1168 - adults aged 18 or more who has tested positive for Covid-19 and were at the end of their 10-day self-isolation period.	12 - 16 April 2021	Mid pandemic	England	Isolation	
39	UK Health Security Agency (confidential internal document). Office for National Statistics- Test and Trace Cases Insights Study. Wave 4. 2021.	Surveys	1044 - adults aged 18 or more who has tested positive for Covid-19 and were at the end of their 10-day self-isolation period.	10 -15 May 2021	Mid pandemic	England	Isolation	
40	UK Health Security Agency (confidential internal document). Office for National Statistics- Test and Trace Cases Insights Study. Wave 5. 2021.	Surveys	1090- adults aged 18 or more who has tested positive for Covid-19 and were at the end of their 10-day self-isolation period.	7 -12 June 2021	Mid pandemic	England	Isolation	
41	UK Health Security Agency (confidential internal document). Office for National Statistics- Test and Trace Cases Insights Study. Wave 6. 2021.	Surveys	936- adults aged 18 or more who has tested positive for Covid-19 and were at the end of their 10-day self-isolation period.	5 -10 July 2021	Mid pandemic	England	Isolation	
42	UK Health Security Agency (confidential internal document). Serial testing of contacts in the context of institutions. 2020.	Interviews	9 - Full-time, part-time, contractors, freelancers, zero-hour contract who cannot work from home or a mix of working from home and outside.<br><br>Mixed experience with Covid-19 test, trace and self-isolation.	3 – 4 December 2020	Mid pandemic	England	Testing & isolation	Initial objectives included test to enable, improve case identification using LFDs for contacts and enable people to

Keene CM, *et al. BMJ Open* 2024; 14:e084437. doi: 10.1136/bmjopen-2024-084437

								avoid unnecessary self-isolation.
43	UK Health Security Agency (confidential internal document). Public perceptions overview. Data from Public Perceptions Tracker 2021.	Surveys	around 1,000 each week - nationally representative sample of adults aged 18 or more	November 2021 report	Late pandemic	England	Testing & reporting	After a short increase, negative media recall of NHSTT appears to be falling. Trust in testing continues to fluctuate.
44	UK Health Security Agency (confidential internal document). Public Sector Self-Collect Evaluation – (Draft) Final Report.	Survey, interview, and focus group	not clear - Public sector employees	1st March 2021 to 30th June 2021	Mid pandemic	UK	Testing & reporting	This report evaluates the roll-out of Self-Test Collect (STC) in the public sector workplace and how it performed against the existing Assisted Test Sites (ATS) model for the public sector workplace
45	UK Health Security Agency (confidential internal document). Research exploring the barriers and drivers to following guidance. nd.	Focus Groups	6 focus groups, 6 interviews with 18–24-year-olds - X 2 groups of people most engaged with and following guidance<br>X 2 groups of people following guidance in a more Ad-hoc manner, picking and choosing<br>X2 groups of people least engaged with guidance and least likely to follow it<br><br>X6, 60-minute depth interviews with 18-24 y/Os	10th January and w/c 17th January 2022	Late pandemic	England	Testing & reporting	At time of research, long list of guidelines to consider, from when to wear a face covering, vaccinations, testing, isolation and what to do if you are a COVID contact.
46	UK Health Security Agency (confidential internal document). Serial contact testing pilots evaluation summary.	Survey, interview, and focus group	n/a – employees	January - Mach 2021	Mid pandemic	England	Testing & isolation	Evaluation summary document.
47	UK Health Security Agency (confidential internal document). Summary Evaluation Findings: Venue Alerts.	Mixed methods	n/a - Recipients of venue alerts in England	24 September 2020 - 19 July 2021	Mid pandemic	England	Isolation	Venues displayed an NHS QR code and kept a record of contacts details for customers,

								visitors and staff who did not sign in through the NHS Covid-19 app. These allowed people to register their attendance so that if a potential outbreak was later identified, others who were present could be notified of their potential risk with a 'venue alert'.
48	UK Health Security Agency (confidential internal document). Supporting COVID-19 testing and vaccination in a deprived local authority: A case study of Blackpool. 2021	Interviews	8 - Four members of Blackpool public health team, the programme director of a large VCFS organisation and 3 Community Champions	May and June 2021	Mid pandemic	England	Testing	Experiences of those working in Blackpool Council and its public health team in supporting testing and vaccination among vulnerable groups, as well as some of the local responses implemented to overcome challenges
49	UK Health Security Agency (confidential internal document). Test and Trace Contacts Insights Study: Wave 3. nd.	Surveys	1100 - adults aged 18 or more who has tested positive for Covid-19 and were at the end of their 10-day self-isolation period.	1 - 10 April 2021	Mid pandemic	England	Isolation	Who had been in contact with someone who has tested positive for coronavirus (COVID-19) and were at the end of their 10-day self-isolation period.
50	UK Health Security Agency (confidential internal document). Test and Trace Contacts Insights Study: Wave 4. nd.	Surveys	1194 - adults aged 18 or more who has tested positive for Covid-19 and	19 - 24 April 2021	Mid pandemic	England	Isolation	

			were at the end of their 10-day self-isolation period.					
51	UK Health Security Agency (Confidential internal document), Test seeking behaviours; attitudes, barriers and facilitators; Briefing Note, 22.03.21. 2021.	Other	NA: This is a literature revie	NA	NA	UK	Testing	Universal testing
52	UK Health Security Agency (confidential internal document). The effects of negative asymptomatic test result messages on understanding of residual risk and behavioural intentions. 2021.	Surveys	1200 - Online experiment (12-15th March 2021) with a representative sample of the UK population where participants imagined they had taken an LFT as part of an asymptomatic testing programme	12 - 15 March 2021	Mid pandemic	UK	Other	Some receiving a test-negative result from asymptomatic lateral flow testing may misunderstand this to mean no risk of being infectious, resulting in reduced adherence to guidelines Tested whether communicating residual risk information and behavioural implications in negative test result messages affects residual risk understanding and behavioural intentions.
53	UK Health Security Agency (confidential internal document). Understanding Compliance with Self- Isolation: Summary of Survey Findings 2021.	Surveys	42,892 contacts were invited and 6,813 responded. - Over 18 years contacts who were reached and advice by NHS Test and Trace to isolate.	25 August - 14 September 2020	Early – Mid pandemic	UK	Isolation	Universal Testing.
54	UK Health Security Agency (confidential internal document). Understanding experiences of the Test and Trace Support Payment: A Qualitative Follow-up Study. 2021.	Interviews	31 participants - ONS Cases and Contacts Insight Survey participants and were not asked to take part in further research as a result.	1 May 2021	Mid pandemic	England	Isolation	Follow-up qualitative study to the ONS Cases and Contact Insights Survey. Sampled from both confirmed cases

								and contacts of COVID-19.
55	UK Health Security Agency (confidential internal document), Understanding the impact of a COVID-19 outreach programme delivered by voluntary and community sector organisations (VCSOs) for migrant worker communities and the role of VCSOs in communicating public health messaging. 2022.	Interviews	23 service users and 8 staff members from 4 VCSOs - Voluntary and community sector organisation (VCSO) service users and stakeholders.	17 January - 31 March 2022	Late pandemic	England	Testing & isolation	Aimed to communicate coronavirus (COVID-19) guidance on testing, self-isolation, and vaccination to migrant worker communities to improve understanding and encourage uptake.
56	UK Health Security Agency (confidential internal document). Weekly TIEB updates. 12 March 2021. 2021.	Survey, interview, and focus group	n/a - Private, Public sectors employees, NHS employees	8 - 12 March 2021	Mid pandemic	England	Testing & isolation	Pilots' evaluation update document covering operational feasibility, behaviours, socioeconomic impact.
57	UK Health Security Agency (confidential internal document). YouGov- NHS Test and Trace W50 & W51. 2023.	Surveys	2222 adults - England adults (aged 18+)	6 - 19 December 2021	Late pandemic	England	Testing, reporting & isolation	This survey has been conducted using an online interview administered to members of the YouGov Plc UK panel of 800,000+ individuals who have agreed to take part in surveys.
**Schools setting**								
**Literature search**								
1	Chantziara S, Brigden LCA, McCallum CH, Craddock IJ. Using Digital Tools for Contact Tracing to Improve COVID-19 Safety in Schools: Qualitative Study Exploring Views and Experiences Among School Staff. JMIR Form Res. 2022;6(11):e36412.	Interviews	18 staff (4 senior management, 12 teachers, 1 teaching assistant, and 1 behaviour support manager)	4 June - 7 November 2020	Early- Mid pandemic	England	Other	Background to the study was whether schools required support to implement guidelines.

2	Woodland L, Mowbray F, Smith LE, Webster RK, Amlôt R, Rubin GJ. What influences whether parents recognise COVID-19 symptoms, request a test and self-isolate: A qualitative study. PLoS One. 2022;17(2):e0263537.	Interviews	18 parents of school-aged (4 to 18 years) children	30 November – 11 December 2020	Mid pandemic	England	Testing & isolation	Parents have additional responsibility of using test and trace systems on behalf of their children. Factors associated to use of England's NHS Test and Trace service.
**Evidence overlapping between general and schools service settings**								
1	Nyashanu M, Pfende F, Ekpenyong M. Exploring the challenges faced by frontline workers in health and social care amid the COVID-19 pandemic: experiences of frontline workers in the English Midlands region, UK. J Interprof Care. 2020;34(5):655-61.	Interviews	40 health and social care frontline workers, including 15 nurses, 10 managers and 15 support workers in the English Midlands	February 2020 to April 2020	Early pandemic	England	Testing	Data collected early in the pandemic before asymptomatic testing of healthcare workers was in place, and healthcare workers and patients were prioritised for symptomatic PCR testing
2	Watson D, Baralle NL, Alagil J, Anil K, Ciccognani S, Dewar-Haggart R, Fearn S, Groot J, Knowles K, Meagher C, McGrath C, Muir S, Musgrove J, Glyn-Owen K, Woods-Townsend K, Mortimore A, Roderick P, Baird J, Inskip H, Godfrey K, Barker M. How do we engage people in testing for COVID-19? A rapid qualitative evaluation of a testing programme in schools, GP surgeries and a university. BMC Public Health. 2022;22(1):305.	Interviews and focus groups	210 participants from 4 schools, 1 university and 2 general practices in the South East of England, participating in the Southampton COVID-19 Testing Pilot Programme: 8 general practice staff, 30 pupils, 21 school staff, 12 pupil/parent pairs, 13 parents, 12 senior school representatives, 81 university students, 28 university staff and 5 senior university representatives<br><br>In total: 77 interviews and 20 focus groups	4 June 2020 - 07 November 2020	Early Mid pandemic	England	Testing and isolation	Spoke to i) individuals who had been approached about taking part in the pilot Saliva Testing Programme including some who tested positive; ii) those who were approached but declined to take part; and iii) senior university, primary care and school representatives responsible for delivering the

								Saliva Testing Programme in their organisations.
**Google/ Bibliographic review- Schools**								
1	Godfrey K, Bagust L, Baird J, Barker M, Batchelor J, Bryant S. Evaluation of the expanded Southampton pilot study (Phase 2) for use of saliva-based lamp testing in asymptomatic populations: Final report, 16th November 2020. 2020.	Interviews and focus groups	Staff, students/pupils, and contractors at four schools (an infant, junior, primary, and secondary) n= 88 and at the University of Southampton were included n= 108	September 2020 - November 2020	Mid pandemic	England	Testing, reporting & Isolation	Purpose of the report is to describe the programme and provide a form of manual as guidance for groups undertaking similar testing.
**Stakeholder identified sources (UKHSA Secretariat)**								
1	UK Health Security Agency (confidential internal document). 2021. Secondary schools research - Wave 2, Birmingham 2021.	Focus groups	Mini groups of parents, teachers, and pupils from a range of schools in Birmingham which either encouraged testing or not	November 2021	Mid pandemic	England	Testing, reporting & isolation	Focus is on schools where testing is less encouraged, and parents/children are not testing regularly.
2	UK Health Security Agency (confidential internal document). 2020. School pilots experience research - User research insights and recommendations.	Survey, Interviews and focus groups	Conducted in-depth interviews with participating school staff, pupils, and parents across 9 schools. 15 in-depth interviews and 796 survey respondents	October - December 2020	Mid pandemic	England	Testing, reporting & isolation	Study conducted alongside school mass testing pilots.
**Healthcare**								
**Literature search**								
1	Martindale AM, Pilbeam C, Mableson H, Tonkin-Crine S, Atkinson P, Borek A, Lant S, Gobat N, Solomon T, Sheard S. Perspectives on COVID-19 testing policies and practices: a qualitative study with scientific advisors and NHS health care workers in England. BMC Public Health. 2021;21(1):1216.	Interviews	24 interviews with 13 participants: five senior scientific advisors (all male Caucasian) and eight healthcare workers (five male, four females, all Caucasian) including nurses, physicians, general practitioners, and allied healthcare workers	March - August 2020	Early pandemic	England	Testing, reporting and isolation	Focus on perceptions of healthcare workers and scientific advisors, with data collected early in the pandemic before asymptomatic testing of healthcare workers was in place, and

								healthcare workers and patients were prioritised for symptomatic PCR testing.
2	Vindrola-Padros C, Andrews L, Dowrick A, Djellouli N, Fillmore H, Bautista Gonzalez E, Javadi D, Lewis-Jackson S, Manby L, Mitchinson L, Mulcahy Symmons S, Martin S, Regenold N, Robinson H, Sumray K, Singleton G, Syversen A, Vanderslott S, Johnson G. Perceptions and experiences of healthcare workers during the COVID-19 pandemic in the UK. BMJ Open. 2020;10(11):e040503.	Mixed methods	30 interviews with healthcare staff members<br>101 newspaper articles and 146 000 social media posts to capture the direct or indirect perceptions and experiences of healthcare workers	December 2019 to the end of April 2020<br><br>Interviews took place in April 2020	Early pandemic	UK	Testing	Focus on healthcare worker perceptions and experiences during the first wave, with data collected before asymptomatic testing was available but healthcare workers and patients were prioritised for PCR testing
**Stakeholder identified sources (UKHSA Secretariat)**								
1	Bow SMA, Goddard A, Cope G, Sharp N, Schick J, Woods C, Jeffery K, Harrington D, Williams S, Rodger AJ, Finer S, Fowler T, Hopkins S, Tunkel SA. An evaluation of a pilot of daily testing of SARS-CoV-2 contacts in acute hospital and ambulance trusts in England. Public Health. 2022;209:46-51.	Surveys and interviews	60 healthcare workers completed the survey (58 who did daily contact testing and 2 who did not).<br>28 interviews included trust daily contact testing leads, a union representative and two participants per trust. Healthcare workers were from four large, multisite acute hospital trusts in London, Oxford and Lancashire and a London ambulance trust	9 January - 28 February 2021	Mid pandemic	England	Testing a nd isolation	Data collected within the evaluation period when asymptomatic testing of healthcare workers was in effect.<br><br>This paper focuses on daily contact testing rather than routine asymptomatic testing.
**ASC academic papers**								
**Literature search**								
1	Kierkegaard P, Micocci M, McLister A, Tulloch JSP, Parvulescu P, Gordon AL, Buckle P. Implementing lateral flow devices in long-term care facilities: experiences from the Liverpool	Interviews	15 staff from 9 care homes<br>~ Broad representation of staff working in a range of care homes (residential and nursing homes)	December 2020 –	Mid pandemic	England	Testing	Pilot that largely mirrors protocols

	COVID-19 community testing pilot in care homes- a qualitative study. BMC Health Serv Res. 2021;21(1):1153.		~ Staff who had received training on how to use the LFDs, were directly involved in working with the LFDs to administer visitor and staff testing, and had been working at the care homes prior to the first national lockdown in March 2020, with the rationale that such staff would have a longitudinal perspective from working before and during the pandemic, on how different testing regimes had influenced care home work, and vice versa.	January 2021				adopted in the UK at that time Sample focused on nursing homes Same time as initial vaccine rollout
2	Micocci M, Gordon AL, Allen AJ, Hicks T, Kierkegaard P, McLister A, Walne S, Hayward G, Buckle P. COVID-19 testing in English care homes and implications for staff and residents. Age Ageing. 2021;50(3):668-72.	Interviews	10 staff members from eight care homes in England —with more than 5 years' experience in the sector	July – August 2020	Early pandemic	England	Testing and reporting	The paper describes PCR testing and the feasibility of point of care testing to support the complexity of testing and detection of COVID-19.
3	Tulloch JSP, Micocci M, Buckle P, Lawrenson K, Kierkegaard P, McLister A, Gordon AL, García-Fiñana M, Peddie S, Ashton M, Buchan I, Parvulescu P. Enhanced lateral flow testing strategies in care homes are associated with poor adherence and were insufficient to prevent COVID-19 outbreaks: results from a mixed methods implementation study. Age and Ageing. 2021;50(6):1868-75.	Interviews	15 care home staff members (from 9 care homes)	December 2020 - January 2021	Mid pandemic	England	Testing and Isolation	Pilot that largely mirrored protocols adopted in the UK at that time. Sample focused on nursing homes Same time as initial vaccine rollout
**Google/ Bibliographic review**								
1	Nyashanu M, Pfende F, Ekpenyong MS. Triggers of mental health problems among frontline healthcare workers during the COVID-19 pandemic in private care homes and domiciliary care agencies: Lived experiences of care workers in the Midlands region, UK. Health Soc Care Community. 2022;30(2):e370-e6.	Interviews	40 semi-structured interviews with frontline workers from nursing homes and domiciliary care agencies	Not stated - but before May 2020	Early pandemic	England	Testing & Isolation	Early stages of pandemic, frontline health workers and patients adversely affected.

**Stakeholder identified sources (UKHSA Secretariat)**								
1	UK Health Security Agency (confidential internal document). 2022. Adult Social Care Vaccination & COVID-19 Testing Report.	Surveys	651 adult social care workers including those in care homes, domiciliary and supported living	27 April – 16 May 2022	Late pandemic	England	Testing, reporting, and isolating	Survey conducted following removal of vaccinations as requirement for deployment in adult social care. Further testing changes in April 2022 (outside of evaluation period).
2	UK Health Security Agency (confidential internal document). nd. Baselining the asymptomatic staff testing journey in adult social care (ASC)	Other	Spoke to stakeholders (# undefined) to create high-level and detailed end-to-end service maps<br>Reviewed existing research to identify user needs and mapped user pain points<br>Created a content inventory of adult social care testing guidance and plotted key documents onto the service map<br>Conducted a SWOT analysis (strengths, weaknesses, opportunities, and threats) to identify gaps and opportunities	Undefined	Unclear	England	Testing and reporting	Non-specific as timeline not known.<br>Expected that due to detail, this is after introduction of twice weekly LFD + PCR testing
3	RWD002 Report: Root cause analysis of observed sensitivity of LFDs below that of pre-deployment expected baseline performance when used by Adult Social Care staff	Other	Dual tests data collected between December 2020 and December 2021<br>Reconciled and analysed to explore differences (if any) device sensitivity within the population user group<br>Reviewed Innova and Biotime LFD devices	24 December 2020 – 23 December 2021	Mid pandemic	England	Reporting and Isolation	Generally, when staff followed testing regimen, LFD sensitivity matched baseline expectation. Recognition that funding was available to support self-isolation, with acknowledgemen t that decisions made at LA level are not reviewed.

Supplemental Table 5. Definitions of the stages of the pandemic

Stage	Date range	Notable events
**1. Early stage pandemic**	February 2020 to August 2020	• First lockdown<br>• Introduction of self-isolation rules<br>• Access to PCR testing established and gradually increased<br>• Restrictions from first lockdown eased
**2. Mid-stage pandemic**	September 2020 to November 2021	• Increasing restrictions leading to two more national lockdowns<br>• NHS COVID app launches<br>• Introduction of COVID-19 vaccines<br>• Access to lateral flow device self-test kits for general population<br>• Four-stage roadmap to easing restrictions leading to most legal limits removed by end of July 2021
**3. End-stage pandemic**	December 2021 to March 2022	• Omicron variant spread leads to increase in vaccine boosters, and self-isolation rule changes<br>• February 2021 sees the removal of legal requirement to self-isolate (unvaccinated) and an end to contact tracing

Supplemental Figure 1. Infographic to define the stages of the pandemic, with details.

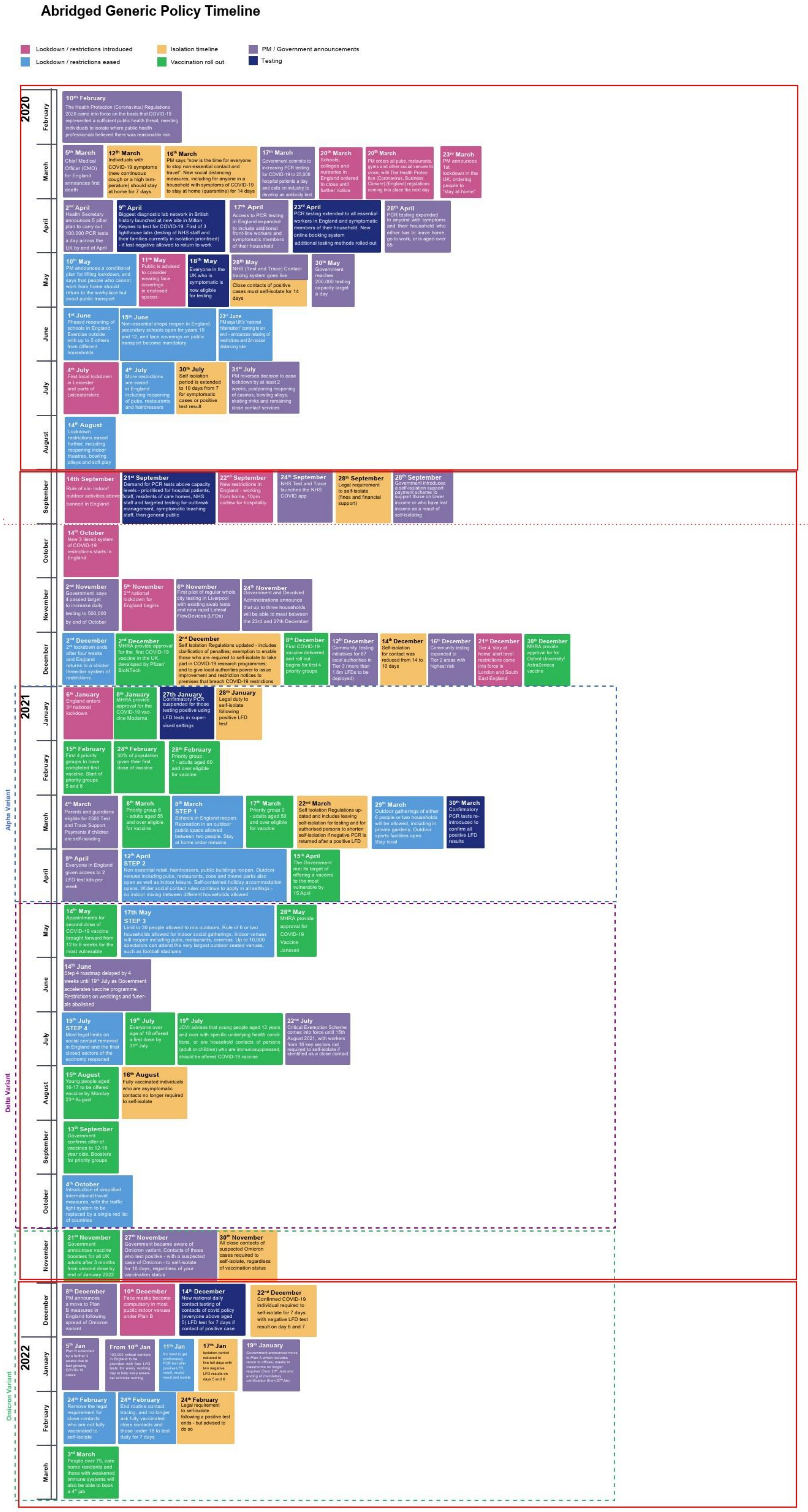

1. Early stage Pandemic

- First lockdown
- Introduction of self-isolation rules
- Access to PCR testing established and gradually increased
- Restrictions from first lockdown eased

2. Mid-stage Pandemic

- Increasing restrictions leading to two more national lockdowns
- NHS COVID app launches
- Introduction of COVID vaccines
- Access to LFD self-test kits for general population
- 4 stage roadmap to easing restrictions leading to most legal limits removed by end of July 2021

3. End stage of pandemic

- Omicron variant spread leads to increase in vaccine boosters and self isolation rule changes
- February 2021 sees the removal of legal requirement to self isolate (unvaccinated) and an end to contact tracing

Keene CM, *et al. BMJ Open* 2024; 14:e084437. doi: 10.1136/bmjopen-2024-084437

Supplemental References

1. National Center for Biotechnology Information (NCBI). PubMed Overview: NCBI; 2023 [cited 2023 28 August]. Available from: https://pubmed.ncbi.nlm.nih.gov/about/.
2. Elsevier. Scopus. Your brilliance, connected: Elsevier; 2022 [cited 2023 28 August]. Available from: https://www.elsevier.com/en-gb/solutions/scopus.
3. Jose Garnica C. The WHO COVID-19 Research Database: behind the scenes of a super powerful tool! Library Science Talk2022.
4. Hanneke CR, Asada Y, Lieberman L, Neubauer LC, Fagen M. The Scoping Review Method: Mapping the Literature in " Structural Change " Public Health Interventions. SAGE Research Methods Cases Part 2. 2017. doi: https://dx.doi.org/10.4135/9781473999008.
