# Supplementary material for: Decision to self-isolate during the COVID-19 pandemic in the UK: a rapid scoping review
Source: BMJ Open. 2024 Mar 29;14(3):e084437. doi: 10.1136/bmjopen-2024-084437 (PMC10982762; doi:10.1136/bmjopen-2024-084437)
Supplement: Author's manuscript [file bmjopen-2024-084437.draft_revisions.pdf]

BMJ Open is committed to open peer review. As part of this commitment we make the peer review history of every article we publish publicly available.

When an article is published we post the peer reviewers' comments and the authors' responses online. We also post the versions of the paper that were used during peer review. These are the versions that the peer review comments apply to.

The versions of the paper that follow are the versions that were submitted during the peer review process. They are not the versions of record or the final published versions. They should not be cited or distributed as the published version of this manuscript.

BMJ Open is an open access journal and the full, final, typeset and author-corrected version of record of the manuscript is available on our site with no access controls, subscription charges or pay-per-view fees (http://bmjopen.bmj.com).

If you have any questions on BMJ Open's open peer review process please email info.bmjopen@bmj.com

BMJ Open

Decision to self-isolate during the COVID-19 pandemic in the UK: a rapid scoping review

Journal:	*BMJ Open*
Manuscript ID	bmjopen-2024-084437
Article Type:	Original research
Date Submitted by the Author:	18-Jan-2024
Complete List of Authors:	Keene, Claire; University of Oxford, Nuffield Department of Medicine<br>Dickinson, Sophie; EY-Oxford Health Analytics Consortium<br>Naidoo, Reshania; University of Oxford, Nuffield Department of Medicine<br>Andersen-Waine, Billie; EY-Oxford Health Analytics Consortium<br>Ferguson-Lewis, Angus; EY-Oxford Health Analytics Consortium<br>Polner, Anastasia; EY-Oxford Health Analytics Consortium<br>Amswych, Ma'ayan; EY-Oxford Health Analytics Consortium<br>White, Lisa ; University of Oxford, Department of Biology<br>Molyneux, Sassy ; University of Oxford, Nuffield Department of Medicine; University of Oxford<br>Wanat, Marta; University of Oxford, Nuffield Department of Primary Care Health Sciences<br>EY-Oxford Health Analytics Consortium, NA; EY-Oxford Health Analytics Consortium
Keywords:	COVID-19, Behavior, Public health < INFECTIOUS DISEASES, Protocols & guidelines < HEALTH SERVICES ADMINISTRATION & MANAGEMENT

SCHOLARONE™
Manuscripts

BMJ

I, the Submitting Author has the right to grant and does grant on behalf of all authors of the Work (as defined in the below author licence), an exclusive licence and/or a non-exclusive licence for contributions from authors who are: i) UK Crown employees; ii) where BMJ has agreed a CC-BY licence shall apply, and/or iii) in accordance with the terms applicable for US Federal Government officers or employees acting as part of their official duties; on a worldwide, perpetual, irrevocable, royalty-free basis to BMJ Publishing Group Ltd ("BMJ") its licensees and where the relevant Journal is co-owned by BMJ to the co-owners of the Journal, to publish the Work in this journal and any other BMJ products and to exploit all rights, as set out in our licence.

The Submitting Author accepts and understands that any supply made under these terms is made by BMJ to the Submitting Author unless you are acting as an employee on behalf of your employer or a postgraduate student of an affiliated institution which is paying any applicable article publishing charge ("APC") for Open Access articles. Where the Submitting Author wishes to make the Work available on an Open Access basis (and intends to pay the relevant APC), the terms of reuse of such Open Access shall be governed by a Creative Commons licence – details of these licences and which Creative Commons licence will apply to this Work are set out in our licence referred to above.

Other than as permitted in any relevant BMJ Author's Self Archiving Policies, I confirm this Work has not been accepted for publication elsewhere, is not being considered for publication elsewhere and does not duplicate material already published. I confirm all authors consent to publication of this Work and authorise the granting of this licence.

For peer review only

Decision to self-isolate during the COVID-19 pandemic in the UK: a rapid scoping review

Co-authors: Claire M Keene[1,†], Sophie Dickinson[2], Reshania Naidoo[2,3], Billie Andersen-Waine[2], Angus Ferguson-Lewis[2], Anastasia Polner[2], Ma'ayan Amswych[2], Lisa J White[4], Sassy Molyneux[1,5], Marta Wanat[6], and the EY-Oxford Health Analytics Consortium[*]

[1] Health Systems Collaborative, Oxford Centre for Global Health Research, Nuffield Department of Medicine, University of Oxford, Oxford, UK
[2] Ernst & Young (EY) UKI Health Sciences and Wellness, London, UK
[3] Centre for Tropical Medicine & Global Health, Nuffield Department of Medicine, University of Oxford, Oxford, UK
[4] Department of Biology, University of Oxford, Oxford, UK
[5] Pandemic Sciences Institute, University of Oxford, UK
[6] Nuffield Department of Primary Care Health Sciences, University of Oxford, UK

[†]Corresponding author: Claire M Keene: clairekeene@gmail.com
[*]EY-Oxford Health Analytics Consortium membership:

- Ricardo Aguas - OUI
- Ma'ayan Amswych - EY
- Sumali Bajaj - OUI
- Kweku Bimpong - EY
- Adam Bodley - OUI
- Liberty Cantrell - OUI
- Siyu Chen - OUI
- Richard Creswell - OUI
- Prabin Dahal - OUI
- Sophie Dickinson - EY
- Sabine Dittrich - OUI
- Tracy Evans - OUI
- Angus Ferguson-Lewis - EY
- Caroline Franco - OUI
- Bo Gao - OUI
- Rachel Hounsell - OUI
- Muhammad Kasim - OUI
- Claire Keene - OUI
- Ben Lambert - OUI
- Umar Mahmood - EY
- Melinda Mills - OUI
- Ainura Moldokmatova - OUI
- Sassy Molyneux - OUI
- Randolph Ngwafor Anye - OUI
- ared Norman - OUI
- Wirichada Pan-Ngum - OUI
- Sunil Pokharel - OUI
- Anastasiia Polner - EY
- Katarzyna Przybylska - OUI
- Sompob Salamba - OUI
- Sheetal Silal - OUI
- oseph Tsui - OUI
- Merryn Voysey - OUI
- Marta Wanat - OUI

Contact emails for submission:
Sophie Dickinson <Sophie.Dickinson@uk.ey.com>;
Reshania Naidoo <reshania.naidoo@ndm.ox.ac.uk>;
Billie Andersen-Waine <b@billieandersen.com>;
Angus Ferguson-Lewis <Afergusonlewis@gmail.com>;
Anastasiia Polner <Anastasiia.Polner@ey-seren.com>;
Ma'ayan Amswych <Maayan.Amswych@uk.ey.com>;
Lisa J White <lisa.white@biology.ox.ac.uk>;
Sassy Molyneux <smolyneux@kemri-wellcome.org>;
Marta Wanat <marta.wanat@phc.ox.ac.uk>

Word count: 4668 words
3 figures 1 table
Abstract 300/300 words

For peer review only

Abstract

Objective: Testing for COVID-19 was a key component of the United Kingdom's (UK) response to the COVID-19 pandemic. This strategy relied on positive individuals self-isolating to reduce transmission, making isolation the lynchpin in the public health approach. Therefore, we scoped evidence to systematically identify and categorise barriers and facilitators to compliance with self-isolation guidance during the COVID-19 pandemic in the UK, to inform public health strategies in future pandemics.

Design: Scoping review

Search strategy: Key terms were used to search literature databases (PubMed, Scopus, and the World Health Organization COVID-19 Research Database, on 7 November 2022), Google Scholar, and stakeholder-identified manuscripts, ultimately including evidence published in English from UK-based studies conducted between 2020 and 2022.

Data extraction and synthesis: Data were extracted and synthesised into themes, organised broadly into capability, opportunity, and motivation, and reviewed with key stakeholders from the UK Health Security Agency (UKHSA).

Results: We included 105 sources, with 63 identified from UKHSA and used to inform their decision-making during the pandemic. Influences on the decision to comply with isolation guidance were categorised into six themes: perceived ability to isolate; information and guidance; logistics; social influences, including trust; perceived value; and perceived consequences. Individuals assessed these factors in deciding whether or not to comply with guidance and self-isolate.

Conclusions: Decisions to self-isolate after a positive test were influenced by multiple factors, according to individuals' differing beliefs, concerns, priorities, and personal circumstances. To support adherence with self-isolation, future strategies must facilitate meaningful financial, practical and mental health support to allow individuals to overcome the perceived and actual negative consequences of isolating. Leveraging the value in protecting others, building trust with the public, and clear, consistent communication of the purpose and procedures of isolating, will also be critical to the success of any future public health response to a pandemic.

Article summary: strengths and limitations

- This scoping review systematically identifies and categorises available data on a critical element of the COVID-19 response in the United Kingdom: adherence with self-isolation guidance.
- This review primarily draws on evidence from studies that focused on testing during the COVID-19 pandemic, given the paucity of studies specifically addressing isolation behaviours.
- Additionally, the review was conducted over a short period of time by a large team, potentially introducing inconsistencies into the screening and extracting processes.
- Nevertheless, a large body of unpublished and confidential internal data from the UK Health Security Agency was included, which supplemented the limited published literature and which would not otherwise be available for synthesis, general dissemination, and contribution to discussions on future strategies.
- Findings from this review have been submitted as part of a larger report to inform the UK Health Security Agency's future pandemic preparedness planning and the UK COVID-19 Inquiry.

Key words: COVID-19, Test trace and isolate, England, isolation behaviour

For peer review only - http://bmjopen.bmj.com/site/about/guidelines.xhtml

Introduction

Any novel pandemic poses a particular challenge in that definitive pharmaceutical interventions, such as therapeutic medications or preventative vaccines, take time to develop.[1] In the meantime, public health policy is reliant on non-pharmaceutical interventions to reduce transmission and lessen impact.[1 2] In the United Kingdom (UK), the initial response to COVID-19 centred on a spectrum of non-pharmaceutical public health measures aimed at mitigating transmission from COVID-19-positive cases[1 2]: periods of national and local lockdown, social distancing, limits on social gatherings, mandated use of face coverings in public spaces, and self-isolation after a positive COVID-19 test result or contact with a case.[3 4]

To facilitate isolation, mass testing for COVID-19 was required to identify COVID-19-positive individuals and their close contacts.[5] The National Health Service Test and Trace (NHSTT) programme was established in May 2020 to implement testing, tracing and isolation services at scale.[6] This complex intervention was the first programme of its kind in the UK, created and delivered at pace during a period of unprecedented uncertainty.[5] It distributed nearly two billion lateral flow tests and 158 million polymerase chain reaction tests free of charge in England alone, at a reported cost of £25.8 billion, with the aim of preventing population-wide deaths, decreasing morbidity, reducing burdens on healthcare services, and facilitating the re-opening of the economy. The testing programme detected between 26% and 40% of all COVID-19 cases in England.[5]

The ultimate goal of the testing and tracing activities was to facilitate isolation of those who tested positive for COVID-19 and/or who were close contacts of positive cases; it was through the isolation step of the NHSTT programme's cascade that transmission was reduced.[7] Consequently, regardless of the resources invested in encouraging people to test, if people did not isolate when required, the impact of the programme would be undermined. Despite requirements to self-isolate, compliance was often suboptimal and varied across different contexts and over time.[5] It is crucial to understand what drove adherence with isolation guidance, and what barriers were faced, to inform future pandemic response strategies.

To support the development of more robust public health strategies in future, we conducted a scoping review of the available evidence on perceptions and experiences of self-isolation during the COVID-19 pandemic in the UK. We then categorised the barriers and facilitators to self-isolation identified.

Methods

Study design

A rapid scoping study was conducted to summarise the large volume of rapidly generated, heterogenous evidence on isolation during the COVID-19 pandemic in the UK, to identify gaps in this evidence, and to describe the barriers and facilitators to engaging with self-isolation guidance. In conducting this scoping review we followed the 2005 'Arksey and O'Malley' framework[8] (with the adaptations proposed by Levac et al in 2010[9]) and drew on the 2015 Joanna Briggs Institute guidance.[10]

This scoping study was nested in a broader rapid scoping review of barriers and facilitators to engaging with the COVID-19 testing programme, covering testing, reporting, and self-isolation behaviour in the UK. This in turn formed part of a mixed methods programme of work conducted by the EY-Oxford Health Analytics Consortium, aimed at evaluating the UK Health Security Agency's (UKHSA's) COVID-19 testing strategy in England – the protocol and report are available online.[5 11]

For peer review only - http://bmjopen.bmj.com/site/about/guidelines.xhtml

For peer review only

Search strategy and selection of the evidence

The search strategy (see supplemental table 1 for search terms) was developed in close consultation with an information specialist and conducted on 7 November 2022 using PubMed, Scopus, the World Health Organization COVID-19 Research Database, and documents provided by UKHSA. UKHSA-provided documents comprising of a body of published and unpublished research; many of these documents were confidential internal documents relating to work specifically conducted to inform UKHSA's decisions during the pandemic. This search was supplemented through free-text searches on Google Scholar, review of the references of included articles, and stakeholder consultation[12] (see supplemental table 2 for the rationale for database selection).

Table 1. Summary of the search parameters and limits as well as the inclusion and exclusion criteria,[2] categorised according to the 'population, context, concept' search framework[15]

	Inclusion criteria	Exclusion criteria
**SEARCH LIMITS**		
**Language**	Published in English	Published in languages other than English
**Dates**	Published from 2020	Published before 2020
**Methods**	Qualitative or mixed methods studies	Purely quantitative studies
**ELIGIBILITY**		
**Literature**	Journal articles, peer-reviewed material, articles under review, published books and book chapters, other academic research, research commissioned by governments, unpublished reports	Opinion or statement pieces, magazine articles, blog posts
**Population**	England, Northern Ireland, Scotland, Wales, and the islands making up the British Isles	Countries outside the UK, including the Republic of Ireland
**Context (service settings)**	All NHSTT programme service settings, including healthcare workers, adult social care, schools, universities, community, and the universal testing programmes.	Nil
**Concept (key activities)**	Description of isolation behaviour and barriers and/or facilitators to adherence to self-isolation guidance (with a focus on isolating due to a positive COVID-19 test result, but including isolating after exposure to a positive contact).	Not focused on the behaviour of self-isolation

Following the database search, all identified citations were collated and uploaded into Rayyan (a web-based research collaboration platform[13]), and duplicates were removed. Following an initial screening pilot, titles and abstracts were screened by two reviewers against the inclusion criteria for the overall project, including testing, reporting, and isolation behaviour. A sample of ≥20% titles and abstracts were evaluated by a third reviewer to clarify eligibility criteria and ensure consistency of inclusion,[9] calculating a Gwet's first-order coefficient (AC1)[14] to assess agreement. Potentially relevant sources were retrieved in full and reviewed against the eligibility criteria. Disagreements at each stage of the selection process were resolved through discussion; discussions were held with an additional reviewer if no consensus was reached. The evidence included in this paper on isolation behaviour was drawn from the documents included in the overarching review (Table 1).

Data extraction, charting and synthesis

To rapidly process a large volume of documents, a team of 12 reviewers extracted the data. Data extracted included i) study metadata and ii) information about the perceptions, experiences, barriers, and facilitators to each of the key activities (testing, reporting, and isolating). Data were extracted into

For peer review only - http://bmjopen.bmj.com/site/about/guidelines.xhtml

an Excel template, refined through entry of initial sources (supplemental table 3). Each reviewer extracted data from two sources that overlapped with sources reviewed by another team member, to check quality and support discussions to refine the template and eligibility criteria. Given the rapid timelines for the overarching programme of work and the aim of this study to scope the evidence, the articles were not assessed for quality.

The analysis combined deductive and inductive elements, organised across three steps. First, a set of a priori categories was developed based on the expertise of the team and an initial reading of included sources. Initial categories of barriers and facilitators were refined throughout the analysis to ensure they reflected the full dataset. Second, the data were synthesised thematically, resulting in some categories being collapsed and reconfigured. Third, the themes were mapped onto the dimensions of the Capability, Opportunity, and Motivation Model of Behaviour (COM-B)[16] (Figure 1), to facilitate selection of potential intervention strategies.

Stakeholder input

Stakeholders from UKHSA were consulted to identify additional sources of published and unpublished evidence (included as 'stakeholder-identified' sources), sense-check the findings, and help frame the results.

Results

Overview of the evidence

After screening 4152 records, this analysis included 105 sources in the synthesis on self-isolation during COVID-19 (see **Error! Reference source not found.**, full details in supplemental table 4). Of these 105 studies, 29% were identified through the literature database search, 60% from UKHSA stakeholders, and 11% from other sources (bibliographic review and Google search). Overall, 81% were focused on England, 3% on Scotland, 2% on Wales, and none on Northern Ireland; 12% covered the UK in general; and 2% included the UK in a broader international study. Data were collected during the early stages of the pandemic (before August 2020) in 18% of papers, during the middle stages (up to November 2021) in 75%, and during the final stages of restrictions in 18% (supplementary figure 1). Surveys were used in 37% of the sources, interviews in 54%, focus groups in 16%, and other methods in 16%.

Overview of results

We arrived at six themes to categorise the barriers and facilitators that influenced the decision to comply with isolation guidance (**Error! Reference source not found.**): logistics of isolation; social influences and trust; perceived ability to isolate; information and guidance; perceived consequences of complying with isolation; and perceived value of isolating. Ultimately, individuals weighed up these influences in their decision to comply with guidance and self-isolate.

1. Perceived ability to self-isolate

Despite negative descriptions of isolation, many people reported being able to self-isolate in line with contemporary requirements[18-21] and confidence in being able to self-isolate effectively and for the full duration required.[19] However, this varied among people and over time, as well as by the level of adherence to the guidance.[22]

Isolation was described as something that could be prepared for,[23 24] with previous experiences influencing whether and how individuals planned for potential periods of self-isolation.[24]

For peer review only - http://bmjopen.bmj.com/site/about/guidelines.xhtml

> *I was aware that this could happen at any moment in time, more in actual fact during the winter time we had snow up here in the North of England [where] we got cut off for a few days, I've had flu before so I've always got lots of soup, things in the freezer, it's kind of an eventuality so I didn't even have to ask somebody to go shopping for me, I just said right we're on lockdown until the test comes back so we had five or six days in the house where we just stayed at home*. (Parent 4).[24]

External support reduced the need for people to leave home[23] and reduced reports of experiencing isolation as a challenge.[25] Required support included practical help, food, medical supplies, financial aid (including sick pay), and mental health care (such as welfare check-in calls or activities to distract those isolating).[26-30] Neighbourly support was also described as fostering a sense of community[30]; for some university students, the opportunity to isolate together in a single space facilitated mutual support and therefore facilitated self-isolation.[31]

Support came from the government through the Test and Protect system, the Test and Trace Support Payment Scheme,[32] local authority support measures such as shopping assistance,[33 34] the entitlement to sick pay,[35] and universities.[30] Other support came through friends, family, and community networks.[23 24 27 30] Support was described as particularly important for 'younger people, those with precarious incomes and women',[36] as well as people 'living alone or without existing social connections established in the community'.[37] Those with potentially greater need for support were less likely to report having adequate support.[25]

The uptake of existing support programmes was reduced by lack of awareness[32 38-40] and access issues,[32 38] including bureaucratic application processes and stringent eligibility criteria.[38 41] However, expanding eligibility alone had mixed effects on adherence to self-isolation.[42-44]. It was suggested that support should also be widely advertised and proactively signposted through support call-handlers[38 45] and that reassurance was needed to overcome stigma and some people feeling unable to ask for help.[27]

2. Information and guidance: understanding when, where, how, and why to isolate

Poor understanding or confusion about the guidance and about transmission risks was a barrier to isolating effectively[22 26 46-49]; reported lack of clarity tended to be in understanding the rules about leaving home for further COVID-19 testing,[19] understanding the duration of isolation and when to start isolating,[46] understanding whether others in the household should isolate,[31] or whether to isolate if asymptomatic.[26 49] Understanding of guidance decreased when regulations changed.[50] One survey found greater understanding of the guidance among those who did self-isolate than among those who did not comply,[19] but this was not always consistent as experiences were also reported of isolating despite not understanding the guidance.[51 52]

Good communication was described as an enabler of adherence to isolation guidance,[41] including provision of written information in different languages and welfare check-in calls that increased knowledge of the guidance.[28] Communication of specific content was suggested to improve isolation uptake: providing insight into the importance of adherence to self-isolation guidance, demonstrating that it was the norm and that many people were following the guidance, reinforcing trust in the government and NHS programmes, and advertising available practical and financial support.[38 53] On the other hand, uncoordinated and inefficient communication during isolation caused frustration.[27]

For peer review only - http://bmjopen.bmj.com/site/about/guidelines.xhtml

3. Logistics of isolation: convenience and practical challenges

People described various practical challenges to following isolation guidance to the letter,[54] including limited access to testing,[26] unsafe or unsuitable home environments, managing childcare and caring responsibilities,[55] the difficulty of managing multiple positive cases or mixed results within a single household,[28 56] and avoiding other household members becoming sick.[24 46 54] These challenges were magnified in smaller houses and apartments[55] or when households included vulnerable family members.[31] Ultimately it appeared that combinations of these challenges heightened barriers to effective isolation.[28]

> *I think it's unrealistic to isolate within the household. If my daughter gets it, it's unrealistic for her to stay in her room and not come out*. (Parent).[46]

4. Social influences and trust in authorities

People perceived high levels of adherence to self-isolation guidance in the community and higher still in their friends and family.[19] It was suggested that the perception that others were following self-isolation guidance, and that isolating when positive was the 'norm', facilitated self-isolation.[53]

Guidance from authoritative sources influenced adherence to self-isolation.[19] However, this deference to authority decreased over time,[57] and distrust in the government was described as a barrier to self-isolation.[26] Disagreement with government measures,[19] mistrust regarding the accuracy of the NHS COVID-19 app and proximity alerts,[24 55 58] and concern over secure handling of details by NHSTT seemed to be associated with lower adherence to self-isolation guidance.[19] A lack of trust in test results undermined engagement with isolation; the negative consequences of isolation (outlined below) were considered an unacceptable burden if the requirement to self-isolate might be 'unnecessary' if based on a false-positive result. This was a particular concern among healthcare workers[27] and care home staff.[59] Negative publicity may have undermined trust in testing processes and, in turn, willingness to isolate.[55]

> *'Several of those interviewed were concerned about reports of accuracy, concerning the LFDs [lateral flow devices]. There was a particular concern about false-positive tests and its potential to reduce their already fragile workforce in the event that they ended up having to send "non-infectious" employees home to self-isolate because of faulty test results.'*[59]

5. Perceived value in complying with isolation

Perceived benefit influenced the decision to comply with isolation guidance.[26] People described adherence to isolation guidance as 'important'[19] primarily to prevent transmission and protect others.[24 27 60] Adherence to self-isolation even beyond official requirements appeared to be driven by perceived risk of contracting COVID-19 and perceived vulnerability to severe COVID-19.[19 60] For these reasons, it was suggested that adherence to self-isolation would be supported through 'strengthening perceived benefit to self-isolate with messages emphasising its effectiveness'[26] and that the desire to protect others should be leveraged in communication of guidance.[48]

Some healthcare workers valued self-isolation as a break from heavy workloads[61] and an opportunity to 'engage in new hobbies, talk to family and friends by phone or online'[37] and foster a 'sense of community with others in the same situation'.[30]

In contrast, others felt that there was no benefit to isolating or following the rules, with some reporting it to be a 'waste of time',[62] particularly when they considered themselves low risk due to vaccination, had low-risk contacts, or used infection control measures.[63] This was particularly the case

For peer review only - http://bmjopen.bmj.com/site/about/guidelines.xhtml

after a positive contact rather than after a positive test or if they had symptoms.[64-67] For some, perceived risk decreased over the course of the pandemic,[60] and students felt at lower risk because of their younger age.[30] Additionally, asymptomatic individuals who tested positive had lower motivation to isolate than symptomatic individuals testing positive, as observed by call handlers.[31]

6. Perceived consequences of isolation

Daily life

People described the consequences of self-isolation on their daily life, such as the inability to leave one's house, socialise, or use public transport.[68] This made it difficult to maintain routine domestic tasks (such as shopping, accessing healthcare, collecting medications, or exercising), maintain social ties with family and friends, and to engage with education or work.[24 27 28 34 37 46 69 70] Parents also anticipated difficulties balancing work and child care obligations when they, or their children, needed to isolate.[24] These difficulties were described as major challenges to adherence to isolation[25] and stated as a reason for non-adherence to isolation guidance.[25 26 33 49]

People described guilt because their isolation would prevent them from performing their usual roles, meaning they were letting people down by not fulfilling their obligations and were placing an additional burden on work colleagues and household members.[28 54 69 71 72] Healthcare workers described their guilt in increasing the workload of already overworked colleagues when they needed to isolate.[73-75] This 'extra pressure on the remaining staff'[76] had organisational implications on staffing numbers and created an 'ethical dilemma' because of the impact on health service provision.[73 77]

> *I'm a carer for vulnerable adults and would be worried about them receiving care that I could not give if I was isolating* (Survey).[54]

Impact on mental health

People also described negative effects of isolation on mental health,[60 71 78] including loneliness, boredom, lack of interaction with friends and family, the inability to exercise, strain on their interpersonal relationships and harmony within a household, and anxiety over finances.[28 31 34 36 37 46] University students in particular described struggling with losing access to their usual coping mechanisms[37] and that isolation exacerbated existing mental health problems.[31] Parents were concerned that isolating took school students away from their peers, education, and outdoor activities, which risked their mental health.[46]

> *I think mental health was like my biggest challenge. It was very easy to just feel down and not wanting to do things, not feel motivated to either do work or just get out of bed (*Focus group 8, student 5).[37]

The negative impact self-isolating had on mental health was described by others as a main barrier to self-isolation.[27 60] People anticipated that self-isolation would negatively impact their mental health,[37 54] and the unwillingness to self-isolate was exacerbated by people becoming 'fed up' with missing out on social interactions and other activities as the pandemic progressed.[55]

Impact on finances

Existing financial hardship was described as an important barrier to isolating.[25 28 79] In addition, self-isolation created financial hardship that acted as a barrier to reporting positive test results, in order to avoid isolation and its financial consequences.[25 69 80-85] These challenges were in large part ascribed to loss of income through loss of work or wages.[27 54 55 59 78 80 86]

For peer review only - http://bmjopen.bmj.com/site/about/guidelines.xhtml

The consequences of self-isolation for certain groups in society, such as those who were homeless, misusing substances, or involved in the criminal justice system, may have differed from those experienced by the wider population, and circumstances may have made self-isolation more difficult to manage and influenced decisions to isolate for these groups.[34] In particular, the financial consequences and considerations were not uniform, with some reporting that they could absorb the loss of income or worked from home already, while others could not 'afford to isolate'.[20 78 82 86] The financial implications of isolation were described as particularly challenging for those with ethnic minority backgrounds, younger people, women, those on lower or precarious incomes, those unable to work from home while self-isolating, those working in 'shut-down sectors', migrant workers, and those who were self-employed, impacting adherence to self-isolation recommendations in these groups.[21 36 81 86 87]

Discussion

This study describes six categories of factors that influenced the decision to self-isolate in the UK during the COVID-19 pandemic, mapped onto the COM-B framework as: capability (perceived ability and information and guidance), opportunity (logistics and social influences, including trust in authority), and motivation (perceived value and consequences).

Decision to isolate

Ultimately, adherence to isolation guidance was a decision that was re-made over the period of required isolation and as individuals' lives changed. Individuals' adherence to guidance ranged from full adherence at a personal cost,[22 88] to partial adherence where isolation was broken for reasons they believed to be 'essential' or 'acceptable',[24] to not isolating at all despite a positive test result[26 33] (including not disclosing their symptoms or hiding their COVID-19 status[87]), to avoidance of testing in the first place.[87] This spectrum of engagement has been reported for other non-pharmaceutical measures across the COVID-19 and other pandemics, with people reporting a range of intentional and non-intentional non-adherence behaviours,[89] influenced at least in part by 'perceived necessity'.[2]

The decision to isolate was shaped by many factors.[24 26 37 60] These results highlighted that people's decision to comply with isolation guidance was supported when the guidance was clear and plainly communicated, there were social norms to isolate and have trust in authority, there was support for planning and managing isolation, there was a perceived risk to themselves or others, and there was clear perceived value of isolation in protecting others from COVID-19 transmission. However, practical logistical challenges, distrust of tests and the implementing institutions, and the negative consequences of isolating on daily life, mental health, and finances all reduced adherence to isolation.

The factors influencing self-isolation adherence reflect the factors that influenced engagement with other COVID-19 public health measures. In the UK, clarity and consistency of guidance,[90 91] trust in authority,[91] perception of risk,[90] social norms,[92] and 'collective importance'[93] were also found to influence adherence to other COVID-19 guidelines,[90] uptake of vaccination,[93 94] and adherence to other non-pharmaceutical measures such as social distancing.[1 89 91 92] Mistrust in authority was found to explain a large proportion of the variance in vaccination hesitancy in the UK[93] and was repeatedly described in the context of non-adherence to social distancing regulations.[1 91] Adherence to different public health measures during COVID-19 was also linked; for example, those more hesitant to get vaccinated for COVID-19 were also less likely to adhere to social distancing guidelines.[93]

For peer review only - http://bmjopen.bmj.com/site/about/guidelines.xhtml

The desire to protect others was also a common theme facilitating engagement with many public health measures to tackle COVID-19[90 92 95] as well as previous pandemics.[2] Other behaviours required of the public during the COVID-19 response had some individual benefit: measures like vaccination or social distancing had personal benefit for the individual through reduction of the personal severity of disease or likelihood of contracting COVID-19, which increased engagement with vaccination programmes[96] or social distancing guidance.[95] Conversely, there was very little perceived direct individual benefit to self-isolation. Rather, the protection of others was commonly described as a major source of the value of isolation and as a 'fundamental driver of behaviour during the COVID-19 pandemic'.[48] Feelings of altruism resulted in moral obligations to engage in pro-social behaviours[97]; such behaviours are particularly important to curb transmission when uncomfortable and undesirable activities like isolation are required.[91]

The costs of self-isolation, however, were sufficiently high and significant to disincentivise not only adherence to isolation guidance when testing positive but also act as a deterrent from testing for COVID-19 in the first place.[5] Unlike the safety concerns influencing uptake of COVID-19 vaccinations,[93] the concerns around the consequences of self-isolating were centred around the financial, practical, and mental health effects of isolating. The interruption of daily life due to isolation was not only inconvenient[24 28 34 37 46 69 98] but also meant that obligations were not fulfilled, resulting in a transfer of the burden to family, friends, and colleagues and creating feelings of guilt for the person isolating.[28 54 69 71 72] Isolation sometimes meant loss of work or even loss of a job; for many this was too high a price to pay to prevent transmission,[27 54 78 80 86 88] echoing the impacts of the adverse socioeconomic effects of isolation that have made it a less acceptable non-pharmaceutical intervention across other pandemics.[2 99]

Recommendations for future pandemic responses

Meaningful support has been described as a critical component of the strategy to encourage engagement with COVID-19 public health measures.[91] The major barrier to adherence to isolation guidance was the perception of personal consequences; therefore, external financial, practical, and mental health support became an important facilitator of self-isolation.[24 27 34 35 79 82 100] Whether this came from the government, official agencies, or informal networks, tangible support reduced the perception of isolation as a challenge.[25] However, support schemes did not consistently improve isolation adherence,[42 43] and uptake of the support offered was suboptimal.[32 39]

Not all individuals experienced the COVID-19 pandemic in the same way: for example, COVID-19 had a disproportionate effect on more vulnerable groups in the UK, with higher rates of infection, morbidity, and mortality in some ethnic minorities, older people, and those living in deprived areas.[101] Influential factors interact so that the motivation to self-isolate played out differently depending on people's capability and opportunity to isolate. While some found self-isolation easy to manage,[19] others found it unmanageable in various ways.[22 88] Contextual factors such as ethnicity and socioeconomics played an important role in engagement with programmes across different COVID-19 prevention measures,[89 102] for example considerably reducing access to and uptake of vaccination[103 104] and increasing the negative impact of social distancing measures.[101] Therefore, support must be tailored to context and to an individual's specific challenges.[27] Advertising a range of easily accessible financial, practical, and mental health support systems for different levels of requirement, based on individual circumstances as well as the intensity of isolation required, could help to streamline support services and make them more accessible.

Communication was described as a crucial tool to facilitate adherence to self-isolation recommendations.[41] Confusing or rapidly changing guidance was a repeated theme across behavioural

For peer review only - http://bmjopen.bmj.com/site/about/guidelines.xhtml

research conducted during the COVID-19 pandemic in the UK and frequently reported as a barrier to engagement with public health programmes.[90] When guidance does not seem to make practical sense it shifts the onus of decision-making onto individuals, who must make choices within the constraints of their personal priorities and context. Thus, official guidance must be particularly clear and unambiguous.[2] This could improve uptake across public health measures during a pandemic,[90] such as social distancing [1 101] or vaccination.[105]

Beyond clear, consistent guidance to support understanding of when, where, and how to self-isolate[22 26 46-48] and providing information on available support,[32 38-40] it was repeatedly recommended that communication strategies should address people's motivation to comply[99] in order to activate personal norms that facilitate pro-social behaviours and engagement with preventative measures.[90 92 94] It was suggested that this could be achieved through messaging that reinforces trust in the government and programmes, demonstrates that adherence to guidance is the social norm, creates awareness of the consequences of non-adherence to guidance, and highlights the perceived benefits to the wider community to appeal to altruism.[26 38 48 53 99]

Many of the factors influencing the decision to self-isolate may be weighed differently in another pandemic: the value of protecting others may change if a disease were to primarily affect different population groups, such as children, or if the severity differed from that of COVID-19. At the same time, if the negative consequences of isolating were mitigated, or the duration of required isolation was different, this would also shift the balance of the decision. Context matters in people's decision-making about health, and the decision to follow guidance cannot be considered in isolation.[106] Therefore, regardless of the lessons learned during the COVID-19 pandemic and their applicability to future pandemic preparedness, research is needed to understand what affects how people weigh the different factors influencing the decision to isolate and what could be leveraged to encourage people to prioritise the value over the costs in future health emergencies. Additionally, as perceptions and decisions changed over time, measures should be in place to collect real-time data in future pandemics and make decisions reactively.

Strengths and limitations

Most evidence included in this review was concerned with factors influencing the decision to test[5] and thus evidence on isolation behaviour was often drawn from studies with a focus on testing during COVID-19. Additionally, this review was conducted in a short space of time with a large team, so multiple people were involved in screening and extracting data, which could have introduced selection bias. However, it is strengthened by the inclusion of a large body of unpublished and confidential internal data from UKHSA, which was able to supplement the paucity of information available on the factors affecting isolation behaviour in the published literature and would otherwise not be available for synthesis, general dissemination, and contribution to discussions on future strategies.

Conclusions

This review demonstrates that the decision to isolate was a complex and dynamic process influenced by multiple factors. All dimensions of the COM-B behavioural model must be in place for people to comply with isolation guidance: in addition to leveraging the value in protecting others and mitigating the negative consequences to motivate people to self-isolate, future pandemic response policies and programmes will need to ensure that individuals have the capability and opportunity to put that motivation into practice. Communication of guidelines must be clear and consistent; context-appropriate financial, practical, and mental health support is required; and building trust with the public will be critical to ensure the success of any public health response to a pandemic.

For peer review only - http://bmjopen.bmj.com/site/about/guidelines.xhtml

For peer review only

For peer review only - http://bmjopen.bmj.com/site/about/guidelines.xhtml

Additional information

List of tables and figures

Table 2. Summary of the search parameters and limits as well as the inclusion and exclusion criteria,[2] categorised according to the 'population, context, concept' search framework[15]

Figure 1. The dimensions of the Capability, Opportunity, and Motivation Model of Behaviour (COM-B), adapted from McDonagh et al's application to a systematic review of chlamydia testing facilitators.[17]

Figure 2. Preferred Reporting Items for Systematic Reviews and Meta-analyses extension for scoping review (PRISMA-ScR) flow diagram outlining the search and inclusion of the literature.

Figure 3. Overview of the themes describing the influences on people's decision to comply with isolation guidance.

Acknowledgements
We would like to extend our gratitude to the UK Health Security Agency for their invaluable collaboration in this scoping review. Their willingness to provide access to confidential internal documents significantly enriched our research and understanding of isolation behaviours during the COVID-19 pandemic in England. We would like to thank by name the team who helped to organise, screen and extract the data: Ainura Moldokmatova, Firdaus Mohandas, Katie Douglass, Umar Mahmood and Zoe Echanah. We would like to acknowledge the contributions of the scientific, clinical and technical experts who have offered valuable input, advice and support throughout the evaluation and preparation of the overarching report, particularly the contributions of the Scientific Advisory Group. Our thanks also go to the numerous healthcare professionals, policy makers, and community leaders who worked tirelessly through COVID-19 pandemic in England to ensure that patients received quality care and that communities were safe.

Availability of data and materials: Data are included as supplementary material. Further data requests can be directed to the corresponding author.

Patient and Public Involvement statement: This study was performed on a short timeline, with a rapid turnaround time for the report. Additionally, no primary data was collected. Therefore, no public stakeholder engagement was considered. However, stakeholders from UKHSA were consulted to identify additional sources of published and unpublished evidence (included as 'stakeholder-identified' sources), sense-check the findings, and help frame the results.

Author contributions:
Conceptualisation and methodology: CMK, BAW, SM, MW, RN, and LJW.
Analysis, integration, and interpretation: CMK, SD, BAW, AFL, AP, MA, SM, MW, and the members of the EY-Oxford Health Analytics Consortium.
Writing – original draft: CMK and SD.
Writing – review, editing, and approval: RN, BAW, AFL, AP, MA, LJW, SM, MW, and the members of the EY-Oxford Health Analytics Consortium. All named authors read and approved the final manuscript.

Declarations
Ethics approval and consent to participate: Not applicable.

Consent for publication: All authors have consented to publication.

Competing interest: All authors had financial support from the UK Health Security Agency (UKHSA) for the submitted work; EY LLP London has previously received payment for consultancy work and advisory on the NHS Test & Trace response from the UK Department of Health and Social Care, now

For peer review only - http://bmjopen.bmj.com/site/about/guidelines.xhtml

known as the UK Health Security Agency. There are no other relationships or activities that could appear to have influenced the submitted work.

Funding: This study was funded by Secretary of State for Health and Social Care acting as part of the Crown through the UK Health Security Agency (UKHSA), reference number C80260/PRO5331**.**

References

1. Williams SN, Armitage CJ, Tampe T, et al. Public perceptions and experiences of social distancing and social isolation during the COVID-19 pandemic: a UK-based focus group study. *BMJ Open* 2020;10(7):e039334. doi: 10.1136/bmjopen-2020-039334 [published Online First: 20200720]
2. Teasdale E, Santer M, Geraghty AWA, et al. Public perceptions of non-pharmaceutical interventions for reducing transmission of respiratory infection: systematic review and synthesis of qualitative studies. *BMC Public Health* 2014;14(589):1-17. doi: 10.1186/1471-2458-14-589
3. UK Health Security Agency (confidential internal document). Effectiveness of non-pharmaceutical interventions to reduce transmission of COVID-19 in the UK: A rapid mapping review, 2023.
4. Institute for Government Analysis. Timeline of UK government coronavirus lockdowns and measures, March 2020 to December 2021, 2022:1.
5. EY–Oxford Health Analytics Consortium Evaluation of the national COVID-19 testing programme in England between October 2020 and March 2022., 2023:1-427.
6. Department of Health and Social Care. Government launches NHS Test and Trace service 2020 [cited 2023 29 October 2023]. Available from: https://www.gov.uk/government/news/government-launches-nhs-test-and-trace-service accessed 29 October 2023 2023.
7. Kucharski AJ, Klepac P, Conlan AJK, et al. Effectiveness of isolation, testing, contact tracing, and physical distancing on reducing transmission of SARS-CoV-2 in different settings: a mathematical modelling study. *Lancet Infect Dis* 2020;20(10):1151-60. doi: 10.1016/S1473-3099(20)30457-6 [published Online First: 20200616]
8. Arksey H, O'Malley L. Scoping studies: Towards a methodological framework. *International Journal of Social Research Methodology: Theory and Practice* 2005;8(1):19-32. doi: 10.1080/1364557032000119616
9. Levac D, Colquhoun H, O'Brien KK. Scoping studies: Advancing the methodology. *Implementation Science* 2010;5(1):1-9. doi: 10.1186/1748-5908-5-69
10. Peters MD, Godfrey CM, Khalil H, et al. Guidance for conducting systematic scoping reviews. *Int J Evid Based Healthc* 2015;13(3):141-6. doi: 10.1097/xeb.0000000000000050 [published Online First: 2015/07/03]
11. Naidoo R, Andersen-Waine B, Dahal P, et al. A multistage mixed-methods evaluation of the UKHSA testing response during the COVID-19 pandemic in England. *medRxiv* 2022:2022.10.27.22281604. doi: 10.1101/2022.10.27.22281604
12. Hanneke CR, Asada Y, Lieberman L, et al. The Scoping Review Method: Mapping the Literature in " Structural Change " Public Health Interventions. *SAGE Research Methods Cases Part 2* 2017 doi: https://dx.doi.org/10.4135/9781473999008
13. Ouzzani M, Hammady H, Fedorowicz Z, et al. Rayyan-a web and mobile app for systematic reviews. *Systematic Reviews* 2016;5(1):1-10. doi: 10.1186/s13643-016-0384-4
14. Gwet KL. Computing inter-rater reliability and its variance in the presence of high agreement. *British Journal of Mathematical and Statistical Psychology* 2008;61(1):29-48. doi: 10.1348/000711006X126600

For peer review only - http://bmjopen.bmj.com/site/about/guidelines.xhtml

15. Peters MD. In no uncertain terms: the importance of a defined objective in scoping reviews. *JBI Database System Rev Implement Rep* 2016;14(2):1-4. doi: 10.11124/jbisrir-2016-2838 [published Online First: 2016/08/19]
16. Michie S, Stralen MMv, West R. The behaviour change wheel: A new method for characterising and designing behaviour change interventions. *Implementation Science* 2011;6(42):1-11. doi: http://www.implementationscience.com/content/6/1/42
17. McDonagh LK, Saunders JM, Cassell J, et al. Application of the COM-B model to barriers and facilitators to chlamydia testing in general practice for young people and primary care practitioners: a systematic review. *Implement Sci* 2018;13(1):130. doi: 10.1186/s13012-018-0821-y [published Online First: 20181022]
18. UK Health Security Agency (Confidential internal document). Compliance with self-isolation for those within NHS Test and Trace: Summary of the Evidence 2021.
19. UK Health Security Agency (confidential internal document). Isolation_Survey_Report, 2020.
20. Denford S, Towler L, Ali B, et al. Feasibility and acceptability of daily testing at school as an alternative to self-isolation following close contact with a confirmed case of COVID-19: a qualitative analysis. *BMC Public Health* 2022;22(1):742. doi: 10.1186/s12889-022-13204-x [published Online First: 20220413]
21. Atchison C, Bowman LR, Vrinten C, et al. Early perceptions and behavioural responses during the COVID-19 pandemic: a cross-sectional survey of UK adults. *BMJ Open* 2021;11(1):e043577. doi: 10.1136/bmjopen-2020-043577
22. UK Health Security Agency (confidential internal document). COVID-19 Test and Trace Cases Insights Study, Wave 3, 12–16 April 2021, 2021.
23. Smith LE, Amlot R, Lambert H, et al. Factors associated with adherence to self-isolation and lockdown measures in the UK: a cross-sectional survey. *Public Health* 2020;187:41-52. doi: 10.1016/j.puhe.2020.07.024 [published Online First: 20200906]
24. Woodland L, Mowbray F, Smith LE, et al. What influences whether parents recognise COVID-19 symptoms, request a test and self-isolate: A qualitative study. *PLoS One* 2022;17(2):e0263537. doi: 10.1371/journal.pone.0263537 [published Online First: 2022/02/24]
25. Kyle RG, Isherwood KR, Bailey JW, et al. Self-isolation confidence, adherence and challenges: Behavioural insights from contacts of cases of COVID-19 starting and completing self-isolation in Wales. Cardiff, Wales: Iechyd Cyhoeddus Cymru/Public Health Wales, 2021:1-42.
26. Eraso Y, Hills S. Self-Isolation and Quarantine during the UK's First Wave of COVID-19. A Mixed-Methods Study of Non-Adherence. *Int J Environ Res Public Health* 2021;18(13) doi: 10.3390/ijerph18137015 [published Online First: 20210630]
27. Watson D, Baralle NL, Alagil J, et al. How do we engage people in testing for COVID-19? A rapid qualitative evaluation of a testing programme in schools, GP surgeries and a university. *BMC Public Health* 2022;22(1):305. doi: 10.1186/s12889-022-12657-4 [published Online First: 2022/02/16]
28. Institute for Connected Communities. Newham Council welfare check-in call pilot - Learning from a rapid evaluation 2021–2022: University of East London, 2022.
29. UK Health Security Agency (confidential internal document). Calls (not texts) work best to encourage self-isolation - results from a field experiment in the Test & Trace programme, 2020.
30. Knight H, Carlisle S, O'Connor M, et al. Impacts of the COVID-19 Pandemic and Self-Isolation on Students and Staff in Higher Education: A Qualitative Study. *Int J Environ Res Public Health* 2021;18(20) doi: 10.3390/ijerph182010675 [published Online First: 20211012]
31. Godfrey K, Bagust L, Baird J, et al. Evaluation of the expanded Southampton pilot study (Phase 2) for use of saliva-based lamp testing in asymptomatic populations: Final report, 16th November 2020, 2020:120.

For peer review only - http://bmjopen.bmj.com/site/about/guidelines.xhtml

32. UK Health Security Agency (confidential internal document). Effect of test and trace support payments scheme on ease of self-isolation and isolation compliance: Analysis of ONS isolation compliance survey data, 2021.
33. UK Health Security Agency (confidential internal document). Kingston Hospital Tracing Pilot-Interim Findings, nd.
34. Burns J, Mc Goldrick N, Sigerson D, et al. A Health Inequalities Impact Assessment of the surveillance of COVID-19 in asymptomatic patients attending dental settings in Scotland. *Community Dent Health* 2022;39(4):254-59. doi: 10.1922/CDH_00170Burns06 [published Online First: 20221130]
35. UK Health Security Agency (confidential internal document). Test seeking behaviours; attitudes, barriers and facilitators; Briefing Note, 22.03.21, 2021.
36. Isherwood KR, Kyle RG, Gray BJ, et al. Challenges to self-isolation among contacts of cases of COVID-19: a national telephone survey in Wales. *J Public Health (Oxf)* 2022 doi: 10.1093/pubmed/fdac002 [published Online First: 2022/02/09]
37. Al-Oraibi A, Fothergill L, Yildirim M, et al. Exploring the Psychological Impacts of COVID-19 Social Restrictions on International University Students: A Qualitative Study. *Int J Environ Res Public Health* 2022;19(13) doi: 10.3390/ijerph19137631 [published Online First: 20220622]
38. UK Health Security Agency (confidential internal document). Understanding experiences of the Test and Trace Support Payment: A Qualitative Follow-up Study, 2021.
39. UK Health Security Agency (confidential internal document). COVID-19 Test and Trace Cases Insights Study, Wave 4, 10–15 May 2021, 2021.
40. UK Health Security Agency (confidential internal document). COVID-19 Test and Trace Cases Insights Study, Wave 8, 1–6 November 2021, 2021.
41. UK Health Security Agency (confidential internal document). Kirklees Self-isolation Pilot: Evaluation Report 2021.
42. UK Health Security Agency (confidential internal document). Lambeth Self-isolation Pilot: Impact Evaluation 2021.
43. UK Health Security Agency (confidential internal document). Impact evaluation of a temporary raise in the wage eligibility threshold for the Test & Trace Support Payment in Bradford, 2021.
44. UK Health Security Agency (confidential internal document). Kirklees Self-isolation Pilot: Evaluation Report, 2021.
45. UK Health Security Agency (confidential internal document). Evaluation of the Enduring Transmission Pilot in Peterborough, Fenland and South Holland, 2022.
46. UK Health Security Agency (confidential internal document). School pilots experience research - User research insights and recommendations, 2020.
47. UK Health Security Agency (confidential internal document). COVID-19 Test and Trace Contacts Insights Study, Wave 8: 9–16 August 2021, 2021.
48. Smith LE, Potts HWW, Amlot R, et al. Intention to adhere to test, trace, and isolate during the COVID-19 pandemic (the COVID-19 Rapid Survey of Adherence to Interventions and Responses study). *Br J Health Psychol* 2022;27(3):1100-18. doi: 10.1111/bjhp.12576 [published Online First: 20211130]
49. Smith LE, West R, Potts HWW, et al. Knowledge of Self-Isolation Rules in the UK for Those Who Have Symptoms of COVID-19: A Repeated Cross-Sectional Survey Study. *Int J Environ Res Public Health* 2023;20(3) doi: 10.3390/ijerph20031952 [published Online First: 20230120]
50. UK Health Security Agency (confidential internal document). Secondary schools research - Wave 2, Birmingham, 2021.
51. UK Health Security Agency (confidential internal document). COVID-19 Test and Trace Contacts Insights Study, Wave 6: 1–5 June 2021, 2021.
52. UK Health Security Agency (confidential internal document). DTCC (Daily Testing of Covid contacts) - Voice of the Customer insight update.

For peer review only - http://bmjopen.bmj.com/site/about/guidelines.xhtml

53. UK Health Security Agency (confidential internal document). Compliance with self-isolation: publishing findings of ONS commissioned surveys, 2021.
54. van der Scheer JW, Ansari A, McLaughlin M, et al. Guiding organisational decision-making about COVID-19 asymptomatic testing in workplaces: mixed-method study to inform an ethical framework. *BMC Public Health* 2022;22(1):1747. doi: 10.1186/s12889-022-13993-1 [published Online First: 20220915]
55. Marshall GC, Skeva R, Jay C, et al. Public perceptions and interactions with UK COVID-19 Test, Trace and Isolate policies, and implications for pandemic infectious disease modelling. *[version 1; peer review: awaiting peer review] F1000Research* 2022;11:1005. doi: 10.1101/2022.01.31.22269871
56. Bevan I, Stage Baxter M, Stagg HR, et al. Knowledge, Attitudes, and Behavior Related to COVID-19 Testing: A Rapid Scoping Review. *Diagnostics (Basel)* 2021;11(9) doi: 10.3390/diagnostics11091685 [published Online First: 20210915]
57. UK Health Security Agency (confidential internal document). Public perceptions overview, Data from Public Perceptions Tracker , 2021.
58. Chantziara S, Brigden LCA, McCallum CH, et al. Using Digital Tools for Contact Tracing to Improve COVID-19 Safety in Schools: Qualitative Study Exploring Views and Experiences Among School Staff. *JMIR Form Res* 2022;6(11):e36412. doi: 10.2196/36412 [published Online First: 2022/10/04]
59. Kierkegaard P, Micocci M, McLister A, et al. Implementing lateral flow devices in long-term care facilities: experiences from the Liverpool COVID-19 community testing pilot in care homes- a qualitative study. *BMC Health Serv Res* 2021;21(1):1153. doi: 10.1186/s12913-021-07191-9 [published Online First: 20211025]
60. Denford S, Martin AF, Love N, et al. Engagement With Daily Testing Instead of Self-Isolating in Contacts of Confirmed Cases of SARS-CoV-2: A Qualitative Analysis. *Front Public Health* 2021;9:714041. doi: 10.3389/fpubh.2021.714041 [published Online First: 20210803]
61. Bow SMA, Goddard A, Cope G, et al. An evaluation of a pilot of daily testing of SARS-CoV-2 contacts in acute hospital and ambulance trusts in England. *Public Health* 2022;209:46-51. doi: 10.1016/j.puhe.2022.05.013 [published Online First: 20220608]
62. UK Health Security Agency (confidential internal document). Brand perceptions tracker - wave 1, 2020.
63. Denford S, Martin AF, Towler L, et al. A qualitative process analysis of daily contact testing as an alternative to self-isolation following close contact with a confirmed carrier of SARS-CoV-2. *BMC Public Health* 2022;22(1):1373. doi: 10.1186/s12889-022-13800-x [published Online First: 20220718]
64. UK Health Security Agency (confidential internal document). NHS Test and Trace W50 & W51, 2021.
65. UK Health Security Agency. Public perceptions overview, Data from Public Perceptions Tracker , 2021.
66. UK Health Security Agency (confidential internal document). Research exploring the barriers and drivers to following guidance.
67. UK Health Security Agency (confidential internal document). DTCC – Research Findings January 2022, 2022.
68. UK Health Security Agency (Confidential internal document). Public Sector Self-Collect Evaluation – (Draft) Final Report 2021.
69. UK Health Security Agency (confidential internal document). Weekly pilot evaluation update, 12 March 2021, 2021.
70. Street A, Baxter MS, Christison S, et al. Student views and experiences of asymptomatic COVID-19 testing at the University of Edinburgh: DiaDev Investigating Diagnostics in Global Health, The University of Edinburgh, 2021.

For peer review only - http://bmjopen.bmj.com/site/about/guidelines.xhtml

71. Blake H, Knight H, Jia R, et al. Students' Views towards Sars-Cov-2 Mass Asymptomatic Testing, Social Distancing and Self-Isolation in a University Setting during the COVID-19 Pandemic: A Qualitative Study. *Int J Environ Res Public Health* 2021;18(8) doi: 10.3390/ijerph18084182 [published Online First: 20210415]
72. Street A, Lee SJ, Bevan I. The hidden burden of medical testing: public views and experiences of COVID-19 testing as a social and ethical process. *BMC Public Health* 2022;22(1):1837. doi: 10.1186/s12889-022-14217-2 [published Online First: 20220930]
73. Martindale AM, Pilbeam C, Mableson H, et al. Perspectives on COVID-19 testing policies and practices: a qualitative study with scientific advisors and NHS health care workers in England. *BMC Public Health* 2021;21(1):1216. doi: 10.1186/s12889-021-11285-8 [published Online First: 20210624]
74. Nyashanu M, Pfende F, Ekpenyong M. Exploring the challenges faced by frontline workers in health and social care amid the COVID-19 pandemic: experiences of frontline workers in the English Midlands region, UK. *J Interprof Care* 2020;34(5):655-61. doi: 10.1080/13561820.2020.1792425 [published Online First: 20200717]
75. Nyashanu M, Pfende F, Ekpenyong MS. Triggers of mental health problems among frontline healthcare workers during the COVID-19 pandemic in private care homes and domiciliary care agencies: Lived experiences of care workers in the Midlands region, UK. *Health Soc Care Community* 2022;30(2):e370-e76. doi: 10.1111/hsc.13204 [published Online First: 20201026]
76. Vindrola-Padros C, Andrews L, Dowrick A, et al. Perceptions and experiences of healthcare workers during the COVID-19 pandemic in the UK. *BMJ Open* 2020;10(11):e040503. doi: 10.1136/bmjopen-2020-040503 [published Online First: 20201105]
77. UK Health Security Agency (confidential internal document). Supporting COVID-19 testing and vaccination in a deprived local authority - A case study of Blackpool 2021.
78. UK Health Security Agency (confidential internal document). COVID-19 Test and Trace Contacts Insights Study, Wave 3: 1–10 April 2021, 2021.
79. UK Health Security Agency (confidential internal document). London Borough of Havering Isolation Outreach Pilot Evaluation nd.
80. Jayes L, Bogdanovica I, Johnston E, et al. Perspectives of attenders and non-attenders to SARS-CoV-2 asymptomatic community testing in England: a qualitative interview study. *BMJ Open* 2022;12(9):e064542. doi: 10.1136/bmjopen-2022-064542 [published Online First: 20220922]
81. UK Health Security Agency (confidential internal document). Evaluation of COVID-19 guidance and support for the migrant worker community delivered by voluntary and community sector organisations (VCSOs), 2022.
82. Dennis A, Robin C, Carter H. The social media response to twice-weekly mass asymptomatic testing in England. *BMC Public Health* 2022;22(1):182. doi: 10.1186/s12889-022-12605-2 [published Online First: 20220127]
83. UK Health Security Agency (confidential internal document). Adult Social Care Vaccination & COVID-19 Testing Report, 2022.
84. Smith LE, Potts HWW, Amlôt R, et al. Adherence to the test, trace, and isolate system in the UK: results from 37 nationally representative surveys. *BMJ* 2021;372:n608. doi: 10.1136/bmj.n608 [published Online First: 2021/04/02]
85. UK Health Security Agency (confidential internal document). Baselining the asymptomatic staff testing journey in adult social care (ASC).
86. UK Health Security Agency (confidential internal document). London Borough of Havering Isolation Outreach Pilot Evaluation.
87. Mahmood F, Acharya D, Kumar K, et al. Impact of COVID-19 pandemic on ethnic minority communities: a qualitative study on the perspectives of ethnic minority community leaders. *BMJ Open* 2021;11(10):e050584. doi: 10.1136/bmjopen-2021-050584 [published Online First: 20211025]

For peer review only - http://bmjopen.bmj.com/site/about/guidelines.xhtml

88. UK Health Security Agency (confidential internal document). COVID-19 Test and Trace Cases Insights Study, nd.
89. Hills S, Eraso Y. Factors associated with non-adherence to social distancing rules during the COVID-19 pandemic: a logistic regression analysis. *BMC Public Health* 2021;21(1):352. doi: 10.1186/s12889-021-10379-7 [published Online First: 20210213]
90. Wright L, Paul E, Steptoe A, et al. Facilitators and barriers to compliance with COVID-19 guidelines: a structural topic modelling analysis of free-text data from 17,500 UK adults. *BMC Public Health* 2022;22(1):34. doi: 10.1186/s12889-021-12372-6 [published Online First: 20220106]
91. Burton A, McKinlay A, Dawes J, et al. Understanding Barriers and Facilitators to Compliance with UK Social Distancing Guidelines During the COVID-19 Pandemic: A Qualitative Interview Study. *Behaviour Change* 2022;40(1):30-50. doi: 10.1017/bec.2021.27
92. Wu JS, Font X, McCamley C. COVID-19 social distancing compliance mechanisms: UK evidence. *Environ Res* 2022;205:112528. doi: 10.1016/j.envres.2021.112528 [published Online First: 20211223]
93. Freeman D, Loe BS, Chadwick A, et al. COVID-19 vaccine hesitancy in the UK: the Oxford coronavirus explanations, attitudes, and narratives survey (Oceans) II. *Psychol Med* 2022;52(14):3127-41. doi: 10.1017/S0033291720005188 [published Online First: 20201211]
94. Radic A, Koo B, Gil-Cordero E, et al. Intention to Take COVID-19 Vaccine as a Precondition for International Travel: Application of Extended Norm-Activation Model. *Int J Environ Res Public Health* 2021;18(6) doi: 10.3390/ijerph18063104 [published Online First: 20210317]
95. Coroiu A, Moran C, Campbell T, et al. Barriers and facilitators of adherence to social distancing recommendations during COVID-19 among a large international sample of adults. *PLoS One* 2020;15(10):e0239795. doi: 10.1371/journal.pone.0239795 [published Online First: 20201007]
96. Ashworth M, Thunstrom L, Cherry TL, et al. Emphasize personal health benefits to boost COVID-19 vaccination rates. *Proc Natl Acad Sci U S A* 2021;118(32) doi: 10.1073/pnas.2108225118
97. Schwartz SH. Normative Influences on Altruism. Advances in Experimental Social Psychology Volume 101977:221-79.
98. UK Health Security Agency (confidential internal document). COVID-19 Test and Trace Contacts Behavioural Insights Study.
99. Brooks SK, Webster RK, Smith LE, et al. The psychological impact of quarantine and how to reduce it: rapid review of the evidence. *Lancet* 2020;395(10227):912-20. doi: 10.1016/S0140-6736(20)30460-8 [published Online First: 20200226]
100. UK Health Security Agency (confidential internal document). Compliance with self-isolation for those within NHS Test and Trace: Summary of the Evidence, 2021.
101. Her Majesty's Government. Social Distancing Review: Report. London, united Kingdom: Her Majesty's Government,, 2021:1-32.
102. Husted M, Gibbons A, Cheung WY, et al. COVID-19 vaccination hesitancy in adults in the United Kingdom: Barriers and facilitators to uptake. *Health Psychol* 2023;42(8):584-92. doi: 10.1037/hea0001256 [published Online First: 20230112]
103. Mounier-Jack S, Paterson P, Bell S, et al. Covid-19 vaccine roll-out in England: A qualitative evaluation. *PLoS One* 2023;18(6):e0286529. doi: 10.1371/journal.pone.0286529 [published Online First: 20230602]
104. Abba-Aji M, Stuckler D, Galea S, et al. Ethnic/racial minorities' and migrants' access to COVID-19 vaccines: A systematic review of barriers and facilitators. *J Migr Health* 2022;5:100086. doi: 10.1016/j.jmh.2022.100086 [published Online First: 20220218]
105. Leigh JP, Moss SJ, White TM, et al. Factors affecting COVID-19 vaccine hesitancy among healthcare providers in 23 countries. *Vaccine* 2022;40(31):4081-89. doi: 10.1016/j.vaccine.2022.04.097 [published Online First: 20220505]

For peer review only - http://bmjopen.bmj.com/site/about/guidelines.xhtml

106. Matthias MS, Salyers MP, Frankel RM. Re-thinking shared decision-making: context matters. *Patient Educ Couns* 2013;91(2):176-9. doi: 10.1016/j.pec.2013.01.006 [published Online First: 20130211]

For peer review only

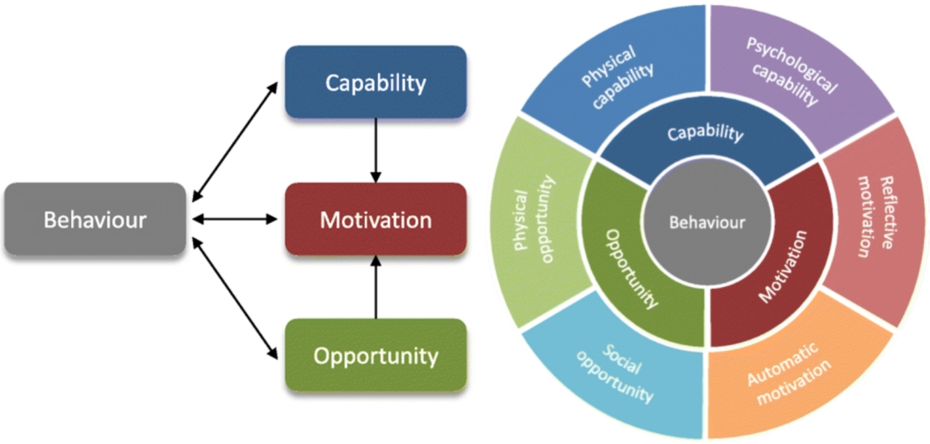

Figure 1. The dimensions of the Capability, Opportunity, and Motivation Model of Behaviour (COM-B), adapted from McDonagh et al's application to a systematic review of chlamydia testing facilitators.17

326x154mm (130 x 130 DPI)

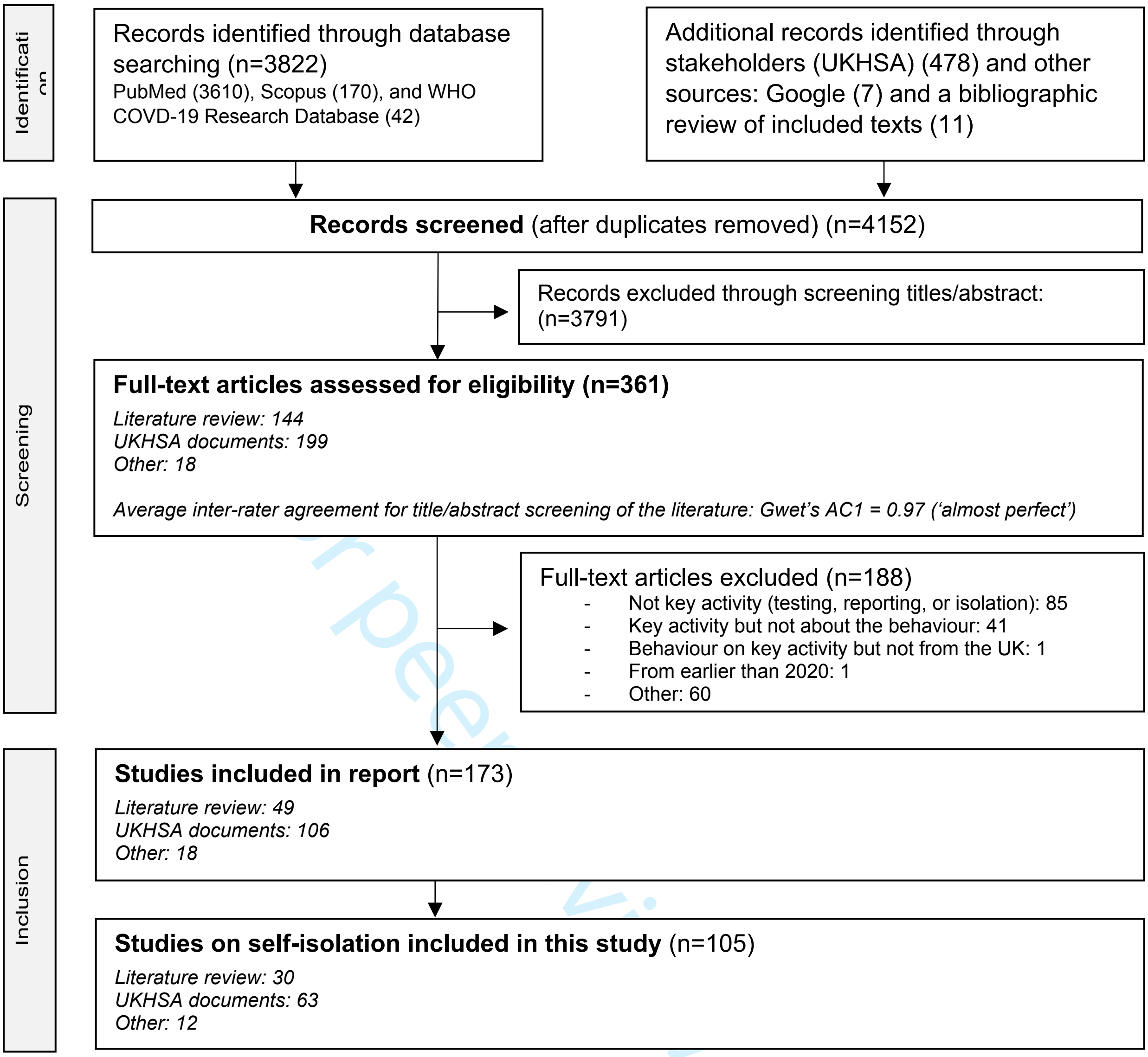

Figure 1. Preferred Reporting Items for Systematic Reviews and Meta-analyses extension for scoping review (PRISMA-ScR) flow diagram outlining the search and inclusion of the literature.

For peer review only

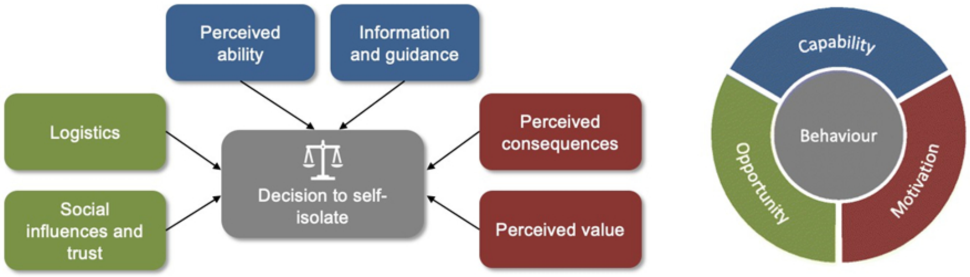

Figure 3. Overview of the themes describing the influences on people’s decision to comply with isolation guidance.

402x137mm (57 x 57 DPI)

Decision to self-isolate during the COVID-19 pandemic in the UK: a rapid scoping study

Supplemental tables and figures

Supplemental Table 1. Search categories and examples of search terms. Search strings were adapted to each database. 2
Supplemental Table 2. Justifications and rationale for databases searched 3
Supplemental Table 3. Data extraction template 4
Supplemental Table 4. Included sources in the study examining self-isolation behaviour during the COVID-19 pandemic in the UK 5
Supplemental Table 5. Definitions of the stages of the pandemic....35

Supplemental Figure 1. Infographic to define the stages of the pandemic, with details 36

For peer review only

For peer review only - http://bmjopen.bmj.com/site/about/guidelines.xhtml

Supplemental Table 1. Search categories and examples of search terms. Search strings were adapted to each database.

Category	Search terms
**COVID-19**	COVID* OR corona OR coronavirus OR SARS-CoV-2 OR "SARS CoV 2" OR "SARS CoV-2" OR SARS-CoV2 OR SARSCoV2 OR "Severe Acute Respiratory Syndrome Coronavirus 2" OR "Severe Acute Respiratory Syndrome Corona Virus 2" OR 2019-nCoV
	**AND**
**Key activities**	test* OR screen* OR RT-PCR OR PCR OR “polymerase chain reaction” OR “lateral flow” OR “lateral flow device*” OR “lateral flow assay*” OR LFD OR self-test* OR “test and trace” OR “contact trac*” OR surveillance OR POCT OR report* OR self-report* OR selfreport* OR “test positive” OR “testing positive” OR result* OR “self-isolation” OR “self isolation” OR isolat* OR containment OR reopening OR re-opening OR mitigat* OR flatten*
	**AND**
**Behaviour, barriers, and facilitators**	knowledge OR understand* OR attitude* OR perception* OR perceive OR belief* OR believ* OR expectation* OR trust OR willing* OR intention* OR behaviour* OR behavior* OR practice* OR enact* OR engag* OR adher* OR complian* OR comply OR experience* OR view* OR motivation* OR barrier* OR block* OR challeng* OR difficult* OR facilitat* OR enabl* OR access* OR feasib* OR accept* OR uptake
	**AND**
**Research methods**	qualitative* OR interview* OR FGD OR "focus group*" OR survey* OR questionnair* OR mixed-method* OR “mixed method*” OR ethnograph* OR theme OR thematic* OR “grounded theory” OR “content analysis” OR field-work OR “field work” OR selfreport* OR self-report* OR “self report*” OR view* OR experience* OR hermeneutic OR phenomenolog*
	**AND**
**Geographic setting**	“United Kingdom” OR UK OR England OR Ireland OR Irish OR Scot* OR Wales OR Britain OR British OR NHS OR “National Health Service*” OR UKHSA OR “United Kingdom Health Security Agency” OR “UK Health Security Agency” OR “Channel Island*” OR London OR Birmingham OR Liverpool OR Manchester OR Cardiff OR Belfast OR Edinburgh OR Glasgow

For peer review only - http://bmjopen.bmj.com/site/about/guidelines.xhtml

Supplemental Table 2. Justifications and rationale for databases searched

Database	Rational for inclusion in the search strategy
**PubMed**	As the primary database for biomedical and health behaviour literature, PubMed not only covers Medline but also offers a vast range of other relevant sources. This includes in-process citations, out-of-scope citations, ahead of print citations, and author manuscripts of NIH funded research, making it a pivotal source for comprehensive and timely information.[1]
**Scopus**	Scopus provides interdisciplinary coverage, encompassing nearly 100 million biomedical, physical, life, and social sciences research records from over 7000 publishers. Its extensive citation analysis capabilities also help to understand the breadth and depth of literature influence. Additionally, the database is curated by independent subject matter experts, recognised as leaders in their fields, adding to the credibility for information for this study.[2]
**The World Health Organization COVID-19 Research Database**	The literature cited in the WHO COVID-19 Research Database was updated daily (Tuesday through Saturday) during the pandemic from searches of bibliographic databases, hand searching, and the addition of other expert-referred scientific articles. This database represents a comprehensive multilingual source of current literature on the topic, with the backing and expertise of a global health authority. While it may not be exhaustive, new research was added regularly. Databases searched include MEDLINE, Scopus, EuropePMC, Web of Science, ProQuest Central, EMBASE, medRxiv, ICTRP, WHO COVID, ScienceDirect, and grey literature.[3]
**Google Scholar**	Google Scholar covers a broad range of literature types, including theses, conference proceedings, and articles from academic publishers. Its inclusion can identify research overlooked in more specialized databases, ensuring comprehensive coverage and filling obvious gaps.
**Review of bibliographies of included studies**	A classic snowballing method, reviewing bibliographies can unveil seminal and influential works that may not be captured by traditional database searches. This method ensures inclusion of studies deemed foundational or highly relevant by scholars in the field and identifies evidence deemed relevant by those sources included in the initial screening process.
**Stakeholder consultation**	A strength of scoping studies is the explicit involvement of stakeholders in not only framing the research question, sense-checking the results, and informing the interpretation but also in identifying critical sources of evidence that may otherwise have been missed.[4]<br>In this instance, the UK Health Security Agency (UKHSA) was consulted as a major stakeholder to identify research commissioned by UKHSA.

For peer review only - http://bmjopen.bmj.com/site/about/guidelines.xhtml

Supplemental Table 3. Data extraction template

Manuscript information																					Key messages	Notes	Bibliographic review	Operational insights
Publication											Methodology						Setting							
No.	Rayyan ID	Authors	Title	**Inclusion**	**Reason for exclusion**	**EXTRACTOR** ***[Name]***	Publication date *[year]*	Journal or Source	**Publication stage**	Notes	**Methods**	Participants *[Who were the interviews/ surveys with? Whose views and experiences are represented?]*	Number of participants	**Data collection period** ***NB!***	Obvious limitations *[copy and paste limitations from the limitations section]*	Notes *[e.g. if 'other' chosen, expand here]*	**Country**	**Scope**	**Site/ Setting**	Notes	***Summary of the findings*** *- bullet points - big picture overview of the key points for OUR question (not the aims of the paper) - add own summary of the key points (paraphrase and summarise) and can copy and paste the conclusions if they summarise the key findings we need*	*Anything that doesn't fit in the categories, explanations etc* *<As well as your thoughts and notes and interpretations>*		
1																								
2																								
3																								
4																								
5																								

	Isolation Behaviour - copy and paste results and quotes																							
	Isolation								Person														Other	
	Perceived attributes of the isolation itself						Trust		Knowledge				Individual and context											
	**Perceived PHYSICAL factors of isolation itself** *[e.g. physical burden of the isolation itself like (physical) isolation fatigue, discomforts of staying in isolation]*		**Perceived MENTAL factors of isolation itself** *[e.g. like anxiety around isolation, isolation fatigue, "dread" of isolation (again)]*		**Perceived RESOURCE factors of isolation itself** *[e.g. resource factors like financial costs to the user of the isolation process, time etc]*		**Trust in the process of isolation and system** *[e.g. credibility and trust in the institutions delivering/providing the isolation or guidance on isolation)*		**Understanding of isolation requirements** *[i.e. does the individual understand if and when they SHOULD isolate? e.g. when, which type of isolation, where to isolate and for how long, including for what symptom]*		**Perceived capability to isolate** *[i.e. does the individual understand HOW to isolate? e.g. understanding of how to do it (in theory) and feeling able to take the steps to isolate]*		**Logistics of isolation** *[i.e. ACCESS to isolation e.g. convenience, ease of access, ease of isolating, isolation resources, ability to isolate within their home and separate from other family members (separate rooms, space etc)]*		**Personal factors influencing isolation** *[e.g. mental health, burnout, resilience, perceptions of risk of contracting COVID, perceptions of risk of consequences if contract COVID etc]*		**Perception of value in isolation** *[e.g. to oneself, to others, to society, as a duty etc, perceived responsibility towards others to isolate, perceptions of COVID threat level, perceptions of necessity to isolate]*		**Perceived consequences of isolation** *[e.g. e.g. not being to work, or having sick leave or having to look after others, impact on finances, education, work, career, caring responsibilities etc]*		**Social influences related to isolation** *[e.g. stigma in taking part/not taking part, perceived freedom to say no/yes to isolation)*		**Other** *[not captured by other categories]*	
	Barriers	Enablers	Barriers	Enablers	Barriers	Enablers	Barriers	Enablers	Barriers	Enablers	Barriers	Enablers	Barriers	Enablers	Barriers	Enablers	Barriers	Enablers	Barriers	Enablers	Barriers	Enablers	Barriers	Enablers
1																								
2																								
3																								
4																								
5																								

For peer review only - http://bmjopen.bmj.com/site/about/guidelines.xhtml

Supplemental Table 4. Included sources in the study examining self-isolation behaviour during the COVID-19 pandemic in the UK

No	Publication	Methodology			Setting			
	Reference	Methods	Description of the sample	Data collection period	Pandemic stage (early, mid, late)	Country	Scope	Context
**General**								
**Literature search**								
1	Al-Oraibi A, Fothergill L, Yildirim M, Knight H, Carlisle S, O'Connor M, Briggs L, Morling JR, Corner J, Ball JK, Denning C, Vedhara K, Blake H. Exploring the Psychological Impacts of COVID-19 Social Restrictions on International University Students: A Qualitative Study. Int J Environ Res Public Health. 2022;19(13).	Interview and focus group	A total of eight online focus groups were conducted, comprising 29 international students, with group sizes varying between three to five students. Seventeen one-to-one interviews were held with university staff members.<br>International students - focus groups<br>University staff - interviews	January - February 2021	Mid pandemic	International (including UK)	Isolation	Testing in universities - Focus groups held with international university students, and interviews held with university staff.
2	Blake H, Carlisle S, Fothergill L, Hassard J, Favier A, Corner J, Ball JK, Denning C. Mixed-methods process evaluation of a residence-based SARS-CoV-2 testing participation pilot on a UK university campus during the COVID-19 pandemic. BMC Public Health. 2022;22(1):1470.	Survey, interview, and focus group	University students living onsite<br>Student survey - 152<br>Staff interviews - 13<br>Student focus groups - 30	April - June 2021	Mid pandemic	England	Testing and isolation	Convergent parallel mixed methods evaluating intervention fidelity and barriers/enablers to implementation of an asymptomatic testing programme in university.
3	Blake H, Knight H, Jia R, Corner J, Morling JR, Denning C, Ball JK, Bolton K, Figueredo G, Morris DE, Tighe P, Villalon AM, Ayling K, Vedhara K. Students' Views towards Sars-Cov-2 Mass Asymptomatic Testing, Social Distancing and Self-Isolation in a University Setting during the COVID-19 Pandemic: A Qualitative Study. Int J Environ Res Public Health. 2021;18(8).	Focus groups	25 university students from 1 institution	October 2020	Mid pandemic	England	Testing and isolation	Focus group discussion with university students on testing and isolation during the pandemic. Thematic analysis conducted on output of focus groups.

For peer review only - http://bmjopen.bmj.com/site/about/guidelines.xhtml

4	Burns J, Mc Goldrick N, Sigerson D, Edwards M, Culshaw S, Clark C, Watling C, Braid R, O'Keefe E, Gorman M, Conway DI. A Health Inequalities Impact Assessment of the surveillance of COVID-19 in asymptomatic patients attending dental settings in Scotland. Community Dent Health. 2022;39(4):254-9.	Survey	35 dental team members from across Scotland participated in the consultation.	October 2020	Mid pandemic	Scotland	Testing and isolation	A Health Inequalities Impact Assessment (HIIA) was conducted to understand the differential impacts the programme would have on the population and to improve the accessibility of the programme
5	Denford S, Martin AF, Love N, Ready D, Oliver I, Amlot R, Yardley L, Rubin GJ. Engagement With Daily Testing Instead of Self-Isolating in Contacts of Confirmed Cases of SARS-CoV-2: A Qualitative Analysis. Front Public Health. 2021;9:714041. Epub 20210803. doi: 10.3389/fpubh.2021.714041.	Interviews	52 participants, with 35 who had taken part in a feasibility study to evaluate daily contact testing, and 17 who had declined the offer and opted to self-isolate	11 - 23 December 2020 and 4 - 12 January 2021	Mid pandemic	UK	Testing and isolation	Daily contact testing rather than asymptomatic testing – focus on attitudes towards contact testing as an alternative to contacts of positive cases having to isolate. Study ran before Universal Testing roll out.
6	Denford S, Martin AF, Towler L, Mowbray F, Essery R, Bloomer R, Ready D, Love N, Amlot R, Oliver I, Rubin GJ, Yardley L. A qualitative process analysis of daily contact testing as an alternative to self-isolation following close contact with a confirmed carrier of SARS-CoV-2. BMC Public Health. 2022;22(1):1373.	Interviews	60 - People who had been in close contact with a confirmed SARS-CoV-2 carrier and had consented to take part in the trial.	24 June - 8 July 2021	Mid pandemic	England	Testing & Isolation	Daily contact testing rather than asymptomatic testing – focus on attitudes towards contact testing as an alternative to contacts of positive cases having to isolate. Study covers before and after introduction of Universal Testing.
7	Dennis A, Robin C, Carter H. The social media response to twice-weekly mass asymptomatic	Other – review of	5783 comments – members of public	5 April - 28 May 2021	Mid pandemic	England	Testing & Isolation	Analysis of social media comments

For peer review only - http://bmjopen.bmj.com/site/about/guidelines.xhtml

	testing in England. BMC Public Health. 2022;22(1):182. Epub 20220127. doi: 10.1186/s12889-022-12605-2.	social media						from members of the public in England about twice-weekly asymptomatic testing. Study is from period when Universal Testing was first introduced.
8	Eraso Y, Hills S. Self-Isolation and Quarantine during the UK's First Wave of COVID-19. A Mixed-Methods Study of Non-Adherence. Int J Environ Res Public Health. 2021;18(13). Epub 20210630. doi: 10.3390/ijerph18137015.	Survey, interview, and focus group	residents in six North London boroughs<br>681 - Survey<br>16 – interviews	1 - 31 May 2020	Mid pandemic	England	Isolation	Study conducted before Universal Testing was available
9	Hanley S, Raybould G, Baxter E, Gray J, Sharkey D, Walker KF. Maternity services' responses to the COVID-19 pandemic: how Public Health England guidance was implemented in practice. Journal of Hospital Infection. 2022;129:214-8.	Surveys	44 maternity healthcare workers from 33 NHS hospitals in England, including 14 midwives, 3 senior midwives, 2 senior trainee obstetricians, 21 consultant obstetricians, 1 clinical fellow, 1 service director, 1 labour ward coordinator and 1 clinical midwifery manager	November 2020 - July 2021	Mid pandemic	England	Testing and isolation	Patient participation in testing programme.
10	Isherwood KR, Kyle RG, Gray BJ, Davies AR. Challenges to self-isolation among contacts of cases of COVID-19: a national telephone survey in Wales. J Public Health (Oxf). 2022.	Surveys	2027 - Individuals were eligible for inclusion if they: (i) had been successfully contacted by TTP after forward contact tracing (ii) were a close contact of a confirmed case of COVID-19; (iii) were aged 18 years or over; (iv) resident in Wales: and (v) had completed their self-isolation period at the time of telephone survey. Contacts were excluded from the study if they were: (i) under the age of 18; (ii) currently self-isolating; (iii) not a resident in Wales	11 November - 1 December 2020	Mid pandemic	Wales	Isolation	Daily contact testing rather than asymptomatic testing – focus on attitudes towards contact testing as an alternative to contacts of positive cases having to isolate. Before Universal Testing
11	Jayes L, Bogdanovica I, Johnston E, Chattopadhyay K, Morling JR, Devine S, Richmond N, Langley T. Perspectives of attenders and non-attenders to SARS-CoV-2 asymptomatic community testing in England: a qualitative interview study. BMJ Open. 2022;12(9):e064542.	Interviews	With 18 members of the public who attended a community testing centre and 15 who had not	February 2021 - May 2021	Mid pandemic	England	Isolation	Community asymptomatic testing. Study period covers before and after the start of introduction of

For peer review only - http://bmjopen.bmj.com/site/about/guidelines.xhtml

								Universal Testing.
12	Knight H, Carlisle S, O'Connor M, Briggs L, Fothergill L, Al-Oraibi A, Yildirim M, Morling JR, Corner J, Ball J, Denning C, Vedhara K, Blake H. Impacts of the COVID-19 Pandemic and Self-Isolation on Students and Staff in Higher Education: A Qualitative Study. Int J Environ Res Public Health. 2021;18(20). Epub 20211012. doi: 10.3390/ijerph182010675.	Interview and focus groups	26 university staff interviews and 11 university students participating in focus groups	January 2021 - March 2021	Mid pandemic	England	Testing and isolation	University testing programme. Before the introduction of Universal Testing.
13	Mahmood F, Acharya D, Kumar K, Paudyal V. Impact of COVID-19 pandemic on ethnic minority communities: a qualitative study on the perspectives of ethnic minority community leaders. BMJ Open. 2021;11(10):e050584.	Interviews	19 - Community leaders recruited through organisations representing ethnic minority communities and religious places of worship	October - November 2020	Mid pandemic	England	Testing, reporting and isolation	Community testing focussing on ethnic minority communities. Before Universal Testing.
14	Martin AF, Denford S, Love N, Ready D, Oliver I, Amlot R, Rubin GJ, Yardley L. Engagement with daily testing instead of self-isolating in contacts of confirmed cases of SARS-CoV-2. BMC Public Health. 2021;21(1):1067.	Surveys	319 people who had agreed to daily testing 205 who were not offered daily testing - adult contacts of confirmed COVID-19 cases who consented to daily testing, and a comparison group of contacts who were not offered testing and instead self-isolated	11 – 23 December 2020 and 4 - 12 January 2021	Mid pandemic	England	Testing and isolation	Daily contact testing rather than asymptomatic testing – focus on attitudes towards contact testing as an alternative to contacts of positive cases having to isolate. Before Universal Testing.
15	Mowbray F, Woodland L, Smith LE, Amlot R, Rubin GJ. Is My Cough a Cold or Covid? A Qualitative Study of COVID-19 Symptom Recognition and Attitudes Toward Testing in the UK. Front Public Health. 2021;9:716421.	Interviews	40 people (21 members of the general population, 19 students)	30 November - 11 December 2020	Mid pandemic	England	Testing	Focus on symptom recognition to test with a mixture of general public and university students. Before Universal Testing.
16	Robin C, Symons C, Carter H. Local Community Response to Mass Asymptomatic COVID-19 Testing in Liverpool, England: Social Media Analysis. JMIR Form Res. 2022;6(8):e34422.	Other – review of media and social media comments	Overall, 1096 comments were sampled: 219 newspapers comments, 472 Facebook comments, and 405 tweets. - Members of Liverpool local community	2 November 2020 - 8 November 2020	Mid pandemic	England	Isolation	Social media analysis of responses and views towards community asymptomatic testing. Before Universal Testing.

For peer review only - http://bmjopen.bmj.com/site/about/guidelines.xhtml

17	Smith LE, Amlot R, Lambert H, Oliver I, Robin C, Yardley L, Rubin GJ. Factors associated with adherence to self-isolation and lockdown measures in the UK: a cross-sectional survey. Public Health. 2020;187:41-52.	Surveys	Adults in the UK recruited online - 217 with experience of isolation of 2240 total respondents	6 - 7 May 2020	Early pandemic	UK	Isolation	Focus on adherence to isolation rather than on testing. Relatively early in the pandemic – first lockdown. Before Universal Testing.
18	Smith LE, Potts HWW, Amlot R, Fear NT, Michie S, Rubin GJ. Intention to adhere to test, trace, and isolate during the COVID-19 pandemic (the COVID-19 Rapid Survey of Adherence to Interventions and Responses study). Br J Health Psychol. 2022;27(3):1100-18.	Surveys	12,976 - Selected only participants who lived in England due to differing restrictions across the four UK nations. and were eligible for the study if they were aged 16 years or over and lived in the United Kingdom. Quotas were applied based on age and gender (combined), and reflected targets based on data from the Office for National Statistics	27 April 2020 – 27 January 2021	Early- Mid pandemic	UK	Testing & Isolation	Before Universal Testing.
19	Smith LE, Potts HWW, Amlôt R, Fear NT, Michie S, Rubin GJ. Adherence to the test, trace, and isolate system in the UK: results from 37 nationally representative surveys. BMJ. 2021;372:n608.	Surveys	74 699 responses from 45 957 people living in the UK, aged 16 years or older	2 March 2020 to 27 January 2021	Early- Mid pandemic	UK	Testing, Reporting and Isolation	Levels of adherence to test, trace, and isolate are low, although some improvement has occurred over time. Practical support and financial reimbursement are likely to improve adherence. Targeting messaging and policies to men, younger age groups, and key workers might also be necessary.
20	Street A, Lee SJ, Bevan I. The hidden burden of medical testing: public views and experiences of COVID-19 testing as a social and ethical process. BMC Public Health. 2022;22(1):1837.	Interviews	70 - members of the general public (aged 19–85) living in the Lothian region of Scotland	7 July - 24 September 2020	Early- Mid pandemic	Scotland	Isolation	Before Universal Testing.

For peer review only - http://bmjopen.bmj.com/site/about/guidelines.xhtml

21	van der Scheer JW, Ansari A, McLaughlin M, Cox C, Liddell K, Burt J, George J, Kenny R, Cousens R, Leach B, McGowan J, Morley K, Willars J, Dixon-Woods M. Guiding organisational decision-making about COVID-19 asymptomatic testing in workplaces: mixed-method study to inform an ethical framework. BMC Public Health. 2022;22(1):1747.	Surveys and interviews	50 survey participants and 11 interviews with staff members from workplaces used as case studies	November 2020 - December 2020	Mid pandemic	England	Testing and isolation	Exploring how workers feel about workplace testing and the implications of testing and isolation on their work. Before Universal Testing.
**Google/ Bibliographic search**								
1	Atchison C, Bowman LR, Vrinten C, Redd R, Pristerà P, Eaton J, Ward H. Early perceptions and behavioural responses during the COVID-19 pandemic: a cross-sectional survey of UK adults. BMJ Open. 2021;11(1):e043577.	Surveys	Nationally representative sample of 2108 UK adults	17 March 2020 - 18 March 2020	Early pandemic	UK	Isolation	Study conducted within 48 hours of UK government advising public to stop non-essential contact with others and all unnecessary travel
2	Bevan I, Stage Baxter M, Stagg HR, Street A. Knowledge, Attitudes, and Behavior Related to COVID-19 Testing: A Rapid Scoping Review. Diagnostics (Basel). 2021;11(9).	Survey, interview, and focus groups	UK population (amongst other international samples)	March 2020 - January 2021	Early- Mid pandemic	UK	Testing and isolation	Rapid scoping review found existing research at time of review was limited in depth and scope.
3	Denford S, Towler L, Ali B, Treneman-Evans G, Bloomer R, Peto TE, Young BC, Yardley L. Feasibility and acceptability of daily testing at school as an alternative to self-isolation following close contact with a confirmed case of COVID-19: a qualitative analysis. BMC Public Health. 2022;22(1):742.	Interviews	Staff, students, and parents of students (n=63)	Not stated	Mid pandemic?	England	Testing, reporting and isolation	At time of study, close contacts of the case required to self-isolate at home for 10 days.
4	French C, Denford S, Brooks-Pollock E, Wheling H, Hickman M. Low uptake of COVID-19 lateral flow testing among university students: a mixed methods evaluation. Public Health. 2022; 204:54–62.	Surveys, interviews, and focus groups	University students. A total of 436 students completed the online survey, and 20 in-depth interviews were conducted	November - December 2020	Mid pandemic	England	Isolation	Based at one university assessing LFT testing uptake and the differences between demographic variations
5	Kyle RG, Isherwood KR, Bailey JW, Davies AR. Self-isolation confidence, adherence and challenges: Behavioural insights from contacts of cases of COVID-19 starting and completing	Survey, interviews, and focus group	General population with 13,531 responses from survey and 1011 telephone surveys	November 2020 - January 2021	Mid pandemic	Wales	Isolation	Report published by NHS Public Health Wales exploring factors associated to

For peer review only - http://bmjopen.bmj.com/site/about/guidelines.xhtml

	self-isolation in Wales. Cardiff, Wales: Iechyd Cyhoeddus Cymru/Public Health Wales, 2021.							adherence to isolation with the aim to inform content of public health messaging and support.
6	Lin L, Song Y, Wang Q, Pu J, Sun FY, Zhang Y, Zhou X, Larson HJ, Hou Z. Public Attitudes and Factors of COVID-19 Testing Hesitancy in the United Kingdom and China: Comparative Infodemiology Study. JMIR Infodemiology 2021;1(1): e26895	Other-Social listening	General population - 3856 UK tweets and 9299 Chinese Sina Weibo posts	1 June - 15 July 2020	Early pandemic	International (including UK)	Testing, reporting and isolation	Comparative analysis between UK and China to assess public attitudes and uptake regarding COVID-19 testing.
7	Love N, Ready D, Turner C, Yardley L, Rubin J, Hopkins S, et al. The acceptability of testing contacts of confirmed COVID-19 cases using serial, self-administered lateral flow devices as an alternative to self-isolation. medRxiv. 2021.	Interviews	General population (n=882)	11 - 23 December 2020 And 4 - 12 January 2021	Mid pandemic	England	Testing, reporting & isolation	Investigating a 'test to enable' approach as part of England's tracing strategy dur to suboptimal adherence to self-isolation guidance.
8	Marshall GC, Skeva R, Jay C, Silva MEP, Fyles M, House T, Davis EL, Pi L, Medley GF, Quilty BJ, Dyson L, Yardley L, Fearon E. Public perceptions and interactions with UK COVID-19 Test, Trace and Isolate policies, and implications for pandemic infectious disease modelling. [version 1; peer review: awaiting peer review] F1000Research. 2022;11:1005.	Interviews	20 adults over the age of 18 living in the UK	Summer 2021	Mid pandemic	UK	Testing, reporting and isolation	Interviews took place during a policy change (19 July 2021), so the findings may be overrepresenting aspects of the interview context. Whilst efforts were made to recruit a diversity of views, participants were necessarily those who chose to take part in this study.
9	Smith LE, West R, Potts HWW, Amlot R, Fear NT, Rubin GJ, Michie S. Knowledge of Self-Isolation Rules in the UK for Those Who Have Symptoms of COVID-19: A Repeated Cross-Sectional Survey Study. Int J Environ Res Public Health. 2023;20(3).	Surveys	78,573 responses from 51,881 participants of the general population (UK adults)	November 2020 - February 2022	Mid- Late pandemic	UK	Isolation	At time of study adherence to self-isolation in people who develop key COVID-19

For peer review only - http://bmjopen.bmj.com/site/about/guidelines.xhtml

								symptoms is low in the UK.
10	Street A, Baxter MS, Christison S, Bevan I, Bauld L. Student views and experiences of asymptomatic COVID-19 testing at the University of Edinburgh. DiaDev Investigating Diagnostics in Global Health, The University of Edinburgh, 2021.	Interviews	24 University students who had completed a 10-minute survey	November - December 2020	Mid pandemic	Scotland	Testing and isolation	Main goal at the time of the mass testing programme was to minimise transmission of COVID19 when students travelled home for winter break.
**Stakeholder identified sources (UKHSA Secretariat)**								
1	ICF Consulting Services Limited. Evaluation of the Enduring Transmission Pilot in Peterborough, Fenland and South Holland. 2022.	Interviews	Untreated - 764,686<br>Treated - 228<br>Total - 764,914 - A total of 717 treated people were included in the pilot's MI (individuals supported by Rosmini from June to December 2021). Of these, 504 individuals could be linked to CTAS data based on their postcode, gender, ethnicity, and age (measured at assessment date). Among these 504 treated people, 276 could not be used for analysis for various reasons. Removing these 276 individuals from the sample of 504 treated people linked to the CTAS database resulted in a final sample of 228 treated people.<br><br>The sample of 764,686 untreated people was obtained by considering all individuals aged 18-67 who resided in LADs other than Fenland, Peterborough, and South Holland (after excluding all LADs where other Test & Trace pilots were rolled out), and with non-missing value on all the variables of interest (demographics and outcome).	23 June - 22 November 2021	Mid pandemic	England	Isolation	Evaluation was completed in two stages, a qualitative process evaluation and an impact evaluation stage.
2	Institute for Connected Communities. Newham Council welfare check-in call pilot - Learning from a rapid evaluation 2021–2022: University of East London; 2022. Available from: https://repository.uel.ac.uk/download/6d85e6855	Interviews	41 - The research participants were adult males and females, aged 20 to 75+, from Black, Asian, and ethnic minority groups and White British, from	November - December 2021	Mid -Late pandemic	England	Isolation	'Other' settings.

For peer review only - http://bmjopen.bmj.com/site/about/guidelines.xhtml

	9f8c6330ed94896e2165009f6a7bd6bce2324522 00857a16a7ed2b9/2819325/Welfare%20Check-In%20Call%20Evaluation%20Report.pdf.		across the Borough, and UK nationals and non-nationals. Of the 1,282 who were consented to take part in the study by the Council, 571 were eligible for screening calls. The 41 interviewed participants were clustered around seven postcode areas within the Borough and represented the general demographic characteristics and proportions of Newham. All the research participants who took part in the study had tested positive for COVID-19.					
3	UK Health Security Agency (confidential internal document), Brand perceptions tracker - wave 1. 2020.	Surveys	2,029 adult - Adults aged 18+ in England	9 -17 December 2020	Mid pandemic	England	Testing & isolation	Universal Testing.
4	UK Health Security Agency (confidential internal document).Calls (not texts) work best to encourage self-isolation – results from a field experiment in the Test & Trace programme. 2020.	Other	6,812 trial participants (2652 control, 1219 texts only, 1503 calls only, 1438 texts + calls) - close contacts of confirmed case	17 August - 10 September 2020	Early- Mid pandemic	England	Isolation	Randomised controlled field trial who'd recently been in close contact with a confirmed case. At the time of study, 14-day isolation period required.
5	UK Health Security Agency (confidential internal document). Compliance with self-isolation for those within NHS Test and Trace: Summary of the Evidence. 2021.	Surveys	Exact number hasn't been stated - Surveys were conducted internally and externally as well as analysis of data collected through NHS Test and Trace. Data collection was done from the following sources:<br>1. Internal surveys<br>2. Test and trace data on contacts<br>3. ONS compliance with self-isolation surveys<br>4. Test and Trace compliance	1. Internal Survey<br>a) August - September 2020<br>b) November 2020 - March 2021<br>2) ONS	Early – Mid pandemic	England	Isolation	The issues in obtaining an accurate estimate of compliance include considering the complexities around what is and is not

For peer review only

			check-in calls data<br>5. Test and Trace compliance check-in calls - landline data	compliance surveys – February - March 2021				permitted, including changes over time; the understanding or misunderstanding of the requirements among those who are isolating and the impact this can have on their self-reports. Due to these complexities, it is useful to compare sources of data that use different approaches and methods for obtaining estimates as this can strengthen confidence in their reliability.
6	UK Health Security Agency (confidential internal document). Compliance with self-isolation: publishing findings of ONS commissioned surveys.2021.	Other	n/a - n/a	Not stated	?	England	Isolation	Summary document of the published findings of ONS commissioned surveys to understanding of how well people isolate and what drives compliance.

For peer review only - http://bmjopen.bmj.com/site/about/guidelines.xhtml

7	UK Health Security Agency (Confidential internal document), COVID-19 Test and Trace Contacts Behavioural Insights Study - Contacts not required to self-isolate; Wave 1. 2021.	Interviews	1100 adults- aged 18 years or over Respondents were sampled through the Contact Tracing and Advice (CTAS) database, held by NHS TT.	25 – 30 October 2021	Mid pandemic	England	Testing & isolation	Survey for adults who had been in contact with someone who had tested positive for COVID-19 and were not required to self-isolate due to vaccination status.
8	UK Health Security Agency (Confidential internal document), COVID-19 Test and Trace Contacts Behavioural Insights Study - Contacts not required to self-isolate; Wave 2. 2021.	Interviews	918 adults - Respondents were sampled through the Contact Tracing and Advice (CTAS) database, held by NHS TT.	22 – 27 November 2021	Mid pandemic	England	Testing & isolation	Survey for adults who had been in contact with someone who had tested positive for COVID-19 and were not required to self-isolate due to vaccination status.
9	UK Health Security Agency (Confidential internal document), COVID-19 Test and Trace Contacts Behavioural Insights Study - Contacts not required to self-isolate; Wave 3. 2022.	Interviews	1078 - Respondents were sampled through the Contact Tracing and Advice (CTAS) database, held by NHS TT.	10 - 15 January 2022	Late pandemic	England	Testing & isolation	Survey for adults who had been in contact with someone who had tested positive for COVID-19 and were not required to self-isolate due to vaccination status. Strongly advised to take daily LFD for 7 days or until 10 days since their last contact

For peer review only - http://bmjopen.bmj.com/site/about/guidelines.xhtml

								with a positive case if earlier.
10	UK Health Security Agency (Confidential internal document). COVID-19 Test and Trace Contacts Behavioural Insights Study - Contacts not required to self-isolate; Wave 5. 2022.	Interviews	1112 - Respondents were sampled through the Contact Tracing and Advice (CTAS) database, held by NHS TT.	21 - 26 February 2022	Late pandemic	England	Testing & isolation	Survey for adults who had been in contact with someone who had tested positive for COVID-19 and were not required to self-isolate due to vaccination status. Strongly advised to take daily LFD for 7 days or until 10 days since their last contact with a positive case if earlier.
11	UK Health Security Agency (confidential internal document). Daily Testing for Contacts of COVID-19 Cases (DTCC) – Evaluation. 2022.	Surveys	c.9,838 respondents - Customers'	17 December 2021 - 19 January 2022	Late pandemic	England	Testing, reporting & isolation	Interim findings from the evaluation of the Daily Testing of contacts of COVID-19 (DTCC) Question 2: To what extent are people following the guidance to test and acting appropriately on the results of the tests?
12	UK Health Security Agency (confidential internal document). Does mass testing reduce compliance with self-isolation of confirmed positive cases of SARS-CoV-2? Evidence from the Liverpool mass testing pilot. 2021.	Surveys	In total 247 participants with a Liverpool address had participated in the mass testing pilot and 277 tested positive through normal, symptomatic channels. In addition, the study included 3 participants who had participated in the mass testing pilot but did not have a Liverpool address and 678 participants who did not have a Liverpool address and tested positive through normal,	Not stated	Unclear	England	Isolation	There are concerns that participation in asymptomatic community testing may undermine adherence to self-isolation compliance among those who test positive for

For peer review only - http://bmjopen.bmj.com/site/about/guidelines.xhtml

			symptomatic channels. - All participants had tested positive for SARS-CoV-2, had their details passed to NHS Test and Trace and had supplied a telephone contact number.					SARS-CoV-2. Regression analysis and propensity score matching was used to assess the impact of participation in the Liverpool mass testing pilot on compliance.
13	UK Health Security Agency (confidential internal document). DTCC (Daily Testing of Covid Contacts) - Voice of the Customer Insight update. 2022.	Surveys	9838 - <customers at the main stages of the test, trace, and isolate journey>	17 December 2021 - 19 January 2022	Late pandemic	England	Testing & isolation	Universal Testing.
14	UK Health Security Agency (confidential internal document). DTCC – Research Findings January 2022. 2022.	Interviews	Unclear - Online focus groups and 18-24 y/o, none following COVID regulations or only following very sporadically	10 January - 17 January 2022	Late pandemic	England	Testing, reporting & isolation	Universal Testing.
15	UK Health Security Agency (confidential internal document). Effect of test and trace support payments scheme on ease of self isolation and isolation compliance- Analysis of ONS Isolation Compliance Survey Data. 2021.	Surveys	12,486 respondents - The analysis uses data from surveys of confirmed positive cases and contacts conducted by ONS and is broken down by characteristics including age, gender, ethnicity, and receipt of any benefits.	March - July 2021	Mid pandemic	England	Isolation	12th October 2020, UK government launched Test and Trace Support Payment (TTSP) scheme (£500 support payment for those needing to isolate and on low incomes).
16	UK Health Security Agency (Confidential internal document). Evaluation of COVID-19 guidance and support for the migrant worker community delivered by voluntary and community sector organisations (VCSOs). 2022.	Interviews	23 service users and 8 stakeholders - Service users (migrant workers) and stakeholders (staff from voluntary and community sector organisations)	29 March - 7 April 2022	Late pandemic	England	Testing & isolation	Previous work by the UKHSA Evaluation team found that Voluntary and community sector organisations (VCSOs) play an important role in delivering support to 'disproportionately impacted' and 'under-served' communities

For peer review only - http://bmjopen.bmj.com/site/about/guidelines.xhtml

								such as migrant workers.
17	UK Health Security Agency (confidential internal document). Exploring Compliance with Self-Isolation among Deprived Communities: A Qualitative Research Protocol. 2021.	Interviews	1104 - Deprived communities (postcodes in bottom three deciles Index of Multiple Deprivation)	15 - 19 March 2021	Mid pandemic	England	Isolation	NHS Test and Trace refreshed its business plan in December 2020, reemphasising the importance of breaking the transmission of COVID-19 and highlighting that role contact tracing and self-isolation, of those who have COVID and their close contacts, plays in achieving this aim.
18	UK Health Security Agency (confidential internal document). Impact evaluation of a temporary raise in the wage eligibility threshold for the Test & Trace Support Payment in Bradford. 2021.	Other	Individuals who started a self-isolation period and for whom a compliance outcome is observed (postintervention)= approx. 15,000; individuals who started a self-isolation period = (postintervention) approx. 83,000; individuals who were reached by Test & Trace (postintervention) = approx. 22,000. - Residents of Bradford/other LADs (cases or contacts) who started self-isolating in the pre- or post-intervention period; or those who were reached by Test & Trace via phone in the pre- or post-intervention period; or shared the names of one/more contacts in the pre- or post-intervention period. Note: Only individuals aged between 18 and 67 (working-age population) and who reside in Bradford or one among the other 303 English LADs are included in the analysis	10 April - 19 May 2021 And 1 March - 9 April 2021 (the 40 days preceding the pilot).	Mid pandemic	England	Testing, reporting & isolation	Between 10th April and 19th May 2021, the wage eligibility threshold for the TTSP was raised in Bradford from £350 to £500 (people within this income range, defined newly eligible individuals, were the main target of the intervention). It was hoped that the intervention would help increase the proportions of individuals claiming and getting TTSP.
19	UK Health Security Agency (Confidential internal document). Isolation Survey Report. 2020.	Surveys	2783 respondents (1818 cases, 965 contacts)	16 November	Early pandemic	England	Isolation	The survey displays

For peer review only - http://bmjopen.bmj.com/site/about/guidelines.xhtml

				to 7 December 2020				difference of attitudes and risk perceptions, which were measured in 5 points scale, between self-isolation compliers and non-compliers. The variation can be seen at items how easy to self-isolate, confidence in self-isolation to reduce virus spread, trust of information about Covid-19 from government, and whether government has enacted right measures to protect public. In risk perception, there was a differing perceived risk intensity of Covid-19 to society.
20	UK Health Security Agency (confidential internal document). Kingston Hospital Tracing Pilot-Interim Findings.	Other	Baseline period (March 22nd-June 17th, 2021): 759<br><br>Pilot period (from June 18th-4th November): 2364 - Hospital patients	Baseline period: 22 March -17 June 2021<br><br>Pilot period: 18 June - 4 November 2021	Mid pandemic	England	Reporting & Isolation	In February 2021, we identified that 12% of cases (163 people) that had come through to LCT in Kingston had not completed contact tracing because they were in hospital. To address this gap in contact tracing and offer

For peer review only

								local welfare support to potentially vulnerable resident cases in hospital, Kingston started the Hospital Tracing pilot in June 2021.
21	UK Health Security Agency (confidential internal document). Kirklees Self-isolation Pilot: Evaluation Report. 2021.	Mixed methods	Not clear - Inclusion criteria for the pilot were that individuals:<br><br>1) were required to self-isolate following a positive COVID-19 result; or as a close contact of a confirmed COVID-19 case;<br>2) adequately demonstrated a loss in income due to self-isolation;<br>3) earned £26,000 or less per annum;<br>4) were unable to work from home.	27 May – 22 June 2021	Mid pandemic	England	Testing & isolation	The Kirklees self-isolation pilot is aimed at improving self-isolation outcomes, among other key metrics, using a financial mechanism. The pilot trials a revised version of the Test and Trace Support Payment Scheme (TTSP) with a broader set of eligibility criteria and an income replacement policy. The pilot ran for four weeks between 27 May and 22 June 2021.
22	UK Health Security Agency (confidential internal document). Lambeth Self-isolation Pilot: Impact Evaluation.	Other	not clear - Residents of Lambeth earning up to £30,000 per year were eligible to apply for financial support during self-isolation	Not stated	Unclear	England	Testing & isolation	Universal Testing.
23	UK Health Security Agency (confidential internal document). London Borough of Havering Isolation Outreach Pilot Evaluation.	Mixed methods	not clear - The pilot only visited index cases in the community and didn’t visit close contact, those in hospital, social care provision or deceased.	7 June - 18 July 2021	Mid pandemic	England	Isolation	To support residents who were isolating due to testing positive with COVID-19, Havering Council embarked on a

For peer review only - http://bmjopen.bmj.com/site/about/guidelines.xhtml

								pilot to visit all positive cases within the community twice across the residents 10-day isolation period
24	UK Health Security Agency (confidential internal document). Office for National Statistics - COVID Test and Trace Contacts Insights Study. Wave 5. 2021.	Interviews	918 - Respondents were sampled through the Contact Tracing and Advice (CTAS) database, held by NHS TT.	4 – 8 May 2021	Mid pandemic	England	Testing & isolation	Who had been in contact with someone who has tested positive for coronavirus (COVID-19) and were at (or near) the end of their 10-day self-isolation period.
25	UK Health Security Agency (confidential internal document). Office for National Statistics - COVID Test and Trace Contacts Insights Study. Wave 6. 2021.	Interviews	968 - Respondents were sampled through the Contact Tracing and Advice (CTAS) database, held by NHS TT.	1 – 5 June 2021	Mid pandemic	England	Testing & isolation	Who had been in contact with someone who has tested positive for coronavirus (COVID-19) and were at (or near) the end of their 10-day self-isolation period.
26	UK Health Security Agency (confidential internal document). Office for National Statistics - COVID Test and Trace Contacts Insights Study. Wave 7a. 2021.	Interviews	990 - Respondents were sampled through the Contact Tracing and Advice (CTAS) database, held by NHS TT.	28 June- 3 July 2021	Mid pandemic	England	Testing & isolation	Who had been in contact with someone who has tested positive for coronavirus (COVID-19) and were at (or near) the end of their 10-day self-isolation period.
27	UK Health Security Agency (confidential internal document). Office for National Statistics – COVID Test and Trace Contacts Insights Study. Wave 7b. 2021.	Surveys	1122 - adults aged 18 or more who has tested positive for Covid-19 and were at the end of their 10-day self-isolation period.	8 - 13 March 2022	Late pandemic	England	Isolation	
28	UK Health Security Agency (confidential internal document). Office for National Statistics - COVID	Interviews	946 - Respondents were sampled through the Contact Tracing and	9 – 16 August 2021	Mid pandemic	England	Testing & isolation	Who had been in contact with someone who

For peer review only - http://bmjopen.bmj.com/site/about/guidelines.xhtml

	Test and Trace Contacts Insights Study. Wave 8. 2021.		Advice (CTAS) database, held by NHS TT.					has tested positive for coronavirus (COVID-19) and were at (or near) the end of their 10-day self-isolation period.
29	UK Health Security Agency (confidential internal document). Office for National Statistics - COVID Test and Trace Cases Insights Study: Wave 7. 2021.	Surveys	881- adults aged 18 or more who has tested positive for Covid-19 and were at the end of their 10-day self-isolation period.	27 September - 2 October 2021	Mid pandemic	England	Isolation	
30	UK Health Security Agency (confidential internal document). Office for National Statistics - COVID Test and Trace Cases Insights Study. Wave 8. 2021.	Surveys	976 - adults aged 18 or more who has tested positive for Covid-19 and were at the end of their 10-day self-isolation period.	1 - 6 November 2021	Mid pandemic	England	Isolation	Adults who had tested positive for coronavirus (COVID-19) and who were at the end of their 10-day self-isolation period.
31	UK Health Security Agency (confidential internal document). Office for National Statistics - COVID Test and Trace Self-Isolation Insights Study. Contacts required to Self-isolate. Waves 9 & 10. 2021.	Interviews	n/a - Respondents were sampled through the Contact Tracing and Advice (CTAS) database, held by NHS TT.	October - November 2021	Mid pandemic	England	Testing & isolation	COVID Test and Trace Cases Insights Study aims to understand behaviour of individuals required to self-isolate after testing positive. Universal Testing- survey aimed to exclude those who had received both doses of the vaccine or did not need to isolate.
32	UK Health Security Agency (confidential internal document). Office for National Statistics - COVID Test and Trace Contacts Insights Study. Wave 9. 2021.	Surveys	895 - adults aged 18 or more who has tested positive for Covid-19 and were at the end of their 10-day self-isolation period.	29 November - 4 December 2021	Late pandemic	England	Isolation	Understand the behaviour of individuals required to self-isolate after testing positive for coronavirus (COVID-19), as well as the

For peer review only - http://bmjopen.bmj.com/site/about/guidelines.xhtml

								effects of isolation on mental health, work, and finances.
33	UK Health Security Agency (confidential internal document). Office for National Statistics - COVID Test and Trace Cases Insights Study. Wave 11. 2022.	Surveys	1006 - adults aged 18 or more who has tested positive for Covid-19 and were at the end of their 10-day self-isolation period.	7 - 12 February 2022	Late pandemic	England	Isolation	Adults who had tested positive for coronavirus (COVID-19) and who were at the end of their self-isolation period.
34	UK Health Security Agency (confidential internal document). Office for National Statistics - COVID Test and Trace Self-Isolation Insights Study. Contacts required to Self-isolate. Wave 11. 2022.	Interviews	1184 - Respondents were sampled through the Contact Tracing and Advice (CTAS) database, held by NHS TT.	1 December 2021	Late pandemic	England	Testing & isolation	Survey for adults who were required to self-isolate after being in contact with someone who tested positive for COVID-19 and were at (or near) the end of their 10-day self-isolation period. This survey aimed to exclude those who had received both doses of the vaccine or did not need to self-isolate for another reason.
35	UK Health Security Agency (confidential internal document).Office for National Statistics - Indicators of Clinically Extremely Vulnerable Individuals' Responses to COVID-19 Outbreak. COVID High Risk Group Insights. 2021.	Interviews	2979 - Clinically extremely vulnerable populations	18 - 30 January 2021	Mid pandemic	England	Testing & isolation	COVID High Risk Group Insights survey was compiled rapidly in response to policy questions on whether the population who had been advised to shield were following shielding guidance and other information.

For peer review only

36	UK Health Security Agency (confidential internal document). Office for National Statistics - International Arrivals Insights Study: Waves 4 & 5. 2021.	Interviews	1067 - The sample included adults who arrived in the UK on 8 and 11 October (Wave 4) and 5 and 8 (Wave 5).	October - November 2021	Mid pandemic	England	Testing & isolation	Adults who had arrived in the UK from countries or territories that were not on the red list and reported that they were quarantining at an address in England.
37	UK Health Security Agency (confidential internal document). Office for National Statistics - Test and Trace Cases Insights Study. Wave 1: Pilot. 2021.	Surveys	2552 - adults aged 18 or more who has tested positive for Covid-19 and were at the end of their 10-day self-isolation period.	1 - 13 February 2022	Late pandemic	England	Isolation	COVID Test and Trace Cases Insights Study aims to understand behaviour of individuals required to self-isolate after testing positive. Universal Testing.
38	UK Health Security Agency (confidential internal document). Office for National Statistics -Test and Trace Cases Insights Study. Wave 3. 2021.	Surveys	1168 - adults aged 18 or more who has tested positive for Covid-19 and were at the end of their 10-day self-isolation period.	12 - 16 April 2021	Mid pandemic	England	Isolation	
39	UK Health Security Agency (confidential internal document). Office for National Statistics- Test and Trace Cases Insights Study. Wave 4. 2021.	Surveys	1044 - adults aged 18 or more who has tested positive for Covid-19 and were at the end of their 10-day self-isolation period.	10 -15 May 2021	Mid pandemic	England	Isolation	
40	UK Health Security Agency (confidential internal document). Office for National Statistics- Test and Trace Cases Insights Study. Wave 5. 2021.	Surveys	1090- adults aged 18 or more who has tested positive for Covid-19 and were at the end of their 10-day self-isolation period.	7 -12 June 2021	Mid pandemic	England	Isolation	
41	UK Health Security Agency (confidential internal document). Office for National Statistics- Test and Trace Cases Insights Study. Wave 6. 2021.	Surveys	936- adults aged 18 or more who has tested positive for Covid-19 and were at the end of their 10-day self-isolation period.	5 -10 July 2021	Mid pandemic	England	Isolation	
42	UK Health Security Agency (confidential internal document). Serial testing of contacts in the context of institutions. 2020.	Interviews	9 - Full-time, part-time, contractors, freelancers, zero-hour contract who cannot work from home or a mix of working from home and outside.<br><br>Mixed experience with Covid-19 test, trace and self-isolation.	3 – 4 December 2020	Mid pandemic	England	Testing & isolation	Initial objectives included test to enable, improve case identification using LFDs for contacts and enable people to

For peer review only - http://bmjopen.bmj.com/site/about/guidelines.xhtml

								avoid unnecessary self-isolation.
43	UK Health Security Agency (confidential internal document). Public perceptions overview. Data from Public Perceptions Tracker 2021.	Surveys	around 1,000 each week - nationally representative sample of adults aged 18 or more	November 2021 report	Late pandemic	England	Testing & reporting	After a short increase, negative media recall of NHSTT appears to be falling. Trust in testing continues to fluctuate.
44	UK Health Security Agency (confidential internal document). Public Sector Self-Collect Evaluation – (Draft) Final Report.	Survey, interview, and focus group	not clear - Public sector employees	1st March 2021 to 30th June 2021	Mid pandemic	UK	Testing & reporting	This report evaluates the roll-out of Self-Test Collect (STC) in the public sector workplace and how it performed against the existing Assisted Test Sites (ATS) model for the public sector workplace
45	UK Health Security Agency (confidential internal document). Research exploring the barriers and drivers to following guidance. nd.	Focus Groups	6 focus groups, 6 interviews with 18–24-year-olds - X 2 groups of people most engaged with and following guidance<br>X 2 groups of people following guidance in a more Ad-hoc manner, picking and choosing<br>X2 groups of people least engaged with guidance and least likely to follow it<br><br>X6, 60-minute depth interviews with 18-24 y/Os	10th January and w/c 17th January 2022	Late pandemic	England	Testing & reporting	At time of research, long list of guidelines to consider, from when to wear a face covering, vaccinations, testing, isolation and what to do if you are a COVID contact.
46	UK Health Security Agency (confidential internal document). Serial contact testing pilots evaluation summary.	Survey, interview, and focus group	n/a – employees	January - Mach 2021	Mid pandemic	England	Testing & isolation	Evaluation summary document.
47	UK Health Security Agency (confidential internal document). Summary Evaluation Findings: Venue Alerts.	Mixed methods	n/a - Recipients of venue alerts in England	24 September 2020 - 19 July 2021	Mid pandemic	England	Isolation	Venues displayed an NHS QR code and kept a record of contacts details for customers,

For peer review only

								visitors and staff who did not sign in through the NHS Covid-19 app. These allowed people to register their attendance so that if a potential outbreak was later identified, others who were present could be notified of their potential risk with a 'venue alert'.
48	UK Health Security Agency (confidential internal document). Supporting COVID-19 testing and vaccination in a deprived local authority: A case study of Blackpool. 2021	Interviews	8 - Four members of Blackpool public health team, the programme director of a large VCFS organisation and 3 Community Champions	May and June 2021	Mid pandemic	England	Testing	Experiences of those working in Blackpool Council and its public health team in supporting testing and vaccination among vulnerable groups, as well as some of the local responses implemented to overcome challenges
49	UK Health Security Agency (confidential internal document). Test and Trace Contacts Insights Study: Wave 3. nd.	Surveys	1100 - adults aged 18 or more who has tested positive for Covid-19 and were at the end of their 10-day self-isolation period.	1 - 10 April 2021	Mid pandemic	England	Isolation	Who had been in contact with someone who has tested positive for coronavirus (COVID-19) and were at the end of their 10-day self-isolation period.
50	UK Health Security Agency (confidential internal document). Test and Trace Contacts Insights Study: Wave 4. nd.	Surveys	1194 - adults aged 18 or more who has tested positive for Covid-19 and	19 - 24 April 2021	Mid pandemic	England	Isolation	

For peer review only - http://bmjopen.bmj.com/site/about/guidelines.xhtml

			were at the end of their 10-day self-isolation period.					
51	UK Health Security Agency (Confidential internal document), Test seeking behaviours; attitudes, barriers and facilitators; Briefing Note, 22.03.21. 2021.	Other	NA: This is a literature revie	NA	NA	UK	Testing	Universal testing
52	UK Health Security Agency (confidential internal document). The effects of negative asymptomatic test result messages on understanding of residual risk and behavioural intentions. 2021.	Surveys	1200 - Online experiment (12-15th March 2021) with a representative sample of the UK population where participants imagined they had taken an LFT as part of an asymptomatic testing programme	12 - 15 March 2021	Mid pandemic	UK	Other	Some receiving a test-negative result from asymptomatic lateral flow testing may misunderstand this to mean no risk of being infectious, resulting in reduced adherence to guidelines Tested whether communicating residual risk information and behavioural implications in negative test result messages affects residual risk understanding and behavioural intentions.
53	UK Health Security Agency (confidential internal document). Understanding Compliance with Self- Isolation: Summary of Survey Findings 2021.	Surveys	42,892 contacts were invited and 6,813 responded. - Over 18 years contacts who were reached and advice by NHS Test and Trace to isolate.	25 August - 14 September 2020	Early – Mid pandemic	UK	Isolation	Universal Testing.
54	UK Health Security Agency (confidential internal document). Understanding experiences of the Test and Trace Support Payment: A Qualitative Follow-up Study. 2021.	Interviews	31 participants - ONS Cases and Contacts Insight Survey participants and were not asked to take part in further research as a result.	1 May 2021	Mid pandemic	England	Isolation	Follow-up qualitative study to the ONS Cases and Contact Insights Survey. Sampled from both confirmed cases

For peer review only

								and contacts of COVID-19.
55	UK Health Security Agency (confidential internal document), Understanding the impact of a COVID-19 outreach programme delivered by voluntary and community sector organisations (VCSOs) for migrant worker communities and the role of VCSOs in communicating public health messaging. 2022.	Interviews	23 service users and 8 staff members from 4 VCSOs - Voluntary and community sector organisation (VCSO) service users and stakeholders.	17 January - 31 March 2022	Late pandemic	England	Testing & isolation	Aimed to communicate coronavirus (COVID-19) guidance on testing, self-isolation, and vaccination to migrant worker communities to improve understanding and encourage uptake.
56	UK Health Security Agency (confidential internal document). Weekly TIEB updates. 12 March 2021. 2021.	Survey, interview, and focus group	n/a - Private, Public sectors employees, NHS employees	8 - 12 March 2021	Mid pandemic	England	Testing & isolation	Pilots' evaluation update document covering operational feasibility, behaviours, socioeconomic impact.
57	UK Health Security Agency (confidential internal document). YouGov- NHS Test and Trace W50 & W51. 2023.	Surveys	2222 adults - England adults (aged 18+)	6 - 19 December 2021	Late pandemic	England	Testing, reporting & isolation	This survey has been conducted using an online interview administered to members of the YouGov Plc UK panel of 800,000+ individuals who have agreed to take part in surveys.
**Schools setting**								
**Literature search**								
1	Chantziara S, Brigden LCA, McCallum CH, Craddock IJ. Using Digital Tools for Contact Tracing to Improve COVID-19 Safety in Schools: Qualitative Study Exploring Views and Experiences Among School Staff. JMIR Form Res. 2022;6(11):e36412.	Interviews	18 staff (4 senior management, 12 teachers, 1 teaching assistant, and 1 behaviour support manager)	4 June - 7 November 2020	Early- Mid pandemic	England	Other	Background to the study was whether schools required support to implement guidelines.

For peer review only - http://bmjopen.bmj.com/site/about/guidelines.xhtml

2	Woodland L, Mowbray F, Smith LE, Webster RK, Amlôt R, Rubin GJ. What influences whether parents recognise COVID-19 symptoms, request a test and self-isolate: A qualitative study. PLoS One. 2022;17(2):e0263537.	Interviews	18 parents of school-aged (4 to 18 years) children	30 November – 11 December 2020	Mid pandemic	England	Testing & isolation	Parents have additional responsibility of using test and trace systems on behalf of their children. Factors associated to use of England's NHS Test and Trace service.
**Evidence overlapping between general and schools service settings**								
1	Nyashanu M, Pfende F, Ekpenyong M. Exploring the challenges faced by frontline workers in health and social care amid the COVID-19 pandemic: experiences of frontline workers in the English Midlands region, UK. J Interprof Care. 2020;34(5):655-61.	Interviews	40 health and social care frontline workers, including 15 nurses, 10 managers and 15 support workers in the English Midlands	February 2020 to April 2020	Early pandemic	England	Testing	Data collected early in the pandemic before asymptomatic testing of healthcare workers was in place, and healthcare workers and patients were prioritised for symptomatic PCR testing
2	Watson D, Baralle NL, Alagil J, Anil K, Ciccognani S, Dewar-Haggart R, Fearn S, Groot J, Knowles K, Meagher C, McGrath C, Muir S, Musgrove J, Glyn-Owen K, Woods-Townsend K, Mortimore A, Roderick P, Baird J, Inskip H, Godfrey K, Barker M. How do we engage people in testing for COVID-19? A rapid qualitative evaluation of a testing programme in schools, GP surgeries and a university. BMC Public Health. 2022;22(1):305.	Interviews and focus groups	210 participants from 4 schools, 1 university and 2 general practices in the South East of England, participating in the Southampton COVID-19 Testing Pilot Programme: 8 general practice staff, 30 pupils, 21 school staff, 12 pupil/parent pairs, 13 parents, 12 senior school representatives, 81 university students, 28 university staff and 5 senior university representatives<br><br>In total: 77 interviews and 20 focus groups	4 June 2020 - 07 November 2020	Early Mid pandemic	England	Testing and isolation	Spoke to i) individuals who had been approached about taking part in the pilot Saliva Testing Programme including some who tested positive; ii) those who were approached but declined to take part; and iii) senior university, primary care and school representatives responsible for delivering the

For peer review only - http://bmjopen.bmj.com/site/about/guidelines.xhtml

								Saliva Testing Programme in their organisations.
**Google/ Bibliographic review- Schools**								
1	Godfrey K, Bagust L, Baird J, Barker M, Batchelor J, Bryant S. Evaluation of the expanded Southampton pilot study (Phase 2) for use of saliva-based lamp testing in asymptomatic populations: Final report, 16th November 2020. 2020.	Interviews and focus groups	Staff, students/pupils, and contractors at four schools (an infant, junior, primary, and secondary) n= 88 and at the University of Southampton were included n= 108	September 2020 - November 2020	Mid pandemic	England	Testing, reporting & Isolation	Purpose of the report is to describe the programme and provide a form of manual as guidance for groups undertaking similar testing.
**Stakeholder identified sources (UKHSA Secretariat)**								
1	UK Health Security Agency (confidential internal document). 2021. Secondary schools research - Wave 2, Birmingham 2021.	Focus groups	Mini groups of parents, teachers, and pupils from a range of schools in Birmingham which either encouraged testing or not	November 2021	Mid pandemic	England	Testing, reporting & isolation	Focus is on schools where testing is less encouraged, and parents/children are not testing regularly.
2	UK Health Security Agency (confidential internal document). 2020. School pilots experience research - User research insights and recommendations.	Survey, Interviews and focus groups	Conducted in-depth interviews with participating school staff, pupils, and parents across 9 schools. 15 in-depth interviews and 796 survey respondents	October - December 2020	Mid pandemic	England	Testing, reporting & isolation	Study conducted alongside school mass testing pilots.
**Healthcare**								
**Literature search**								
1	Martindale AM, Pilbeam C, Mableson H, Tonkin-Crine S, Atkinson P, Borek A, Lant S, Gobat N, Solomon T, Sheard S. Perspectives on COVID-19 testing policies and practices: a qualitative study with scientific advisors and NHS health care workers in England. BMC Public Health. 2021;21(1):1216.	Interviews	24 interviews with 13 participants: five senior scientific advisors (all male Caucasian) and eight healthcare workers (five male, four females, all Caucasian) including nurses, physicians, general practitioners, and allied healthcare workers	March - August 2020	Early pandemic	England	Testing, reporting and isolation	Focus on perceptions of healthcare workers and scientific advisors, with data collected early in the pandemic before asymptomatic testing of healthcare workers was in place, and

For peer review only - http://bmjopen.bmj.com/site/about/guidelines.xhtml

								healthcare workers and patients were prioritised for symptomatic PCR testing.
2	Vindrola-Padros C, Andrews L, Dowrick A, Djellouli N, Fillmore H, Bautista Gonzalez E, Javadi D, Lewis-Jackson S, Manby L, Mitchinson L, Mulcahy Symmons S, Martin S, Regenold N, Robinson H, Sumray K, Singleton G, Syversen A, Vanderslott S, Johnson G. Perceptions and experiences of healthcare workers during the COVID-19 pandemic in the UK. BMJ Open. 2020;10(11):e040503.	Mixed methods	30 interviews with healthcare staff members<br>101 newspaper articles and 146 000 social media posts to capture the direct or indirect perceptions and experiences of healthcare workers	December 2019 to the end of April 2020<br><br>Interviews took place in April 2020	Early pandemic	UK	Testing	Focus on healthcare worker perceptions and experiences during the first wave, with data collected before asymptomatic testing was available but healthcare workers and patients were prioritised for PCR testing
**Stakeholder identified sources (UKHSA Secretariat)**								
1	Bow SMA, Goddard A, Cope G, Sharp N, Schick J, Woods C, Jeffery K, Harrington D, Williams S, Rodger AJ, Finer S, Fowler T, Hopkins S, Tunkel SA. An evaluation of a pilot of daily testing of SARS-CoV-2 contacts in acute hospital and ambulance trusts in England. Public Health. 2022;209:46-51.	Surveys and interviews	60 healthcare workers completed the survey (58 who did daily contact testing and 2 who did not).<br>28 interviews included trust daily contact testing leads, a union representative and two participants per trust. Healthcare workers were from four large, multisite acute hospital trusts in London, Oxford and Lancashire and a London ambulance trust	9 January - 28 February 2021	Mid pandemic	England	Testing a nd isolation	Data collected within the evaluation period when asymptomatic testing of healthcare workers was in effect.<br><br>This paper focuses on daily contact testing rather than routine asymptomatic testing.
**ASC academic papers**								
**Literature search**								
1	Kierkegaard P, Micocci M, McLister A, Tulloch JSP, Parvulescu P, Gordon AL, Buckle P. Implementing lateral flow devices in long-term care facilities: experiences from the Liverpool	Interviews	15 staff from 9 care homes<br>~ Broad representation of staff working in a range of care homes (residential and nursing homes)	December 2020 –	Mid pandemic	England	Testing	Pilot that largely mirrors protocols

For peer review only - http://bmjopen.bmj.com/site/about/guidelines.xhtml

	COVID-19 community testing pilot in care homes- a qualitative study. BMC Health Serv Res. 2021;21(1):1153.		~ Staff who had received training on how to use the LFDs, were directly involved in working with the LFDs to administer visitor and staff testing, and had been working at the care homes prior to the first national lockdown in March 2020, with the rationale that such staff would have a longitudinal perspective from working before and during the pandemic, on how different testing regimes had influenced care home work, and vice versa.	January 2021				adopted in the UK at that time Sample focused on nursing homes Same time as initial vaccine rollout
2	Micocci M, Gordon AL, Allen AJ, Hicks T, Kierkegaard P, McLister A, Walne S, Hayward G, Buckle P. COVID-19 testing in English care homes and implications for staff and residents. Age Ageing. 2021;50(3):668-72.	Interviews	10 staff members from eight care homes in England —with more than 5 years' experience in the sector	July – August 2020	Early pandemic	England	Testing and reporting	The paper describes PCR testing and the feasibility of point of care testing to support the complexity of testing and detection of COVID-19.
3	Tulloch JSP, Micocci M, Buckle P, Lawrenson K, Kierkegaard P, McLister A, Gordon AL, García-Fiñana M, Peddie S, Ashton M, Buchan I, Parvulescu P. Enhanced lateral flow testing strategies in care homes are associated with poor adherence and were insufficient to prevent COVID-19 outbreaks: results from a mixed methods implementation study. Age and Ageing. 2021;50(6):1868-75.	Interviews	15 care home staff members (from 9 care homes)	December 2020 - January 2021	Mid pandemic	England	Testing and Isolation	Pilot that largely mirrored protocols adopted in the UK at that time. Sample focused on nursing homes Same time as initial vaccine rollout
**Google/ Bibliographic review**								
1	Nyashanu M, Pfende F, Ekpenyong MS. Triggers of mental health problems among frontline healthcare workers during the COVID-19 pandemic in private care homes and domiciliary care agencies: Lived experiences of care workers in the Midlands region, UK. Health Soc Care Community. 2022;30(2):e370-e6.	Interviews	40 semi-structured interviews with frontline workers from nursing homes and domiciliary care agencies	Not stated - but before May 2020	Early pandemic	England	Testing & Isolation	Early stages of pandemic, frontline health workers and patients adversely affected.

For peer review only - http://bmjopen.bmj.com/site/about/guidelines.xhtml

**Stakeholder identified sources (UKHSA Secretariat)**									
1	UK Health Security Agency (confidential internal document). 2022. Adult Social Care Vaccination & COVID-19 Testing Report.	Surveys	651 adult social care workers including those in care homes, domiciliary and supported living	27 April – 16 May 2022	Late pandemic	England	Testing, reporting, and isolating	Survey conducted following removal of vaccinations as requirement for deployment in adult social care. Further testing changes in April 2022 (outside of evaluation period).
2	UK Health Security Agency (confidential internal document). nd. Baselining the asymptomatic staff testing journey in adult social care (ASC)	Other	Spoke to stakeholders (# undefined) to create high-level and detailed end-to-end service maps<br>Reviewed existing research to identify user needs and mapped user pain points<br>Created a content inventory of adult social care testing guidance and plotted key documents onto the service map<br>Conducted a SWOT analysis (strengths, weaknesses, opportunities, and threats) to identify gaps and opportunities	Undefined	Unclear	England	Testing and reporting	Non-specific as timeline not known. Expected that due to detail, this is after introduction of twice weekly LFD + PCR testing
3	RWD002 Report: Root cause analysis of observed sensitivity of LFDs below that of pre-deployment expected baseline performance when used by Adult Social Care staff	Other	Dual tests data collected between December 2020 and December 2021<br>Reconciled and analysed to explore differences (if any) device sensitivity within the population user group<br>Reviewed Innova and Biotime LFD devices	24 December 2020 – 23 December 2021	Mid pandemic	England	Reporting and Isolation	Generally, when staff followed testing regimen, LFD sensitivity matched baseline expectation. Recognition that funding was available to support self-isolation, with acknowledgement that decisions made at LA level are not reviewed.

For peer review only - http://bmjopen.bmj.com/site/about/guidelines.xhtml

Supplemental Table 5. Definitions of the stages of the pandemic

Stage	Date range	Notable events
**1. Early stage pandemic**	February 2020 to August 2020	• First lockdown<br>• Introduction of self-isolation rules<br>• Access to PCR testing established and gradually increased<br>• Restrictions from first lockdown eased
**2. Mid-stage pandemic**	September 2020 to November 2021	• Increasing restrictions leading to two more national lockdowns<br>• NHS COVID app launches<br>• Introduction of COVID-19 vaccines<br>• Access to lateral flow device self-test kits for general population<br>• Four-stage roadmap to easing restrictions leading to most legal limits removed by end of July 2021
**3. End-stage pandemic**	December 2021 to March 2022	• Omicron variant spread leads to increase in vaccine boosters, and self-isolation rule changes<br>• February 2021 sees the removal of legal requirement to self-isolate (unvaccinated) and an end to contact tracing

For peer review only - http://bmjopen.bmj.com/site/about/guidelines.xhtml

Supplemental Figure 1. Infographic to define the stages of the pandemic, with details.

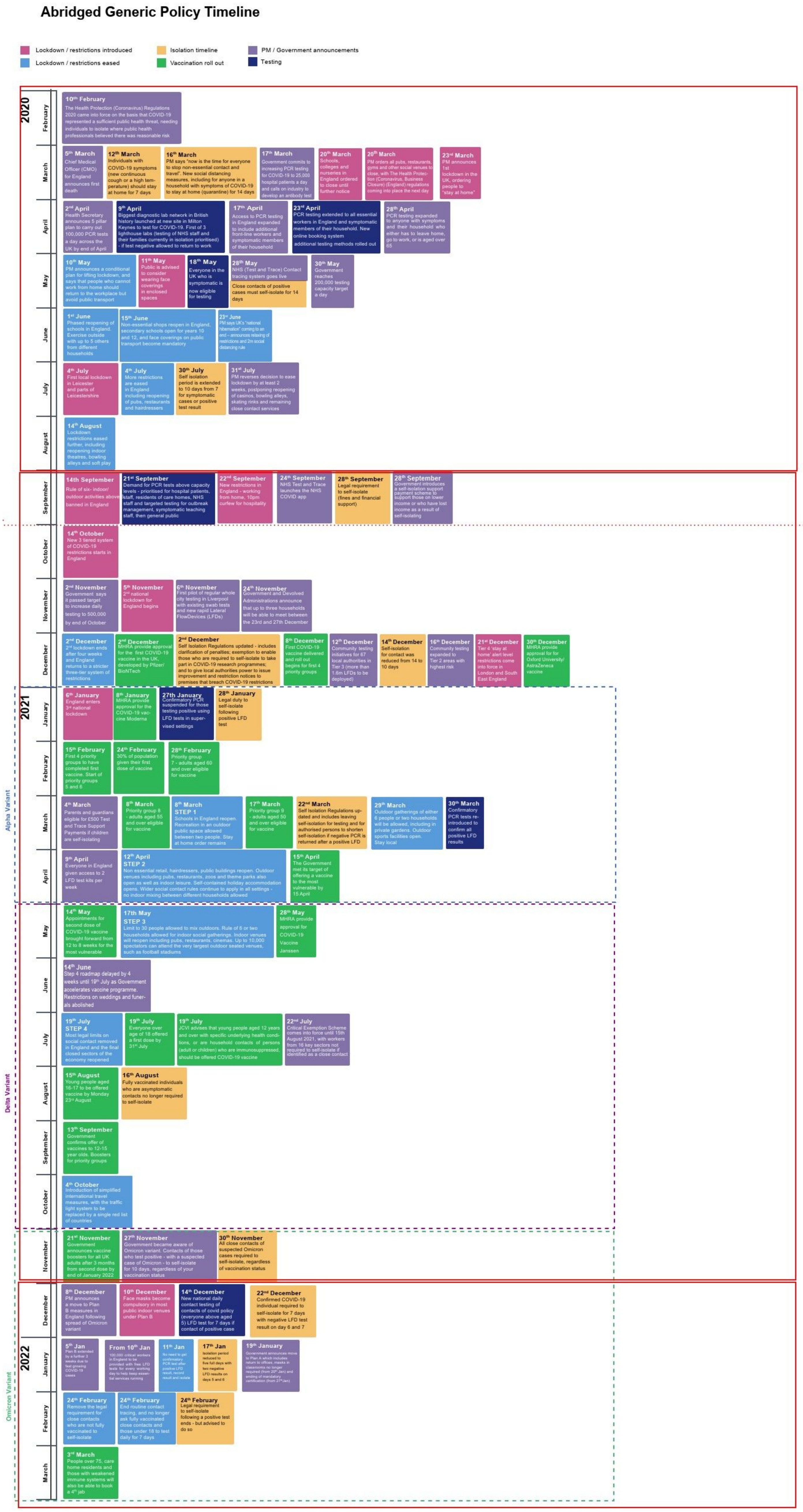

1. Early stage Pandemic

- First lockdown
- Introduction of self-isolation rules
- Access to PCR testing established and gradually increased
- Restrictions from first lockdown eased

2. Mid-stage Pandemic

- Increasing restrictions leading to two more national lockdowns
- NHS COVID app launches
- Introduction of COVID vaccines
- Access to LFD self-test kits for general population
- 4 stage roadmap to easing restrictions leading to most legal limits removed by end of July 2021

3. End stage of pandemic

- Omicron variant spread leads to increase in vaccine boosters and self isolation rule changes
- February 2021 sees the removal of legal requirement to self isolate (unvaccinated) and an end to contact tracing

Supplemental References

1. National Center for Biotechnology Information (NCBI). PubMed Overview: NCBI; 2023 [cited 2023 28 August]. Available from: https://pubmed.ncbi.nlm.nih.gov/about/.
2. Elsevier. Scopus. Your brilliance, connected: Elsevier; 2022 [cited 2023 28 August]. Available from: https://www.elsevier.com/en-gb/solutions/scopus.
3. Jose Garnica C. The WHO COVID-19 Research Database: behind the scenes of a super powerful tool! Library Science Talk2022.
4. Hanneke CR, Asada Y, Lieberman L, Neubauer LC, Fagen M. The Scoping Review Method: Mapping the Literature in " Structural Change " Public Health Interventions. SAGE Research Methods Cases Part 2. 2017. doi: https://dx.doi.org/10.4135/9781473999008.

For peer review only

For peer review only - http://bmjopen.bmj.com/site/about/guidelines.xhtml

Preferred Reporting Items for Systematic reviews and Meta-Analyses extension for Scoping Reviews (PRISMA-ScR) Checklist

SECTION	ITEM	PRISMA-ScR CHECKLIST ITEM	REPORTED ON PAGE #
**TITLE**			
Title	1	Identify the report as a scoping review.	1
**ABSTRACT**			
Structured summary	2	Provide a structured summary that includes (as applicable): background, objectives, eligibility criteria, sources of evidence, charting methods, results, and conclusions that relate to the review questions and objectives.	2
**INTRODUCTION**			
Rationale	3	Describe the rationale for the review in the context of what is already known. Explain why the review questions/objectives lend themselves to a scoping review approach.	3
Objectives	4	Provide an explicit statement of the questions and objectives being addressed with reference to their key elements (e.g., population or participants, concepts, and context) or other relevant key elements used to conceptualize the review questions and/or objectives.	3
**METHODS**			
Protocol and registration	5	Indicate whether a review protocol exists; state if and where it can be accessed (e.g., a Web address); and if available, provide registration information, including the registration number.	3
Eligibility criteria	6	Specify characteristics of the sources of evidence used as eligibility criteria (e.g., years considered, language, and publication status), and provide a rationale.	4 and supplementary material table 2
Information sources*	7	Describe all information sources in the search (e.g., databases with dates of coverage and contact with authors to identify additional sources), as well as the date the most recent search was executed.	4 and supplementary material
Search	8	Present the full electronic search strategy for at least 1 database, including any limits used, such that it could be repeated.	Supplementary material table 1
Selection of sources of evidence†	9	State the process for selecting sources of evidence (i.e., screening and eligibility) included in the scoping review.	4
Data charting process‡	10	Describe the methods of charting data from the included sources of evidence (e.g., calibrated forms or forms that have been tested by the team before their use, and whether data charting was done independently or in duplicate) and any processes for obtaining and confirming data from investigators.	4-5
Data items	11	List and define all variables for which data were sought and any assumptions and simplifications made.	4-5 and supplementary material table 3

For peer review only - http://bmjopen.bmj.com/site/about/guidelines.xhtml

SECTION	ITEM	PRISMA-ScR CHECKLIST ITEM	REPORTED ON PAGE #
Critical appraisal of individual sources of evidence§	12	If done, provide a rationale for conducting a critical appraisal of included sources of evidence; describe the methods used and how this information was used in any data synthesis (if appropriate).	NA
Synthesis of results	13	Describe the methods of handling and summarizing the data that were charted.	4-5
**RESULTS**			
Selection of sources of evidence	14	Give numbers of sources of evidence screened, assessed for eligibility, and included in the review, with reasons for exclusions at each stage, ideally using a flow diagram.	5 and figure 2
Characteristics of sources of evidence	15	For each source of evidence, present characteristics for which data were charted and provide the citations.	5 and supplementary material table 4
Critical appraisal within sources of evidence	16	If done, present data on critical appraisal of included sources of evidence (see item 12).	NA
Results of individual sources of evidence	17	For each included source of evidence, present the relevant data that were charted that relate to the review questions and objectives.	5-9
Synthesis of results	18	Summarize and/or present the charting results as they relate to the review questions and objectives.	5 and figure 3
**DISCUSSION**			
Summary of evidence	19	Summarize the main results (including an overview of concepts, themes, and types of evidence available), link to the review questions and objectives, and consider the relevance to key groups.	9-11
Limitations	20	Discuss the limitations of the scoping review process.	2 and 11
Conclusions	21	Provide a general interpretation of the results with respect to the review questions and objectives, as well as potential implications and/or next steps.	11
**FUNDING**			
Funding	22	Describe sources of funding for the included sources of evidence, as well as sources of funding for the scoping review. Describe the role of the funders of the scoping review.	12

JBI = Joanna Briggs Institute; PRISMA-ScR = Preferred Reporting Items for Systematic reviews and Meta-Analyses extension for Scoping Reviews.

* Where *sources of evidence* (see second footnote) are compiled from, such as bibliographic databases, social media platforms, and Web sites.

† A more inclusive/heterogeneous term used to account for the different types of evidence or data sources (e.g., quantitative and/or qualitative research, expert opinion, and policy documents) that may be eligible in a scoping review as opposed to only studies. This is not to be confused with *information sources* (see first footnote).

‡ The frameworks by Arksey and O'Malley (6) and Levac and colleagues (7) and the JBI guidance (4, 5) refer to the process of data extraction in a scoping review as data charting.

§ The process of systematically examining research evidence to assess its validity, results, and relevance before using it to inform a decision. This term is used for items 12 and 19 instead of "risk of bias" (which is more applicable to systematic reviews of interventions) to include and acknowledge the various sources of evidence that may be used in a scoping review (e.g., quantitative and/or qualitative research, expert opinion, and policy document).

St. Michael's
Inspired Care.
Inspiring Science.

For peer review only - http://bmjopen.bmj.com/site/about/guidelines.xhtml

From: Tricco AC, Lillie E, Zarin W, O'Brien KK, Colquhoun H, Levac D, et al. PRISMA Extension for Scoping Reviews (PRISMAScR): Checklist and Explanation. Ann Intern Med. 2018;169:467–473. doi: 10.7326/M18-0850.

For peer review only

BMJ Open

Decision to self-isolate during the COVID-19 pandemic in the UK: a rapid scoping review

Journal:	*BMJ Open*
Manuscript ID	bmjopen-2024-084437.R1
Article Type:	Original research
Date Submitted by the Author:	26-Feb-2024

Complete List of Authors:	Keene, Claire; University of Oxford, Centre for Global Health Research, Nuffield Department of Medicine<br>Dickinson, Sophie; Ernst & Young (EY) , UKI Health Sciences and Wellness<br>Naidoo, Reshania; University of Oxford, Nuffield Department of Medicine; Ernst & Young (EY) , UKI Health Sciences and Wellness<br>Andersen-Waine, Billie; Ernst & Young (EY) , UKI Health Sciences and Wellness<br>Ferguson-Lewis, Angus; Ernst & Young (EY) , UKI Health Sciences and Wellness<br>Polner, Anastasia; Ernst & Young (EY) , UKI Health Sciences and Wellness<br>Amswych, Ma'ayan; Ernst & Young (EY) , UKI Health Sciences and Wellness<br>White, Lisa ; University of Oxford, Department of Biology<br>Molyneux, Sassy ; University of Oxford, Centre for Global Health Research, Nuffield Department of Medicine; University of Oxford, Pandemic Sciences Institute<br>Wanat, Marta; University of Oxford, Nuffield Department of Primary Care Health Sciences<br>EY-Oxford Health Analytics Consortium, NA; EY-Oxford Health Analytics Consortium

**Primary Subject Heading**:	Public health
Secondary Subject Heading:	Health policy, Infectious diseases, Health services research, Qualitative research
Keywords:	COVID-19, Behavior, Public health < INFECTIOUS DISEASES, Protocols & guidelines < HEALTH SERVICES ADMINISTRATION & MANAGEMENT

SCHOLARONE™ Manuscripts

I, the Submitting Author has the right to grant and does grant on behalf of all authors of the Work (as defined in the below author licence), an exclusive licence and/or a non-exclusive licence for contributions from authors who are: i) UK Crown employees; ii) where BMJ has agreed a CC-BY licence shall apply, and/or iii) in accordance with the terms applicable for US Federal Government officers or employees acting as part of their official duties; on a worldwide, perpetual, irrevocable, royalty-free basis to BMJ Publishing Group Ltd ("BMJ") its licensees and where the relevant Journal is co-owned by BMJ to the co-owners of the Journal, to publish the Work in this journal and any other BMJ products and to exploit all rights, as set out in our licence.

The Submitting Author accepts and understands that any supply made under these terms is made by BMJ to the Submitting Author unless you are acting as an employee on behalf of your employer or a postgraduate student of an affiliated institution which is paying any applicable article publishing charge ("APC") for Open Access articles. Where the Submitting Author wishes to make the Work available on an Open Access basis (and intends to pay the relevant APC), the terms of reuse of such Open Access shall be governed by a Creative Commons licence – details of these licences and which Creative Commons licence will apply to this Work are set out in our licence referred to above.

Other than as permitted in any relevant BMJ Author's Self Archiving Policies, I confirm this Work has not been accepted for publication elsewhere, is not being considered for publication elsewhere and does not duplicate material already published. I confirm all authors consent to publication of this Work and authorise the granting of this licence.

For peer review only

Decision to self-isolate during the COVID-19 pandemic in the UK: a rapid scoping review

Co-authors: Claire M Keene[1,†], Sophie Dickinson[2], Reshania Naidoo[2,3], Billie Andersen-Waine[2], Angus Ferguson-Lewis[2], Anastasia Polner[2], Ma'ayan Amswych[2], Lisa J White[4], Sassy Molyneux[1,5], Marta Wanat[6], and the EY-Oxford Health Analytics Consortium[*]

[1] Health Systems Collaborative, Oxford Centre for Global Health Research, Nuffield Department of Medicine, University of Oxford, Oxford, UK
[2] Ernst & Young (EY) UKI Health Sciences and Wellness, London, UK
[3] Centre for Tropical Medicine & Global Health, Nuffield Department of Medicine, University of Oxford, Oxford, UK
[4] Department of Biology, University of Oxford, Oxford, UK
[5] Pandemic Sciences Institute, University of Oxford, UK
[6] Nuffield Department of Primary Care Health Sciences, University of Oxford, UK

[†]Corresponding author: Claire M Keene: clairekeene@gmail.com
[*]EY-Oxford Health Analytics Consortium membership:

- Ricardo Aguas - OUI
- Ma'ayan Amswych - EY
- Sumali Bajaj - OUI
- Kweku Bimpong - EY
- Adam Bodley - OUI
- Liberty Cantrell - OUI
- Siyu Chen - OUI
- Richard Creswell - OUI
- Prabin Dahal - OUI
- Sophie Dickinson - EY
- Sabine Dittrich - OUI
- Tracy Evans - OUI
- Angus Ferguson-Lewis - EY
- Caroline Franco - OUI
- Bo Gao - OUI
- Rachel Hounsell - OUI
- Muhammad Kasim - OUI
- Claire Keene - OUI
- Ben Lambert - OUI
- Umar Mahmood - EY
- Melinda Mills - OUI
- Ainura Moldokmatova - OUI
- Sassy Molyneux - OUI
- Randolph Ngwafor Anye - OUI
- ared Norman - OUI
- Wirichada Pan-Ngum - OUI
- Sunil Pokharel - OUI
- Anastasiia Polner - EY
- Katarzyna Przybylska - OUI
- Sompob Salamba - OUI
- Sheetal Silal - OUI
- oseph Tsui - OUI
- Merryn Voysey - OUI
- Marta Wanat - OUI

Contact emails for submission:
Sophie Dickinson <Sophie.Dickinson@uk.ey.com>;
Reshania Naidoo <reshania.naidoo@ndm.ox.ac.uk>;
Billie Andersen-Waine <b@billieandersen.com>;
Angus Ferguson-Lewis <Afergusonlewis@gmail.com>;
Anastasiia Polner <Anastasiia.Polner@ey-seren.com>;
Ma'ayan Amswych <Maayan.Amswych@uk.ey.com>;
Lisa J White <lisa.white@biology.ox.ac.uk>;
Sassy Molyneux <smolyneux@kemri-wellcome.org>;
Marta Wanat <marta.wanat@phc.ox.ac.uk>

Word count: 4770 words
3 figures 1 table
Abstract 300/300 words

For peer review only

For peer review only - http://bmjopen.bmj.com/site/about/guidelines.xhtml

Behavioural lit review: ISOLATION manuscript

Key words: COVID-19, Test trace and isolate, England, isolation behaviour

For peer review only

For peer review only - http://bmjopen.bmj.com/site/about/guidelines.xhtml

Abstract

Objective: Testing for COVID-19 was a key component of the United Kingdom's (UK) response to the COVID-19 pandemic. This strategy relied on positive individuals self-isolating to reduce transmission, making isolation the lynchpin in the public health approach. Therefore, we scoped evidence to systematically identify and categorise barriers and facilitators to compliance with self-isolation guidance during the COVID-19 pandemic in the UK, to inform public health strategies in future pandemics.

Design: Scoping review

Search strategy: Key terms were used to search literature databases (PubMed, Scopus, and the World Health Organization COVID-19 Research Database, on 7 November 2022), Google Scholar, and stakeholder-identified manuscripts, ultimately including evidence published in English from UK-based studies conducted between 2020 and 2022.

Data extraction and synthesis: Data were extracted and synthesised into themes, organised broadly into capability, opportunity, and motivation, and reviewed with key stakeholders from the UK Health Security Agency (UKHSA).

Results: We included 105 sources, with 63 identified from UKHSA and used to inform their decision-making during the pandemic. Influences on the decision to comply with isolation guidance were categorised into six themes: perceived ability to isolate; information and guidance; logistics; social influences, including trust; perceived value; and perceived consequences. Individuals continuously assessed these factors in deciding whether or not to comply with guidance and self-isolate.

Conclusions: Decisions to self-isolate after a positive test were influenced by multiple factors, including individuals' beliefs, concerns, priorities, and personal circumstances. Future testing strategies must facilitate meaningful financial, practical and mental health support to allow individuals to overcome the perceived and actual negative consequences of isolating. Clear, consistent communication of the purpose and procedures of isolating will also be critical to support compliance with self-isolation guidance, and should leverage people's perceived value in protecting others. Building public trust is also essential, but requires investment before the next pandemic starts.

Strengths and limitations

- This review is strengthened through the use of a broad search strategy to identify a wide range of evidence and by engagement with a diverse group of stakeholders to contextualise and accurately interpret the results.
- It is also strengthened through the inclusion of a large body of unpublished and confidential internal data from the UK Health Security Agency, which supplemented the limited published literature and which would not otherwise be available for synthesis, general dissemination, and contribution to discussions on future pandemic strategies.
- However, given the paucity of studies specifically addressing isolation behaviours, much of the published evidence on isolation behaviour in this review was drawn from studies that focused primarily on testing rather than isolation during the COVID-19 pandemic.
- Additionally, the review was conducted over a short period of time by a large team, potentially introducing inconsistencies into the screening and extracting processes despite attempts to mitigate bias, including overlap of screening and extraction between reviewers, quality checks and review of included original documents during write up.

For peer review only - http://bmjopen.bmj.com/site/about/guidelines.xhtml

Introduction

Any novel pandemic poses a particular challenge in that definitive pharmaceutical interventions, such as therapeutic medications or preventative vaccines, take time to develop.(1) In the meantime, public health policy is reliant on non-pharmaceutical interventions to reduce transmission and lessen impact.(1, 2) In the United Kingdom (UK), the initial response to COVID-19 centred on a spectrum of non-pharmaceutical public health measures aimed at mitigating transmission from COVID-19-positive cases(1, 2): periods of national and local lockdown, social distancing, limits on social gatherings, mandated use of face coverings in public spaces, and self-isolation after a positive COVID-19 test result or contact with a case.(3, 4)

To facilitate isolation, mass testing for COVID-19 was required to identify COVID-19-positive individuals and their close contacts.(5) The National Health Service Test and Trace (NHSTT) programme was established in May 2020 to implement testing, tracing and isolation services at scale.(6) This complex intervention was the first programme of its kind in the UK, created and delivered at pace during a period of unprecedented uncertainty.(5) It distributed nearly two billion lateral flow tests and 158 million polymerase chain reaction tests free of charge in England alone, at a reported cost of £25.8 billion, with the aim of preventing population-wide deaths, decreasing morbidity, reducing burdens on healthcare services, and facilitating the re-opening of the economy. The testing programme detected between 26% and 40% of all COVID-19 cases in England.(5)

The ultimate goal of the testing and tracing activities was to facilitate isolation of those who tested positive for COVID-19 and/or who were close contacts of positive cases; it was through the isolation step of the NHSTT programme's cascade that transmission was reduced.(7) Consequently, regardless of the resources invested in encouraging people to test, if people did not isolate when required, the impact of the programme would be undermined. Despite requirements to self-isolate, compliance was often suboptimal and varied across different contexts and over time.(5) It is crucial to understand what drove adherence with isolation guidance, and what barriers were faced, to inform future pandemic response strategies.

To support the development of more robust public health strategies in future, we conducted a scoping review of the available evidence on perceptions and experiences of self-isolation during the COVID-19 pandemic in the UK. We then categorised the barriers and facilitators to self-isolation identified.

Methods

Study design

A rapid scoping study was conducted to summarise the large volume of rapidly generated, heterogenous evidence on isolation during the COVID-19 pandemic in the UK, to identify gaps in this evidence, and to describe the barriers and facilitators to engaging with self-isolation guidance. In conducting this scoping review we followed the 2005 'Arksey and O'Malley' framework(8) (with the adaptations proposed by Levac et al in 2010(9)) and drew on the 2015 Joanna Briggs Institute guidance.(10)

This scoping study was nested in a broader rapid scoping review of barriers and facilitators to engaging with the COVID-19 testing programme, covering testing, reporting, and self-isolation behaviour in the UK. This in turn formed part of a mixed methods programme of work conducted by the EY-Oxford Health Analytics Consortium, aimed at evaluating the UK Health Security Agency's (UKHSA's) COVID-19 testing strategy in England – the protocol and report are available online.(5, 11)

For peer review only - http://bmjopen.bmj.com/site/about/guidelines.xhtml

Search strategy and selection of the evidence

The search strategy (see supplemental table 1 for search terms) was developed in close consultation with an information specialist and conducted on 7 November 2022 using PubMed, Scopus, the World Health Organization COVID-19 Research Database, and documents provided by UKHSA. UKHSA-provided documents comprising of a body of published and unpublished research; many of these documents were confidential internal documents relating to work specifically conducted to inform UKHSA's decisions during the pandemic. This search was supplemented through free-text searches on Google Scholar, review of the references of included articles, and stakeholder consultation(12) (see supplemental table 2 for the rationale for database selection).

Table 1. Summary of the search parameters and limits as well as the inclusion and exclusion criteria,[2] categorised according to the 'population, context, concept' search framework(13)

	Inclusion criteria	Exclusion criteria
**SEARCH LIMITS**		
**Language**	Published in English	Published in languages other than English
**Dates**	Published from 2020	Published before 2020
**Methods**	Qualitative or mixed methods studies	Purely quantitative studies
**ELIGIBILITY**		
**Literature**	Journal articles, peer-reviewed material, articles under review, published books and book chapters, other academic research, research commissioned by governments, unpublished reports	Opinion or statement pieces, magazine articles, blog posts
**Population**	England, Northern Ireland, Scotland, Wales, and the islands making up the British Isles	Countries outside the UK, including the Republic of Ireland
**Context (service settings)**	All NHSTT programme service settings, including healthcare workers, adult social care, schools, universities, community, and the universal testing programmes.	Nil
**Concept (key activities)**	Description of isolation behaviour and barriers and/or facilitators to adherence to self-isolation guidance (with a focus on isolating due to a positive COVID-19 test result, but including isolating after exposure to a positive contact).	Not focused on the behaviour of self-isolation

Following the database search, all identified citations were collated and uploaded into Rayyan (a web-based research collaboration platform(14)), and duplicates were removed. Following an initial screening pilot, titles and abstracts were screened by two reviewers against the inclusion criteria for the overall project, including testing, reporting, and isolation behaviour. A sample of ≥20% titles and abstracts were evaluated by a third reviewer to clarify eligibility criteria and ensure consistency of inclusion,(9) calculating a Gwet's first-order coefficient (AC1)(15) to assess agreement. Potentially relevant sources were retrieved in full and reviewed against the eligibility criteria. Disagreements at each stage of the selection process were resolved through discussion; discussions were held with an additional reviewer if no consensus was reached. The evidence included in this paper on isolation behaviour was drawn from the documents included in the overarching review (Table 1).

Data extraction, charting and synthesis

To rapidly process a large volume of documents, a team of 12 reviewers extracted the data. Data extracted included i) study metadata and ii) information about the perceptions, experiences, barriers, and facilitators to each of the key activities (testing, reporting, and isolating). Data were extracted into

For peer review only - http://bmjopen.bmj.com/site/about/guidelines.xhtml

an Excel template, refined through entry of initial sources (supplemental table 3). Each reviewer extracted data from two sources that overlapped with sources reviewed by another team member, to check quality and support discussions to refine the template and eligibility criteria. Given the rapid timelines for the overarching programme of work and the aim of this study to scope the evidence, the articles were not assessed for quality.

The analysis combined deductive and inductive elements, organised across three steps. First, a set of a priori categories was developed based on the expertise of the team and an initial reading of included sources. Initial categories of barriers and facilitators were refined throughout the analysis to ensure they reflected the full dataset. Second, the data were synthesised thematically, resulting in some categories being collapsed and reconfigured. Third, the themes were mapped onto the dimensions of the Capability, Opportunity, and Motivation Model of Behaviour (COM-B)(16) (Figure 1), to facilitate selection of potential intervention strategies.

Stakeholder input

Stakeholders from UKHSA were consulted to identify additional sources of published and unpublished evidence (included as 'stakeholder-identified' sources), sense-check the findings, and help interpret, frame and contextualise the results.

Results

Overview of the evidence

After screening 4152 records, this analysis included 105 sources in the synthesis on self-isolation during COVID-19 (see **Error! Reference source not found.**, full details in supplemental table 4). Reasons for exclusion at full-text screening are described in figure 2. Of these 105 studies, 29% were identified through the literature database search, 60% from UKHSA stakeholders, and 11% from other sources (bibliographic review and Google search). Overall, 81% were focused on England, 3% on Scotland, 2% on Wales, and none on Northern Ireland; 12% covered the UK in general; and 2% included the UK in a broader international study. Data were collected during the early stages of the pandemic (before August 2020) in 18% of papers, during the middle stages (up to November 2021) in 75%, and during the final stages of restrictions in 18% (supplemental table 5 and figure 1). Surveys were used in 37% of the sources, interviews in 54%, focus groups in 16%, and other methods in 16%.

Overview of results

We arrived at six themes to categorise the barriers and facilitators that influenced the decision to comply with isolation guidance (**Error! Reference source not found.**): logistics of isolation; social influences and trust; perceived ability to isolate; information and guidance; perceived consequences of complying with isolation; and perceived value of isolating. Ultimately, individuals weighed up these influences in their decision to comply with guidance and self-isolate.

1. Perceived ability to self-isolate

Despite negative descriptions of isolation, many people reported being able to self-isolate in line with contemporary requirements(17-20) and confidence in being able to self-isolate effectively and for the full duration required.(18) However, this varied among people and over time, as well as by the level of adherence to the guidance.(21)

Isolation was described as something that could be prepared for,(22, 23) with previous experiences influencing whether and how individuals planned for potential periods of self-isolation.(23)

For peer review only - http://bmjopen.bmj.com/site/about/guidelines.xhtml

> *I was aware that this could happen at any moment in time, more in actual fact during the winter time we had snow up here in the North of England [where] we got cut off for a few days, I've had flu before so I've always got lots of soup, things in the freezer, it's kind of an eventuality so I didn't even have to ask somebody to go shopping for me, I just said right we're on lockdown until the test comes back so we had five or six days in the house where we just stayed at home*. (Parent 4).(23)

External support reduced the need for people to leave home(22) and reduced reports of experiencing isolation as a challenge.(24) Required support included practical help, food, medical supplies, financial aid (including sick pay), and mental health care (such as welfare check-in calls or activities to distract those isolating).(25-29) Neighbourly support was also described as fostering a sense of community(29); for some university students, the opportunity to isolate together in a single space facilitated mutual support and therefore facilitated self-isolation.(30)

Support came from the government through the Test and Protect system, the Test and Trace Support Payment Scheme,(31) local authority support measures such as shopping assistance,(32, 33) the entitlement to sick pay,(34) and universities.(29) Other support came through friends, family, and community networks.(22, 23, 26, 29) Support was described as particularly important for 'younger people, those with precarious incomes and women',(35) as well as people 'living alone or without existing social connections established in the community'.(36) Those with potentially greater need for support were less likely to report having adequate support.(24)

The uptake of existing support programmes was reduced by lack of awareness(31, 37-39) and access issues,(31, 37) including bureaucratic application processes and stringent eligibility criteria.(37, 40) However, expanding eligibility alone had mixed effects on adherence to self-isolation.(41-43). It was suggested that support should also be widely advertised and proactively signposted through support call-handlers(37, 44) and that reassurance was needed to overcome stigma and some people feeling unable to ask for help.(26)

2. Information and guidance: understanding when, where, how, and why to isolate

Poor understanding or confusion about the guidance and about transmission risks was a barrier to isolating effectively(21, 25, 45-48); reported lack of clarity tended to be in understanding the rules about leaving home for further COVID-19 testing,(18) understanding the duration of isolation and when to start isolating,(45) understanding whether others in the household should isolate,(30) or whether to isolate if asymptomatic.(25, 48) Understanding of guidance decreased when regulations changed.(49) One survey found greater understanding of the guidance among those who did self-isolate than among those who did not comply,(18) but this was not always consistent as experiences were also reported of isolating despite not understanding the guidance.(50, 51)

Good communication was described as an enabler of adherence to isolation guidance,(40) including provision of written information in different languages and welfare check-in calls that increased knowledge of the guidance.(27) Communication of specific content was suggested to improve isolation uptake: providing insight into the importance of adherence to self-isolation guidance, demonstrating that it was the norm and that many people were following the guidance, reinforcing trust in the government and NHS programmes, and advertising available practical and financial support.(37, 52) On the other hand, uncoordinated and inefficient communication during isolation caused frustration.(26)

For peer review only - http://bmjopen.bmj.com/site/about/guidelines.xhtml

3. Logistics of isolation: convenience and practical challenges

People described various practical challenges to following isolation guidance to the letter,(53) including limited access to testing,(25) unsafe or unsuitable home environments, managing childcare and caring responsibilities,(54) the difficulty of managing multiple positive cases or mixed results within a single household,(27, 55) and avoiding other household members becoming sick.(23, 45, 53) These challenges were magnified in smaller houses and apartments(54) or when households included vulnerable family members.(30) Ultimately it appeared that combinations of these challenges heightened barriers to effective isolation.(27)

> *I think it's unrealistic to isolate within the household. If my daughter gets it, it's unrealistic for her to stay in her room and not come out*. (Parent).(45)

4. Social influences and trust in authorities

People perceived high levels of adherence to self-isolation guidance in the community and higher still in their friends and family.(18) It was suggested that the perception that others were following self-isolation guidance, and that isolating when positive was the 'norm', facilitated self-isolation.(52)

Guidance from authoritative sources influenced adherence to self-isolation.(18) However, this deference to authority decreased over time,(56) and distrust in the government was described as a barrier to self-isolation.(25) Disagreement with government measures,(18) mistrust regarding the accuracy of the NHS COVID-19 app and proximity alerts,(23, 54, 57) and concern over secure handling of details by NHSTT seemed to be associated with lower adherence to self-isolation guidance.(18) A lack of trust in test results undermined engagement with isolation; the negative consequences of isolation (outlined below) were considered an unacceptable burden if the requirement to self-isolate might be 'unnecessary' if based on a false-positive result. This was a particular concern among healthcare workers(26) and care home staff.(58) Negative publicity may have undermined trust in testing processes and, in turn, willingness to isolate.(54)

> *'Several of those interviewed were concerned about reports of accuracy, concerning the LFDs [lateral flow devices]. There was a particular concern about false-positive tests and its potential to reduce their already fragile workforce in the event that they ended up having to send "non-infectious" employees home to self-isolate because of faulty test results.'(58)*

5. Perceived value in complying with isolation

Perceived benefit influenced the decision to comply with isolation guidance.(25) People described adherence to isolation guidance as 'important'(18) primarily to prevent transmission and protect others.(23, 26, 59) Adherence to self-isolation even beyond official requirements appeared to be driven by perceived risk of contracting COVID-19 and perceived vulnerability to severe COVID-19.(18, 59) For these reasons, it was suggested that adherence to self-isolation would be supported through 'strengthening perceived benefit to self-isolate with messages emphasising its effectiveness'(25) and that the desire to protect others should be leveraged in communication of guidance.(47)

Some healthcare workers valued self-isolation as a break from heavy workloads(60) and an opportunity to 'engage in new hobbies, talk to family and friends by phone or online'(36) and foster a 'sense of community with others in the same situation'.(29)

In contrast, others felt that there was no benefit to isolating or following the rules, with some reporting it to be a 'waste of time',(61) particularly when they considered themselves low risk due to vaccination, had low-risk contacts, or used infection control measures.(62) This was particularly the

For peer review only - http://bmjopen.bmj.com/site/about/guidelines.xhtml

case after a positive contact rather than after a positive test or if they had symptoms.(63-66) For some, perceived risk decreased over the course of the pandemic,(59) and students felt at lower risk because of their younger age.(29) Additionally, asymptomatic individuals who tested positive had lower motivation to isolate than symptomatic individuals testing positive, as observed by call handlers.(30)

6. Perceived consequences of isolation

Daily life

People described the consequences of self-isolation on their daily life, such as the inability to leave one's house, socialise, or use public transport.(67) This made it difficult to maintain routine domestic tasks (such as shopping, accessing healthcare, collecting medications, or exercising), maintain social ties with family and friends, and to engage with education or work.(23, 26, 27, 33, 36, 45, 68, 69) Parents also anticipated difficulties balancing work and child care obligations when they, or their children, needed to isolate.(23) These difficulties were described as major challenges to adherence to isolation(24) and stated as a reason for non-adherence to isolation guidance.(24, 25, 32, 48)

People described guilt because their isolation would prevent them from performing their usual roles, meaning they were letting people down by not fulfilling their obligations and were placing an additional burden on work colleagues and household members.(27, 53, 68, 70, 71) Healthcare workers described their guilt in increasing the workload of already overworked colleagues when they needed to isolate.(72-74) This 'extra pressure on the remaining staff'(75) had organisational implications on staffing numbers and created an 'ethical dilemma' because of the impact on health service provision.(72, 76)

> *I'm a carer for vulnerable adults and would be worried about them receiving care that I could not give if I was isolating* (Survey).(53)

Impact on mental health

People also described negative effects of isolation on mental health,(59, 70, 77) including loneliness, boredom, lack of interaction with friends and family, the inability to exercise, strain on their interpersonal relationships and harmony within a household, and anxiety over finances.(27, 30, 33, 35, 36, 45) University students in particular described struggling with losing access to their usual coping mechanisms(36) and that isolation exacerbated existing mental health problems.(30) Parents were concerned that isolating took school students away from their peers, education, and outdoor activities, which risked their mental health.(45)

> *I think mental health was like my biggest challenge. It was very easy to just feel down and not wanting to do things, not feel motivated to either do work or just get out of bed (*Focus group 8, student 5).(36)

The negative impact self-isolating had on mental health was described by others as a main barrier to self-isolation.(26, 59) People anticipated that self-isolation would negatively impact their mental health,(36, 53) and the unwillingness to self-isolate was exacerbated by people becoming 'fed up' with missing out on social interactions and other activities as the pandemic progressed.(54)

Impact on finances

Existing financial hardship was described as an important barrier to isolating.(24, 27, 78) In addition, self-isolation created financial hardship that acted as a barrier to reporting positive test results, in order to avoid isolation and its financial consequences.(24, 68, 79-84) These challenges were in large part ascribed to loss of income through loss of work or wages.(26, 53, 54, 58, 77, 79, 85)

For peer review only - http://bmjopen.bmj.com/site/about/guidelines.xhtml

The consequences of self-isolation for certain groups in society, such as those who were homeless, misusing substances, or involved in the criminal justice system, may have differed from those experienced by the wider population, and circumstances may have made self-isolation more difficult to manage and influenced decisions to isolate for these groups.(33) In particular, the financial consequences and considerations were not uniform, with some reporting that they could absorb the loss of income or worked from home already, while others could not 'afford to isolate'.(19, 77, 81, 85) The financial implications of isolation were described as particularly challenging for those with ethnic minority backgrounds, younger people, women, those on lower or precarious incomes, those unable to work from home while self-isolating, those working in 'shut-down sectors', migrant workers, and those who were self-employed, impacting adherence to self-isolation recommendations in these groups.(20, 35, 80, 85, 86)

Discussion

This study describes six categories of factors that influenced the decision to self-isolate in the UK during the COVID-19 pandemic, mapped onto the COM-B framework as: capability (perceived ability and information and guidance), opportunity (logistics and social influences, including trust in authority), and motivation (perceived value and consequences).

Decision to isolate

Ultimately, adherence to isolation guidance was a decision that was re-made over the period of required isolation and as individuals' lives changed. Individuals' adherence to guidance ranged from full adherence at a personal cost,(21, 87) to partial adherence where isolation was broken for reasons they believed to be 'essential' or 'acceptable',(23) to not isolating at all despite a positive test result(25, 32) (including not disclosing their symptoms or hiding their COVID-19 status(86)), to avoidance of testing in the first place.(86) This spectrum of engagement has been reported for other non-pharmaceutical measures across the COVID-19 and other pandemics, with people reporting a range of intentional and non-intentional non-adherence behaviours,(88) influenced at least in part by 'perceived necessity'.(2)

The decision to isolate was shaped by many factors.(23, 25, 36, 59) These results highlighted that people's decision to comply with isolation guidance was supported when the guidance was clear and plainly communicated, there were social norms to isolate and have trust in authority, there was support for planning and managing isolation, there was a perceived risk to themselves or others, and there was clear perceived value of isolation in protecting others from COVID-19 transmission. However, practical logistical challenges, distrust of tests and the implementing institutions, and the negative consequences of isolating on daily life, mental health, and finances all reduced adherence to isolation.

The factors influencing self-isolation adherence reflect the factors that influenced engagement with other COVID-19 public health measures. In the UK, clarity and consistency of guidance,(89, 90) trust in authority,(90) perception of risk,(89) social norms,(91) and 'collective importance'(92) were also found to influence adherence to other COVID-19 guidelines,(89) uptake of vaccination,(92, 93) and adherence to other non-pharmaceutical measures such as social distancing.(1, 88, 90, 91) Mistrust in authority was found to explain a large proportion of the variance in vaccination hesitancy in the UK(92) and was repeatedly described in the context of non-adherence to social distancing regulations.(1, 90) Adherence to different public health measures during COVID-19 was also linked; for example, those more hesitant to get vaccinated for COVID-19 were also less likely to adhere to social distancing guidelines.(92)

For peer review only - http://bmjopen.bmj.com/site/about/guidelines.xhtml

The desire to protect others was also a common theme facilitating engagement with many public health measures to tackle COVID-19(89, 91, 94) as well as previous pandemics.(2) Other behaviours required of the public during the COVID-19 response had some individual benefit: measures like vaccination or social distancing had personal benefit for the individual through reduction of the personal severity of disease or likelihood of contracting COVID-19, which increased engagement with vaccination programmes(95) or social distancing guidance.(94) Conversely, there was very little perceived direct individual benefit to self-isolation. Rather, the protection of others was commonly described as a major source of the value of isolation and as a 'fundamental driver of behaviour during the COVID-19 pandemic'.(47) Feelings of altruism resulted in moral obligations to engage in pro-social behaviours(96); such behaviours are particularly important to curb transmission when uncomfortable and undesirable activities like isolation are required.(90)

The costs of self-isolation, however, were sufficiently high and significant to disincentivise not only adherence to isolation guidance when testing positive but also act as a deterrent from testing for COVID-19 in the first place.(5) Unlike the safety concerns influencing uptake of COVID-19 vaccinations,(92) the concerns around the consequences of self-isolating were centred around the financial, practical, and mental health effects of isolating. The interruption of daily life due to isolation was not only inconvenient(23, 27, 33, 36, 45, 68, 97) but also meant that obligations were not fulfilled, resulting in a transfer of the burden to family, friends, and colleagues and creating feelings of guilt for the person isolating.(27, 53, 68, 70, 71) Isolation sometimes meant loss of work or even loss of a job; for many this was too high a price to pay to prevent transmission,(26, 53, 77, 79, 85, 87) echoing the impacts of the adverse socioeconomic effects of isolation that have made it a less acceptable non-pharmaceutical intervention across other pandemics.(2, 98)

Recommendations for future pandemic responses

Meaningful support has been described as a critical component of the strategy to encourage engagement with COVID-19 public health measures.(90) The major barrier to adherence to isolation guidance was the perception of personal consequences; therefore, external financial, practical, and mental health support became an important facilitator of self-isolation.(23, 26, 33, 34, 78, 81, 99) Whether this came from the government, official agencies, or informal networks, tangible support reduced the perception of isolation as a challenge.(24) However, support schemes did not consistently improve isolation adherence,(41, 42) and uptake of the support offered was suboptimal.(31, 38)

Not all individuals experienced the COVID-19 pandemic in the same way: for example, COVID-19 had a disproportionate effect on more vulnerable groups in the UK, with higher rates of infection, morbidity, and mortality in some ethnic minorities, older people, and those living in deprived areas.(100) Influential factors interact so that the motivation to self-isolate played out differently depending on people's capability and opportunity to isolate. While some described self-isolation easy to manage,(18) others found it unmanageable in various ways.(21, 87) Contextual factors such as ethnicity and socioeconomics played an important role in engagement with self-isolation, as well as with other programmes across different COVID-19 prevention measures,(88, 101) for example considerably reducing access to and uptake of vaccination(102, 103) and increasing the negative impact of social distancing measures.(100) Therefore, support must be tailored to context and to an individual's specific challenges.(26) Advertising a range of easily accessible financial, practical, and mental health support systems for different levels of requirement, based on individual circumstances as well as the intensity of isolation required, could help to streamline support services and make them more accessible.

For peer review only - http://bmjopen.bmj.com/site/about/guidelines.xhtml

Communication was described as a crucial tool to facilitate adherence to self-isolation recommendations.(40) Confusing or rapidly changing guidance was a repeated theme across behavioural research conducted during the COVID-19 pandemic in the UK and frequently reported as a barrier to engagement with public health programmes.(89) When guidance does not seem to make practical sense it shifts the onus of decision-making onto individuals, who must make choices within the constraints of their personal priorities and context. Thus, official guidance must be particularly clear and unambiguous.(2) This could improve uptake across public health measures during a pandemic,(89) such as social distancing (1, 100) or vaccination.(104)

Beyond clear, consistent guidance to support understanding of when, where, and how to self-isolate(21, 25, 45-47) and providing information on available support,(31, 37-39) it was repeatedly recommended that communication strategies should address people's motivation to comply(98) in order to activate personal norms that facilitate pro-social behaviours and engagement with preventative measures.(89, 91, 93) It was suggested that this could be achieved through messaging that reinforces trust in the government and programmes, demonstrates that adherence to guidance is the social norm, creates awareness of the consequences of non-adherence to guidance, and highlights the perceived benefits to the wider community to appeal to altruism.(25, 37, 47, 52, 98)

Many of the factors influencing the decision to self-isolate may be weighed differently in another pandemic: the value of protecting others may change if a disease were to primarily affect different population groups, such as children, or if the severity differed from that of COVID-19. At the same time, if the negative consequences of isolating were mitigated, or the duration of required isolation was different, this would also shift the balance of the decision. Context matters in people's decision-making about health, and the decision to follow guidance cannot be considered in isolation.(105) Therefore, regardless of the lessons learned during the COVID-19 pandemic and their applicability to future pandemic preparedness, research is needed to understand what affects how people weigh the different factors influencing the decision to isolate and what could be leveraged to encourage people to prioritise the value over the costs in future health emergencies. Additionally, as perceptions and decisions changed over time, measures should be in place to collect real-time data in future pandemics and make decisions reactively.

Strengths and limitations

Most evidence included in this review was concerned with factors influencing the decision to test(5) and thus evidence on isolation behaviour was often drawn from studies with a primary focus on testing during COVID-19. Additionally, this review was conducted in a short space of time with a large team, so multiple people were involved in screening and extracting data, which could have introduced selection bias. Steps to mitigate this included overlap of screening and data extraction for a proportion of the sources, quality checks and review of original documents during write up. However, it is strengthened by the broad search strategy that comprehensively scoped the available literature, as well as by the inclusion of a large body of unpublished and confidential internal data from UKHSA. This data from UKHSA was able to supplement the paucity of information available on the factors affecting isolation behaviour in the published literature, and would otherwise not be available for synthesis, general dissemination, and contribution to discussions on future strategies. Additionally, we consulted a diverse set of stakeholders to identify additional sources of evidence and to contextualise and interpret the results as accurately as possible.

For peer review only - http://bmjopen.bmj.com/site/about/guidelines.xhtml

Conclusions

This review demonstrates that the decision to isolate was a complex and dynamic process influenced by multiple factors. All dimensions of the COM-B behavioural model must be in place for people to comply with isolation guidance: in addition to leveraging the value in protecting others and mitigating the negative consequences to motivate people to self-isolate, future pandemic response policies and programmes will need to ensure that individuals have the capability and opportunity to put that motivation into practice. Communication of guidelines must be clear and consistent; context-appropriate financial, practical, and mental health support is required; and building trust with the public will be critical to ensure the success of any public health response to a pandemic.

For peer review only

Additional information

List of tables and figures

Table 2. Summary of the search parameters and limits as well as the inclusion and exclusion criteria,[2] categorised according to the 'population, context, concept' search framework(13)

Figure 1. The dimensions of the Capability, Opportunity, and Motivation Model of Behaviour (COM-B), adapted from McDonagh et al's application to a systematic review of chlamydia testing facilitators.(106)

Figure 2. Preferred Reporting Items for Systematic Reviews and Meta-analyses extension for scoping review (PRISMA-ScR) flow diagram outlining the search and inclusion of the literature.

Figure 3. Overview of the themes describing the influences on people's decision to comply with isolation guidance.

Acknowledgements

We would like to extend our gratitude to the UK Health Security Agency for their invaluable collaboration in this scoping review. Their willingness to provide access to confidential internal documents significantly enriched our research and understanding of isolation behaviours during the COVID-19 pandemic in England. We would like to thank by name the team who helped to organise, screen and extract the data: Ainura Moldokmatova, Firdaus Mohandas, Katie Douglass, Umar Mahmood and Zoe Echanah. We would like to acknowledge the contributions of the scientific, clinical and technical experts who have offered valuable input, advice and support throughout the evaluation and preparation of the overarching report, particularly the contributions of the Scientific Advisory Group. Our thanks also go to the numerous healthcare professionals, policy makers, and community leaders who worked tirelessly through COVID-19 pandemic in England to ensure that patients received quality care and that communities were safe.

Availability of data and materials: Data are included as supplementary material. Further data requests can be directed to the corresponding author.

Patient and Public Involvement statement: This study was performed on a short timeline, with a rapid turnaround time for the report. Additionally, no primary data was collected. Therefore, no public stakeholder engagement was considered. However, stakeholders from UKHSA were consulted to identify additional sources of published and unpublished evidence (included as 'stakeholder-identified' sources), sense-check the findings, and help frame the results.

Author contributions:

Conceptualisation and methodology: CMK, BAW, SM, MW, RN, and LJW.
Analysis, integration, and interpretation: CMK, SD, BAW, AFL, AP, MA, SM, MW, and the members of the EY-Oxford Health Analytics Consortium.
Writing – original draft: CMK and SD.
Writing – review, editing, and approval: RN, BAW, AFL, AP, MA, LJW, SM, MW, and the members of the EY-Oxford Health Analytics Consortium. All named authors read and approved the final manuscript.

Declarations

Ethics approval and consent to participate: Not applicable.

Consent for publication: All authors have consented to publication.

Competing interest: All authors had financial support from the UK Health Security Agency (UKHSA) for the submitted work; EY LLP London has previously received payment for consultancy work and advisory on the NHS Test & Trace response from the UK Department of Health and Social Care, now

For peer review only - http://bmjopen.bmj.com/site/about/guidelines.xhtml

known as the UK Health Security Agency. There are no other relationships or activities that could appear to have influenced the submitted work.

Funding: This study was funded by Secretary of State for Health and Social Care acting as part of the Crown through the UK Health Security Agency (UKHSA), reference number C80260/PRO5331.

References

1. Williams SN, Armitage CJ, Tampe T, Dienes K. Public perceptions and experiences of social distancing and social isolation during the COVID-19 pandemic: a UK-based focus group study. BMJ Open. 2020;10(7):e039334.
2. Teasdale E, Santer M, Geraghty AWA, Little P, Yardley L. Public perceptions of non-pharmaceutical interventions for reducing transmission of respiratory infection: systematic review and synthesis of qualitative studies. BMC Public Health. 2014;14(589):1-17.
3. UK Health Security Agency (confidential internal document). Effectiveness of non-pharmaceutical interventions to reduce transmission of COVID-19 in the UK: A rapid mapping review. 2023.
4. Institute for Government Analysis. Timeline of UK government coronavirus lockdowns and measures, March 2020 to December 2021. 2022.
5. EY–Oxford Health Analytics Consortium Evaluation of the national COVID-19 testing programme in England between October 2020 and March 2022.; 2023.
6. Department of Health and Social Care. Government launches NHS Test and Trace service 2020 [cited 2023 29 October 2023]. Available from: https://www.gov.uk/government/news/government-launches-nhs-test-and-trace-service.
7. Kucharski AJ, Klepac P, Conlan AJK, Kissler SM, Tang ML, Fry H, et al. Effectiveness of isolation, testing, contact tracing, and physical distancing on reducing transmission of SARS-CoV-2 in different settings: a mathematical modelling study. Lancet Infect Dis. 2020;20(10):1151-60.
8. Arksey H, O'Malley L. Scoping studies: Towards a methodological framework. International Journal of Social Research Methodology: Theory and Practice. 2005;8(1):19-32.
9. Levac D, Colquhoun H, O'Brien KK. Scoping studies: Advancing the methodology. Implementation Science. 2010;5(1):1-9.
10. Peters MD, Godfrey CM, Khalil H, McInerney P, Parker D, Soares CB. Guidance for conducting systematic scoping reviews. Int J Evid Based Healthc. 2015;13(3):141-6.
11. Naidoo R, Andersen-Waine B, Dahal P, Dickinson S, Lambert B, Mills MC, et al. A multistage mixed-methods evaluation of the UKHSA testing response during the COVID-19 pandemic in England. medRxiv. 2022:2022.10.27.22281604.
12. Hanneke CR, Asada Y, Lieberman L, Neubauer LC, Fagen M. The Scoping Review Method: Mapping the Literature in " Structural Change " Public Health Interventions. SAGE Research Methods Cases Part 2. 2017.
13. Peters MD. In no uncertain terms: the importance of a defined objective in scoping reviews. JBI Database System Rev Implement Rep. 2016;14(2):1-4.
14. Ouzzani M, Hammady H, Fedorowicz Z, Elmagarmid A. Rayyan-a web and mobile app for systematic reviews. Systematic Reviews. 2016;5(1):1-10.
15. Gwet KL. Computing inter-rater reliability and its variance in the presence of high agreement. British Journal of Mathematical and Statistical Psychology. 2008;61(1):29-48.
16. Michie S, Stralen MMv, West R. The behaviour change wheel: A new method for characterising and designing behaviour change interventions. Implementation Science. 2011;6(42):1-11.
17. UK Health Security Agency (Confidential internal document). Compliance with self-isolation for those within NHS Test and Trace: Summary of the Evidence 2021.

For peer review only - http://bmjopen.bmj.com/site/about/guidelines.xhtml

For peer review only

18. UK Health Security Agency (confidential internal document). Isolation_Survey_Report. 2020.
19. Denford S, Towler L, Ali B, Treneman-Evans G, Bloomer R, Peto TE, et al. Feasibility and acceptability of daily testing at school as an alternative to self-isolation following close contact with a confirmed case of COVID-19: a qualitative analysis. BMC Public Health. 2022;22(1):742.
20. Atchison C, Bowman LR, Vrinten C, Redd R, Pristerà P, Eaton J, et al. Early perceptions and behavioural responses during the COVID-19 pandemic: a cross-sectional survey of UK adults. BMJ Open. 2021;11(1):e043577.
21. UK Health Security Agency (confidential internal document). COVID-19 Test and Trace Cases Insights Study, Wave 3, 12–16 April 2021. 2021.
22. Smith LE, Amlot R, Lambert H, Oliver I, Robin C, Yardley L, et al. Factors associated with adherence to self-isolation and lockdown measures in the UK: a cross-sectional survey. Public Health. 2020;187:41-52.
23. Woodland L, Mowbray F, Smith LE, Webster RK, Amlôt R, Rubin GJ. What influences whether parents recognise COVID-19 symptoms, request a test and self-isolate: A qualitative study. PLoS One. 2022;17(2):e0263537.
24. Kyle RG, Isherwood KR, Bailey JW, Davies AR. Self-isolation confidence, adherence and challenges: Behavioural insights from contacts of cases of COVID-19 starting and completing self-isolation in Wales. Cardiff, Wales: Iechyd Cyhoeddus Cymru/Public Health Wales; 2021.
25. Eraso Y, Hills S. Self-Isolation and Quarantine during the UK's First Wave of COVID-19. A Mixed-Methods Study of Non-Adherence. Int J Environ Res Public Health. 2021;18(13).
26. Watson D, Baralle NL, Alagil J, Anil K, Ciccognani S, Dewar-Haggart R, et al. How do we engage people in testing for COVID-19? A rapid qualitative evaluation of a testing programme in schools, GP surgeries and a university. BMC Public Health. 2022;22(1):305.
27. Institute for Connected Communities. Newham Council welfare check-in call pilot - Learning from a rapid evaluation 2021–2022: University of East London; 2022. Available from: https://repository.uel.ac.uk/download/6d85e68559f8c6330ed94896e2165009f6a7bd6bce232452200857a16a7ed2b9/2819325/Welfare%20Check-In%20Call%20Evaluation%20Report.pdf.
28. UK Health Security Agency (confidential internal document). Calls (not texts) work best to encourage self-isolation - results from a field experiment in the Test & Trace programme. 2020.
29. Knight H, Carlisle S, O'Connor M, Briggs L, Fothergill L, Al-Oraibi A, et al. Impacts of the COVID-19 Pandemic and Self-Isolation on Students and Staff in Higher Education: A Qualitative Study. Int J Environ Res Public Health. 2021;18(20).
30. Godfrey K, Bagust L, Baird J, Barker M, Batchelor J, Bryant S. Evaluation of the expanded Southampton pilot study (Phase 2) for use of saliva-based lamp testing in asymptomatic populations: Final report, 16th November 2020. 2020.
31. UK Health Security Agency (confidential internal document). Effect of test and trace support payments scheme on ease of self-isolation and isolation compliance: Analysis of ONS isolation compliance survey data. 2021.
32. UK Health Security Agency (confidential internal document). Kingston Hospital Tracing Pilot-Interim Findings. nd.
33. Burns J, Mc Goldrick N, Sigerson D, Edwards M, Culshaw S, Clark C, et al. A Health Inequalities Impact Assessment of the surveillance of COVID-19 in asymptomatic patients attending dental settings in Scotland. Community Dent Health. 2022;39(4):254-9.
34. UK Health Security Agency (confidential internal document). Test seeking behaviours; attitudes, barriers and facilitators; Briefing Note, 22.03.21. 2021.
35. Isherwood KR, Kyle RG, Gray BJ, Davies AR. Challenges to self-isolation among contacts of cases of COVID-19: a national telephone survey in Wales. J Public Health (Oxf). 2022.
36. Al-Oraibi A, Fothergill L, Yildirim M, Knight H, Carlisle S, O'Connor M, et al. Exploring the Psychological Impacts of COVID-19 Social Restrictions on International University Students: A Qualitative Study. Int J Environ Res Public Health. 2022;19(13).

For peer review only - http://bmjopen.bmj.com/site/about/guidelines.xhtml

37. UK Health Security Agency (confidential internal document). Understanding experiences of the Test and Trace Support Payment: A Qualitative Follow-up Study. 2021.
38. UK Health Security Agency (confidential internal document). COVID-19 Test and Trace Cases Insights Study, Wave 4, 10–15 May 2021. 2021.
39. UK Health Security Agency (confidential internal document). COVID-19 Test and Trace Cases Insights Study, Wave 8, 1–6 November 2021. 2021.
40. UK Health Security Agency (confidential internal document). Kirklees Self-isolation Pilot: Evaluation Report 2021.
41. UK Health Security Agency (confidential internal document). Lambeth Self-isolation Pilot: Impact Evaluation 2021.
42. UK Health Security Agency (confidential internal document). Impact evaluation of a temporary raise in the wage eligibility threshold for the Test & Trace Support Payment in Bradford. 2021.
43. UK Health Security Agency (confidential internal document). Kirklees Self-isolation Pilot: Evaluation Report. 2021.
44. UK Health Security Agency (confidential internal document). Evaluation of the Enduring Transmission Pilot in Peterborough, Fenland and South Holland. 2022.
45. UK Health Security Agency (confidential internal document). School pilots experience research - User research insights and recommendations. 2020.
46. UK Health Security Agency (confidential internal document). COVID-19 Test and Trace Contacts Insights Study, Wave 8: 9–16 August 2021. 2021.
47. Smith LE, Potts HWW, Amlot R, Fear NT, Michie S, Rubin GJ. Intention to adhere to test, trace, and isolate during the COVID-19 pandemic (the COVID-19 Rapid Survey of Adherence to Interventions and Responses study). Br J Health Psychol. 2022;27(3):1100-18.
48. Smith LE, West R, Potts HWW, Amlot R, Fear NT, Rubin GJ, et al. Knowledge of Self-Isolation Rules in the UK for Those Who Have Symptoms of COVID-19: A Repeated Cross-Sectional Survey Study. Int J Environ Res Public Health. 2023;20(3).
49. UK Health Security Agency (confidential internal document). Secondary schools research - Wave 2, Birmingham. 2021.
50. UK Health Security Agency (confidential internal document). COVID-19 Test and Trace Contacts Insights Study, Wave 6: 1–5 June 2021. 2021.
51. UK Health Security Agency (confidential internal document). DTCC (Daily Testing of Covid contacts) - Voice of the Customer insight update.
52. UK Health Security Agency (confidential internal document). Compliance with self-isolation: publishing findings of ONS commissioned surveys. 2021.
53. van der Scheer JW, Ansari A, McLaughlin M, Cox C, Liddell K, Burt J, et al. Guiding organisational decision-making about COVID-19 asymptomatic testing in workplaces: mixed-method study to inform an ethical framework. BMC Public Health. 2022;22(1):1747.
54. Marshall GC, Skeva R, Jay C, Silva MEP, Fyles M, House T, et al. Public perceptions and interactions with UK COVID-19 Test, Trace and Isolate policies, and implications for pandemic infectious disease modelling. [version 1; peer review: awaiting peer review] F1000Research. 2022;11:1005.
55. Bevan I, Stage Baxter M, Stagg HR, Street A. Knowledge, Attitudes, and Behavior Related to COVID-19 Testing: A Rapid Scoping Review. Diagnostics (Basel). 2021;11(9).
56. UK Health Security Agency (confidential internal document). Public perceptions overview, Data from Public Perceptions Tracker . 2021.
57. Chantziara S, Brigden LCA, McCallum CH, Craddock IJ. Using Digital Tools for Contact Tracing to Improve COVID-19 Safety in Schools: Qualitative Study Exploring Views and Experiences Among School Staff. JMIR Form Res. 2022;6(11):e36412.
58. Kierkegaard P, Micocci M, McLister A, Tulloch JSP, Parvulescu P, Gordon AL, et al. Implementing lateral flow devices in long-term care facilities: experiences from the Liverpool COVID-

19 community testing pilot in care homes- a qualitative study. BMC Health Serv Res. 2021;21(1):1153.
59. Denford S, Martin AF, Love N, Ready D, Oliver I, Amlot R, et al. Engagement With Daily Testing Instead of Self-Isolating in Contacts of Confirmed Cases of SARS-CoV-2: A Qualitative Analysis. Front Public Health. 2021;9:714041.
60. Bow SMA, Goddard A, Cope G, Sharp N, Schick J, Woods C, et al. An evaluation of a pilot of daily testing of SARS-CoV-2 contacts in acute hospital and ambulance trusts in England. Public Health. 2022;209:46-51.
61. UK Health Security Agency (confidential internal document). Brand perceptions tracker - wave 1. 2020.
62. Denford S, Martin AF, Towler L, Mowbray F, Essery R, Bloomer R, et al. A qualitative process analysis of daily contact testing as an alternative to self-isolation following close contact with a confirmed carrier of SARS-CoV-2. BMC Public Health. 2022;22(1):1373.
63. UK Health Security Agency (confidential internal document). NHS Test and Trace W50 & W51. 2021.
64. UK Health Security Agency. Public perceptions overview, Data from Public Perceptions Tracker . 2021.
65. UK Health Security Agency (confidential internal document). Research exploring the barriers and drivers to following guidance.
66. UK Health Security Agency (confidential internal document). DTCC – Research Findings January 2022. 2022.
67. UK Health Security Agency (Confidential internal document). Public Sector Self-Collect Evaluation – (Draft) Final Report 2021.
68. UK Health Security Agency (confidential internal document). Weekly pilot evaluation update, 12 March 2021. 2021.
69. Street A, Baxter MS, Christison S, Bevan I, Bauld L. Student views and experiences of asymptomatic COVID-19 testing at the University of Edinburgh. DiaDev Investigating Diagnostics in Global Health, The University of Edinburgh; 2021.
70. Blake H, Knight H, Jia R, Corner J, Morling JR, Denning C, et al. Students' Views towards Sars-Cov-2 Mass Asymptomatic Testing, Social Distancing and Self-Isolation in a University Setting during the COVID-19 Pandemic: A Qualitative Study. Int J Environ Res Public Health. 2021;18(8).
71. Street A, Lee SJ, Bevan I. The hidden burden of medical testing: public views and experiences of COVID-19 testing as a social and ethical process. BMC Public Health. 2022;22(1):1837.
72. Martindale AM, Pilbeam C, Mableson H, Tonkin-Crine S, Atkinson P, Borek A, et al. Perspectives on COVID-19 testing policies and practices: a qualitative study with scientific advisors and NHS health care workers in England. BMC Public Health. 2021;21(1):1216.
73. Nyashanu M, Pfende F, Ekpenyong M. Exploring the challenges faced by frontline workers in health and social care amid the COVID-19 pandemic: experiences of frontline workers in the English Midlands region, UK. J Interprof Care. 2020;34(5):655-61.
74. Nyashanu M, Pfende F, Ekpenyong MS. Triggers of mental health problems among frontline healthcare workers during the COVID-19 pandemic in private care homes and domiciliary care agencies: Lived experiences of care workers in the Midlands region, UK. Health Soc Care Community. 2022;30(2):e370-e6.
75. Vindrola-Padros C, Andrews L, Dowrick A, Djellouli N, Fillmore H, Bautista Gonzalez E, et al. Perceptions and experiences of healthcare workers during the COVID-19 pandemic in the UK. BMJ Open. 2020;10(11):e040503.
76. UK Health Security Agency (confidential internal document). Supporting COVID-19 testing and vaccination in a deprived local authority - A case study of Blackpool 2021.
77. UK Health Security Agency (confidential internal document). COVID-19 Test and Trace Contacts Insights Study, Wave 3: 1–10 April 2021. 2021.

For peer review only - http://bmjopen.bmj.com/site/about/guidelines.xhtml

78. UK Health Security Agency (confidential internal document). London Borough of Havering Isolation Outreach Pilot Evaluation nd.
79. Jayes L, Bogdanovica I, Johnston E, Chattopadhyay K, Morling JR, Devine S, et al. Perspectives of attenders and non-attenders to SARS-CoV-2 asymptomatic community testing in England: a qualitative interview study. BMJ Open. 2022;12(9):e064542.
80. UK Health Security Agency (confidential internal document). Evaluation of COVID-19 guidance and support for the migrant worker community delivered by voluntary and community sector organisations (VCSOs). 2022.
81. Dennis A, Robin C, Carter H. The social media response to twice-weekly mass asymptomatic testing in England. BMC Public Health. 2022;22(1):182.
82. UK Health Security Agency (confidential internal document). Adult Social Care Vaccination & COVID-19 Testing Report. 2022.
83. Smith LE, Potts HWW, Amlôt R, Fear NT, Michie S, Rubin GJ. Adherence to the test, trace, and isolate system in the UK: results from 37 nationally representative surveys. BMJ. 2021;372:n608.
84. UK Health Security Agency (confidential internal document). Baselining the asymptomatic staff testing journey in adult social care (ASC).
85. UK Health Security Agency (confidential internal document). London Borough of Havering Isolation Outreach Pilot Evaluation.
86. Mahmood F, Acharya D, Kumar K, Paudyal V. Impact of COVID-19 pandemic on ethnic minority communities: a qualitative study on the perspectives of ethnic minority community leaders. BMJ Open. 2021;11(10):e050584.
87. UK Health Security Agency (confidential internal document). COVID-19 Test and Trace Cases Insights Study. nd.
88. Hills S, Eraso Y. Factors associated with non-adherence to social distancing rules during the COVID-19 pandemic: a logistic regression analysis. BMC Public Health. 2021;21(1):352.
89. Wright L, Paul E, Steptoe A, Fancourt D. Facilitators and barriers to compliance with COVID-19 guidelines: a structural topic modelling analysis of free-text data from 17,500 UK adults. BMC Public Health. 2022;22(1):34.
90. Burton A, McKinlay A, Dawes J, Roberts A, Fynn W, May T, et al. Understanding Barriers and Facilitators to Compliance with UK Social Distancing Guidelines During the COVID-19 Pandemic: A Qualitative Interview Study. Behaviour Change. 2022;40(1):30-50.
91. Wu JS, Font X, McCamley C. COVID-19 social distancing compliance mechanisms: UK evidence. Environ Res. 2022;205:112528.
92. Freeman D, Loe BS, Chadwick A, Vaccari C, Waite F, Rosebrock L, et al. COVID-19 vaccine hesitancy in the UK: the Oxford coronavirus explanations, attitudes, and narratives survey (Oceans) II. Psychol Med. 2022;52(14):3127-41.
93. Radic A, Koo B, Gil-Cordero E, Cabrera-Sanchez JP, Han H. Intention to Take COVID-19 Vaccine as a Precondition for International Travel: Application of Extended Norm-Activation Model. Int J Environ Res Public Health. 2021;18(6).
94. Coroiu A, Moran C, Campbell T, Geller AC. Barriers and facilitators of adherence to social distancing recommendations during COVID-19 among a large international sample of adults. PLoS One. 2020;15(10):e0239795.
95. Ashworth M, Thunstrom L, Cherry TL, Newbold SC, Finnoff DC. Emphasize personal health benefits to boost COVID-19 vaccination rates. Proc Natl Acad Sci U S A. 2021;118(32).
96. Schwartz SH. Normative Influences on Altruism. Advances in Experimental Social Psychology Volume 10. Advances in Experimental Social Psychology1977. p. 221-79.
97. UK Health Security Agency (confidential internal document). COVID-19 Test and Trace Contacts Behavioural Insights Study.
98. Brooks SK, Webster RK, Smith LE, Woodland L, Wessely S, Greenberg N, et al. The psychological impact of quarantine and how to reduce it: rapid review of the evidence. Lancet. 2020;395(10227):912-20.

For peer review only - http://bmjopen.bmj.com/site/about/guidelines.xhtml

99. UK Health Security Agency (confidential internal document). Compliance with self-isolation for those within NHS Test and Trace: Summary of the Evidence. 2021.
100. Her Majesty's Government. Social Distancing Review: Report. London, united Kingdom: Her Majesty's Government,; 2021. p. 1-32.
101. Husted M, Gibbons A, Cheung WY, Keating S. COVID-19 vaccination hesitancy in adults in the United Kingdom: Barriers and facilitators to uptake. Health Psychol. 2023;42(8):584-92.
102. Mounier-Jack S, Paterson P, Bell S, Letley L, Kasstan B, Chantler T. Covid-19 vaccine roll-out in England: A qualitative evaluation. PLoS One. 2023;18(6):e0286529.
103. Abba-Aji M, Stuckler D, Galea S, McKee M. Ethnic/racial minorities' and migrants' access to COVID-19 vaccines: A systematic review of barriers and facilitators. J Migr Health. 2022;5:100086.
104. Leigh JP, Moss SJ, White TM, Picchio CA, Rabin KH, Ratzan SC, et al. Factors affecting COVID-19 vaccine hesitancy among healthcare providers in 23 countries. Vaccine. 2022;40(31):4081-9.
105. Matthias MS, Salyers MP, Frankel RM. Re-thinking shared decision-making: context matters. Patient Educ Couns. 2013;91(2):176-9.
106. McDonagh LK, Saunders JM, Cassell J, Curtis T, Bastaki H, Hartney T, et al. Application of the COM-B model to barriers and facilitators to chlamydia testing in general practice for young people and primary care practitioners: a systematic review. Implement Sci. 2018;13(1):130.

For peer review only

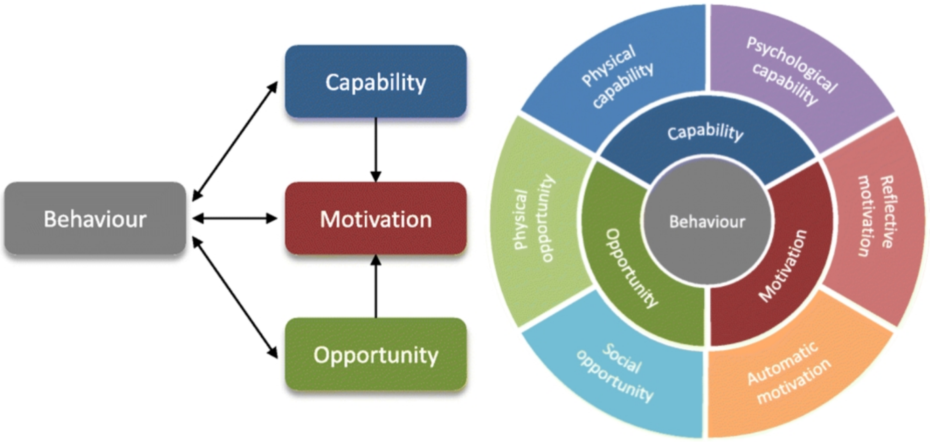

Figure 1. The dimensions of the Capability, Opportunity, and Motivation Model of Behaviour (COM-B), adapted from McDonagh et al's application to a systematic review of chlamydia testing facilitators.17

326x154mm (130 x 130 DPI)

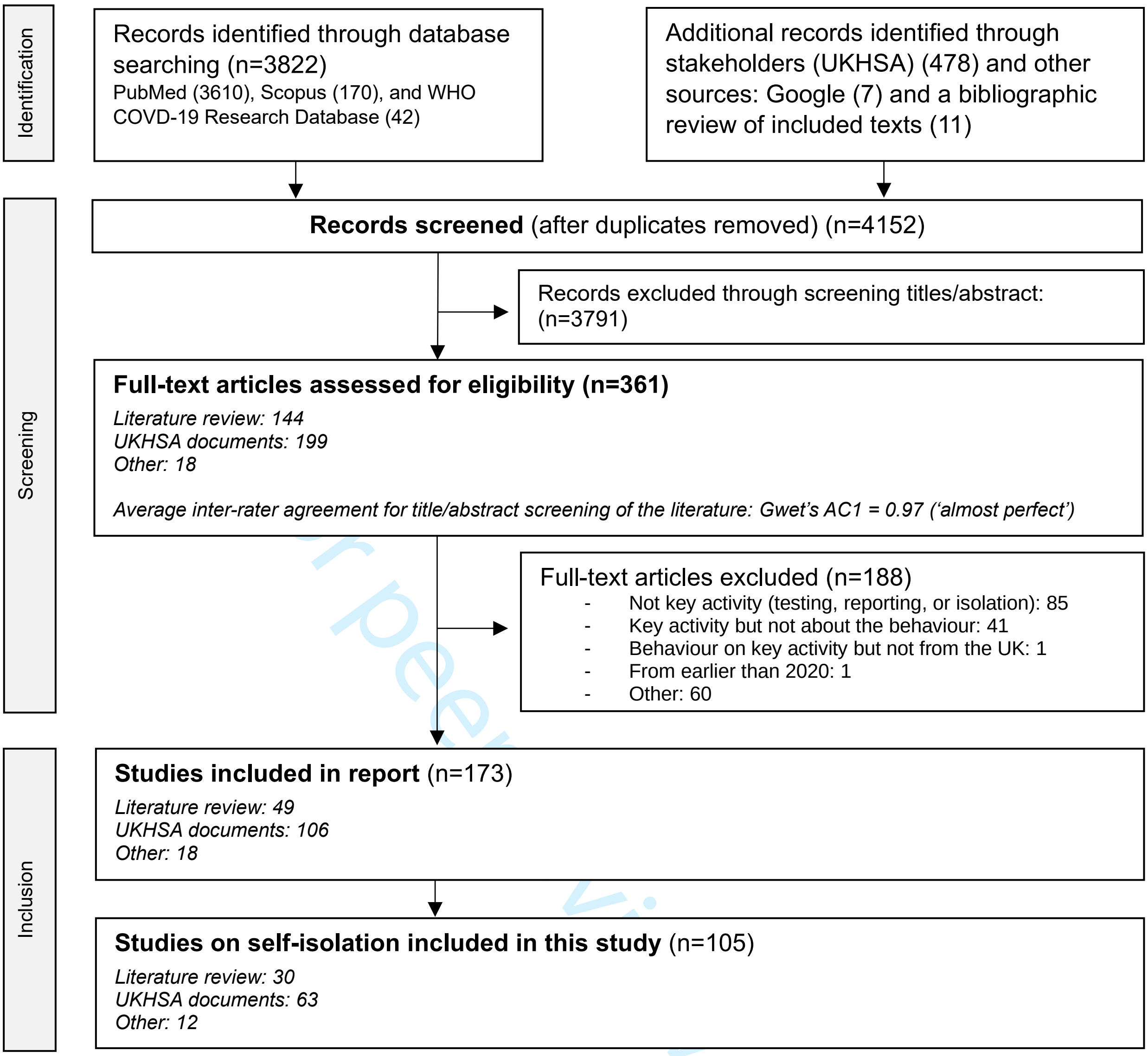

Figure 2. Preferred Reporting Items for Systematic Reviews and Meta-analyses extension for scoping review (PRISMA-ScR) flow diagram outlining the search and inclusion of the literature.

For peer review only

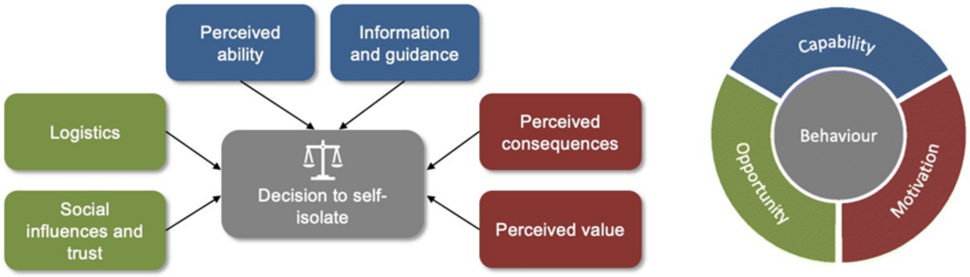

Figure 3. Overview of the themes describing the influences on people's decision to comply with isolation guidance.

402x137mm (57 x 57 DPI)

Decision to self-isolate during the COVID-19 pandemic in the UK: a rapid scoping study

Supplemental tables and figures

Supplemental Table 1. Search categories and examples of search terms. Search strings were adapted to each database. 2
Supplemental Table 2. Justifications and rationale for databases searched 3
Supplemental Table 3. Data extraction template 4
Supplemental Table 4. Included sources in the study examining self-isolation behaviour during the COVID-19 pandemic in the UK 5
Supplemental Table 5. Definitions of the stages of the pandemic 34

Supplemental Figure 1. Infographic to define the stages of the pandemic, with details 35

For peer review only

For peer review only - http://bmjopen.bmj.com/site/about/guidelines.xhtml

Supplemental Table 1. Search categories and examples of search terms. Search strings were adapted to each database.

Category	Search terms
**COVID-19**	COVID* OR corona OR coronavirus OR SARS-CoV-2 OR "SARS CoV 2" OR "SARS CoV-2" OR SARS-CoV2 OR SARSCoV2 OR "Severe Acute Respiratory Syndrome Coronavirus 2" OR "Severe Acute Respiratory Syndrome Corona Virus 2" OR 2019-nCoV
	**AND**
**Key activities**	test* OR screen* OR RT-PCR OR PCR OR “polymerase chain reaction” OR “lateral flow” OR “lateral flow device*” OR “lateral flow assay*” OR LFD OR self-test* OR “test and trace” OR “contact trac*” OR surveillance OR POCT OR report* OR self-report* OR selfreport* OR “test positive” OR “testing positive” OR result* OR “self-isolation” OR “self isolation” OR isolat* OR containment OR reopening OR re-opening OR mitigat* OR flatten*
	**AND**
**Behaviour, barriers, and facilitators**	knowledge OR understand* OR attitude* OR perception* OR perceive OR belief* OR believ* OR expectation* OR trust OR willing* OR intention* OR behaviour* OR behavior* OR practice* OR enact* OR engag* OR adher* OR complian* OR comply OR experience* OR view* OR motivation* OR barrier* OR block* OR challeng* OR difficult* OR facilitat* OR enabl* OR access* OR feasib* OR accept* OR uptake
	**AND**
**Research methods**	qualitative* OR interview* OR FGD OR "focus group*" OR survey* OR questionnair* OR mixed-method* OR “mixed method*” OR ethnograph* OR theme OR thematic* OR “grounded theory” OR “content analysis” OR field-work OR “field work” OR selfreport* OR self-report* OR “self report*” OR view* OR experience* OR hermeneutic OR phenomenolog*
	**AND**
**Geographic setting**	“United Kingdom” OR UK OR England OR Ireland OR Irish OR Scot* OR Wales OR Britain OR British OR NHS OR “National Health Service*” OR UKHSA OR “United Kingdom Health Security Agency” OR “UK Health Security Agency” OR “Channel Island*” OR London OR Birmingham OR Liverpool OR Manchester OR Cardiff OR Belfast OR Edinburgh OR Glasgow

For peer review only

Supplemental Table 2. Justifications and rationale for databases searched

Database	Rational for inclusion in the search strategy
**PubMed**	As the primary database for biomedical and health behaviour literature, PubMed not only covers Medline but also offers a vast range of other relevant sources. This includes in-process citations, out-of-scope citations, ahead of print citations, and author manuscripts of NIH funded research, making it a pivotal source for comprehensive and timely information.[1]
**Scopus**	Scopus provides interdisciplinary coverage, encompassing nearly 100 million biomedical, physical, life, and social sciences research records from over 7000 publishers. Its extensive citation analysis capabilities also help to understand the breadth and depth of literature influence. Additionally, the database is curated by independent subject matter experts, recognised as leaders in their fields, adding to the credibility for information for this study.[2]
**The World Health Organization COVID-19 Research Database**	The literature cited in the WHO COVID-19 Research Database was updated daily (Tuesday through Saturday) during the pandemic from searches of bibliographic databases, hand searching, and the addition of other expert-referred scientific articles. This database represents a comprehensive multilingual source of current literature on the topic, with the backing and expertise of a global health authority. While it may not be exhaustive, new research was added regularly. Databases searched include MEDLINE, Scopus, EuropePMC, Web of Science, ProQuest Central, EMBASE, medRxiv, ICTRP, WHO COVID, ScienceDirect, and grey literature.[3]
**Google Scholar**	Google Scholar covers a broad range of literature types, including theses, conference proceedings, and articles from academic publishers. Its inclusion can identify research overlooked in more specialized databases, ensuring comprehensive coverage and filling obvious gaps.
**Review of bibliographies of included studies**	A classic snowballing method, reviewing bibliographies can unveil seminal and influential works that may not be captured by traditional database searches. This method ensures inclusion of studies deemed foundational or highly relevant by scholars in the field and identifies evidence deemed relevant by those sources included in the initial screening process.
**Stakeholder consultation**	A strength of scoping studies is the explicit involvement of stakeholders in not only framing the research question, sense-checking the results, and informing the interpretation but also in identifying critical sources of evidence that may otherwise have been missed.[4]<br>In this instance, the UK Health Security Agency (UKHSA) was consulted as a major stakeholder to identify research commissioned by UKHSA.

Supplemental Table 3. Data extraction template

Manuscript information																								
Publication											Methodology						Setting				Key messages	Notes	Bibliographic review	Operational insights
No.	Rayyan ID	Authors	Title	**Inclusion**	**Reason for exclusion**	**EXTRACTOR** ***[Name]***	Publication date *[year]*	Journal or Source	**Publication stage**	Notes	**Methods**	Participants *[Who were the interviews/ surveys with? Whose views and experiences are represented?]*	Number of participants	**Data collection period** ***NB!***	Obvious limitations *[copy and paste limitations from the limitations section]*	Notes *[e.g. if 'other' chosen, expand here]*	**Country**	**Scope**	**Site/ Setting**	Notes	***Summary of the findings*** *- bullet points - big picture overview of the key points for OUR question (not the aims of the paper) - add own summary of the key points (paraphrase and summarise) and can copy and paste the conclusions if they summarise the key findings we need*	*Anything that doesn't fit in the categories, explanations etc* *<As well as your thoughts and notes and interpretations>*		
1																								
2																								
3																								
4																								
5																								

	Isolation Behaviour - copy and paste results and quotes																							
	Isolation								Person														Other	
	Perceived attributes of the isolation itself						Trust		Knowledge				Individual and context											
	**Perceived PHYSICAL factors of isolation itself** *[e.g. physical burden of the isolation itself like (physical) isolation fatigue, discomforts of staying in isolation]*		**Perceived MENTAL factors of isolation itself** *[e.g. like anxiety around isolation, isolation fatigue, "dread" of isolation (again)]*		**Perceived RESOURCE factors of isolation itself** *[e.g. resource factors like financial costs to the user of the isolation process, time etc]*		**Trust in the process of isolation and system** *[e.g. credibility and trust in the institutions delivering/providing the isolation or guidance on isolation)*		**Understanding of isolation requirements** *[i.e. does the individual understand if and when they SHOULD isolate? e.g. when, which type of isolation, where to isolate and for how long, including for what symptom]*		**Perceived capability to isolate** *[i.e. does the individual understand HOW to isolate? e.g. understanding of how to do it (in theory) and feeling able to take the steps to isolate]*		**Logistics of isolation** *[i.e. ACCESS to isolation e.g. convenience, ease of access, ease of isolating, resources, ability to isolate within their home and separate from other family members (separate rooms, space etc)]*		**Personal factors influencing isolation** *[e.g. mental health, burnout, resilience, perceptions of risk of contracting COVID, perceptions of risk of consequences if contract COVID etc]*		**Perception of value in isolation** *[e.g. to oneself, to others, to society, as a duty etc, perceived responsibility towards others to isolate, perceptions of COVID threat level, perceptions of necessity to isolate]*		**Perceived consequences of isolation** *[e.g. e.g. not being to work, or having sick leave or having to look after others, impact on finances, education, work, career, caring responsibilities etc]*		**Social influences related to isolation** *[e.g. stigma in taking part/not taking part, perceived freedom to say no/yes to isolation)*		**Other** *[not captured by other categories]*	
	Barriers	Enablers	Barriers	Enablers	Barriers	Enablers	Barriers	Enablers	Barriers	Enablers	Barriers	Enablers	Barriers	Enablers	Barriers	Enablers	Barriers	Enablers	Barriers	Enablers	Barriers	Enablers	Barriers	Enablers
1																								
2																								
3																								
4																								
5																								

For peer review only - http://bmjopen.bmj.com/site/about/guidelines.xhtml

Supplemental Table 4. Included sources in the study examining self-isolation behaviour during the COVID-19 pandemic in the UK

No	Publication	Methodology			Setting			
	Reference	Methods	Description of the sample	Data collection period	Pandemic stage (early, mid, late)	Country	Scope	Context
**General**								
**Literature search**								
1	Al-Oraibi A, Fothergill L, Yildirim M, Knight H, Carlisle S, O'Connor M, Briggs L, Morling JR, Corner J, Ball JK, Denning C, Vedhara K, Blake H. Exploring the Psychological Impacts of COVID-19 Social Restrictions on International University Students: A Qualitative Study. Int J Environ Res Public Health. 2022;19(13).	Interview and focus group	A total of eight online focus groups were conducted, comprising 29 international students, with group sizes varying between three to five students. Seventeen one-to-one interviews were held with university staff members.<br>International students - focus groups<br>University staff - interviews	January - February 2021	Mid pandemic	International (including UK)	Isolation	Testing in universities - Focus groups held with international university students, and interviews held with university staff.
2	Blake H, Carlisle S, Fothergill L, Hassard J, Favier A, Corner J, Ball JK, Denning C. Mixed-methods process evaluation of a residence-based SARS-CoV-2 testing participation pilot on a UK university campus during the COVID-19 pandemic. BMC Public Health. 2022;22(1):1470.	Survey, interview, and focus group	University students living onsite<br>Student survey - 152<br>Staff interviews - 13<br>Student focus groups - 30	April - June 2021	Mid pandemic	England	Testing and isolation	Convergent parallel mixed methods evaluating intervention fidelity and barriers/enablers to implementation of an asymptomatic testing programme in university.
3	Blake H, Knight H, Jia R, Corner J, Morling JR, Denning C, Ball JK, Bolton K, Figueredo G, Morris DE, Tighe P, Villalon AM, Ayling K, Vedhara K. Students' Views towards Sars-Cov-2 Mass Asymptomatic Testing, Social Distancing and Self-Isolation in a University Setting during the COVID-19 Pandemic: A Qualitative Study. Int J Environ Res Public Health. 2021;18(8).	Focus groups	25 university students from 1 institution	October 2020	Mid pandemic	England	Testing and isolation	Focus group discussion with university students on testing and isolation during the pandemic. Thematic analysis conducted on output of focus groups.

For peer review only - http://bmjopen.bmj.com/site/about/guidelines.xhtml

4	Burns J, Mc Goldrick N, Sigerson D, Edwards M, Culshaw S, Clark C, Watling C, Braid R, O'Keefe E, Gorman M, Conway DI. A Health Inequalities Impact Assessment of the surveillance of COVID-19 in asymptomatic patients attending dental settings in Scotland. Community Dent Health. 2022;39(4):254-9.	Survey	35 dental team members from across Scotland participated in the consultation.	October 2020	Mid pandemic	Scotland	Testing and isolation	A Health Inequalities Impact Assessment (HIIA) was conducted to understand the differential impacts the programme would have on the population and to improve the accessibility of the programme
5	Denford S, Martin AF, Love N, Ready D, Oliver I, Amlot R, Yardley L, Rubin GJ. Engagement With Daily Testing Instead of Self-Isolating in Contacts of Confirmed Cases of SARS-CoV-2: A Qualitative Analysis. Front Public Health. 2021;9:714041. Epub 20210803. doi: 10.3389/fpubh.2021.714041.	Interviews	52 participants, with 35 who had taken part in a feasibility study to evaluate daily contact testing, and 17 who had declined the offer and opted to self-isolate	11 - 23 December 2020 and 4 - 12 January 2021	Mid pandemic	UK	Testing and isolation	Daily contact testing rather than asymptomatic testing – focus on attitudes towards contact testing as an alternative to contacts of positive cases having to isolate. Study ran before Universal Testing roll out.
6	Denford S, Martin AF, Towler L, Mowbray F, Essery R, Bloomer R, Ready D, Love N, Amlot R, Oliver I, Rubin GJ, Yardley L. A qualitative process analysis of daily contact testing as an alternative to self-isolation following close contact with a confirmed carrier of SARS-CoV-2. BMC Public Health. 2022;22(1):1373.	Interviews	60 - People who had been in close contact with a confirmed SARS-CoV-2 carrier and had consented to take part in the trial.	24 June - 8 July 2021	Mid pandemic	England	Testing & Isolation	Daily contact testing rather than asymptomatic testing – focus on attitudes towards contact testing as an alternative to contacts of positive cases having to isolate. Study covers before and after introduction of Universal Testing.
7	Dennis A, Robin C, Carter H. The social media response to twice-weekly mass asymptomatic testing in England. BMC Public Health.	Other – review of	5783 comments – members of public	5 April - 28 May 2021	Mid pandemic	England	Testing & Isolation	Analysis of social media comments from members of

For peer review only - http://bmjopen.bmj.com/site/about/guidelines.xhtml

	2022;22(1):182. Epub 20220127. doi: 10.1186/s12889-022-12605-2.	social media						the public in England about twice-weekly asymptomatic testing. Study is from period when Universal Testing was first introduced.
8	Eraso Y, Hills S. Self-Isolation and Quarantine during the UK's First Wave of COVID-19. A Mixed-Methods Study of Non-Adherence. Int J Environ Res Public Health. 2021;18(13). Epub 20210630. doi: 10.3390/ijerph18137015.	Survey, interview, and focus group	residents in six North London boroughs<br>681 - Survey<br>16 – interviews	1 - 31 May 2020	Mid pandemic	England	Isolation	Study conducted before Universal Testing was available
9	Hanley S, Raybould G, Baxter E, Gray J, Sharkey D, Walker KF. Maternity services' responses to the COVID-19 pandemic: how Public Health England guidance was implemented in practice. Journal of Hospital Infection. 2022;129:214-8.	Surveys	44 maternity healthcare workers from 33 NHS hospitals in England, including 14 midwives, 3 senior midwives, 2 senior trainee obstetricians, 21 consultant obstetricians, 1 clinical fellow, 1 service director, 1 labour ward coordinator and 1 clinical midwifery manager	November 2020 - July 2021	Mid pandemic	England	Testing and isolation	Patient participation in testing programme.
10	Isherwood KR, Kyle RG, Gray BJ, Davies AR. Challenges to self-isolation among contacts of cases of COVID-19: a national telephone survey in Wales. J Public Health (Oxf). 2022.	Surveys	2027 - Individuals were eligible for inclusion if they: (i) had been successfully contacted by TTP after forward contact tracing (ii) were a close contact of a confirmed case of COVID-19; (iii) were aged 18 years or over; (iv) resident in Wales: and (v) had completed their self-isolation period at the time of telephone survey. Contacts were excluded from the study if they were: (i) under the age of 18; (ii) currently self-isolating; (iii) not a resident in Wales	11 November - 1 December 2020	Mid pandemic	Wales	Isolation	Daily contact testing rather than asymptomatic testing – focus on attitudes towards contact testing as an alternative to contacts of positive cases having to isolate. Before Universal Testing
11	Jayes L, Bogdanovica I, Johnston E, Chattopadhyay K, Morling JR, Devine S, Richmond N, Langley T. Perspectives of attenders and non-attenders to SARS-CoV-2 asymptomatic community testing in England: a qualitative interview study. BMJ Open. 2022;12(9):e064542.	Interviews	With 18 members of the public who attended a community testing centre and 15 who had not	February 2021 - May 2021	Mid pandemic	England	Isolation	Community asymptomatic testing. Study period covers before and after the start of introduction of Universal Testing.

For peer review only - http://bmjopen.bmj.com/site/about/guidelines.xhtml

12	Knight H, Carlisle S, O'Connor M, Briggs L, Fothergill L, Al-Oraibi A, Yildirim M, Morling JR, Corner J, Ball J, Denning C, Vedhara K, Blake H. Impacts of the COVID-19 Pandemic and Self-Isolation on Students and Staff in Higher Education: A Qualitative Study. Int J Environ Res Public Health. 2021;18(20). Epub 20211012. doi: 10.3390/ijerph182010675.	Interview and focus groups	26 university staff interviews and 11 university students participating in focus groups	January 2021 - March 2021	Mid pandemic	England	Testing and isolation	University testing programme. Before the introduction of Universal Testing.
13	Mahmood F, Acharya D, Kumar K, Paudyal V. Impact of COVID-19 pandemic on ethnic minority communities: a qualitative study on the perspectives of ethnic minority community leaders. BMJ Open. 2021;11(10):e050584.	Interviews	19 - Community leaders recruited through organisations representing ethnic minority communities and religious places of worship	October - November 2020	Mid pandemic	England	Testing, reporting and isolation	Community testing focussing on ethnic minority communities. Before Universal Testing.
14	Martin AF, Denford S, Love N, Ready D, Oliver I, Amlot R, Rubin GJ, Yardley L. Engagement with daily testing instead of self-isolating in contacts of confirmed cases of SARS-CoV-2. BMC Public Health. 2021;21(1):1067.	Surveys	319 people who had agreed to daily testing 205 who were not offered daily testing - adult contacts of confirmed COVID-19 cases who consented to daily testing, and a comparison group of contacts who were not offered testing and instead self-isolated	11 – 23 December 2020 and 4 - 12 January 2021	Mid pandemic	England	Testing and isolation	Daily contact testing rather than asymptomatic testing – focus on attitudes towards contact testing as an alternative to contacts of positive cases having to isolate. Before Universal Testing.
15	Mowbray F, Woodland L, Smith LE, Amlot R, Rubin GJ. Is My Cough a Cold or Covid? A Qualitative Study of COVID-19 Symptom Recognition and Attitudes Toward Testing in the UK. Front Public Health. 2021;9:716421.	Interviews	40 people (21 members of the general population, 19 students)	30 November - 11 December 2020	Mid pandemic	England	Testing	Focus on symptom recognition to test with a mixture of general public and university students. Before Universal Testing.
16	Robin C, Symons C, Carter H. Local Community Response to Mass Asymptomatic COVID-19 Testing in Liverpool, England: Social Media Analysis. JMIR Form Res. 2022;6(8):e34422.	Other – review of media and social media comments	Overall, 1096 comments were sampled: 219 newspapers comments, 472 Facebook comments, and 405 tweets. - Members of Liverpool local community	2 November 2020 - 8 November 2020	Mid pandemic	England	Isolation	Social media analysis of responses and views towards community asymptomatic testing. Before Universal Testing.

For peer review only - http://bmjopen.bmj.com/site/about/guidelines.xhtml

17	Smith LE, Amlot R, Lambert H, Oliver I, Robin C, Yardley L, Rubin GJ. Factors associated with adherence to self-isolation and lockdown measures in the UK: a cross-sectional survey. Public Health. 2020;187:41-52.	Surveys	Adults in the UK recruited online - 217 with experience of isolation of 2240 total respondents	6 - 7 May 2020	Early pandemic	UK	Isolation	Focus on adherence to isolation rather than on testing. Relatively early in the pandemic – first lockdown. Before Universal Testing.
18	Smith LE, Potts HWW, Amlot R, Fear NT, Michie S, Rubin GJ. Intention to adhere to test, trace, and isolate during the COVID-19 pandemic (the COVID-19 Rapid Survey of Adherence to Interventions and Responses study). Br J Health Psychol. 2022;27(3):1100-18.	Surveys	12,976 - Selected only participants who lived in England due to differing restrictions across the four UK nations. and were eligible for the study if they were aged 16 years or over and lived in the United Kingdom. Quotas were applied based on age and gender (combined), and reflected targets based on data from the Office for National Statistics	27 April 2020 – 27 January 2021	Early- Mid pandemic	UK	Testing & Isolation	Before Universal Testing.
19	Smith LE, Potts HWW, Amlôt R, Fear NT, Michie S, Rubin GJ. Adherence to the test, trace, and isolate system in the UK: results from 37 nationally representative surveys. BMJ. 2021;372:n608.	Surveys	74 699 responses from 45 957 people living in the UK, aged 16 years or older	2 March 2020 to 27 January 2021	Early- Mid pandemic	UK	Testing, Reporting and Isolation	Levels of adherence to test, trace, and isolate are low, although some improvement has occurred over time. Practical support and financial reimbursement are likely to improve adherence. Targeting messaging and policies to men, younger age groups, and key workers might also be necessary.
20	Street A, Lee SJ, Bevan I. The hidden burden of medical testing: public views and experiences of COVID-19 testing as a social and ethical process. BMC Public Health. 2022;22(1):1837.	Interviews	70 - members of the general public (aged 19–85) living in the Lothian region of Scotland	7 July - 24 September 2020	Early- Mid pandemic	Scotland	Isolation	Before Universal Testing.

For peer review only - http://bmjopen.bmj.com/site/about/guidelines.xhtml

21	van der Scheer JW, Ansari A, McLaughlin M, Cox C, Liddell K, Burt J, George J, Kenny R, Cousens R, Leach B, McGowan J, Morley K, Willars J, Dixon-Woods M. Guiding organisational decision-making about COVID-19 asymptomatic testing in workplaces: mixed-method study to inform an ethical framework. BMC Public Health. 2022;22(1):1747.	Surveys and interviews	50 survey participants and 11 interviews with staff members from workplaces used as case studies	November 2020 - December 2020	Mid pandemic	England	Testing and isolation	Exploring how workers feel about workplace testing and the implications of testing and isolation on their work. Before Universal Testing.
**Google/ Bibliographic search**								
1	Atchison C, Bowman LR, Vrinten C, Redd R, Pristerà P, Eaton J, Ward H. Early perceptions and behavioural responses during the COVID-19 pandemic: a cross-sectional survey of UK adults. BMJ Open. 2021;11(1):e043577.	Surveys	Nationally representative sample of 2108 UK adults	17 March 2020 - 18 March 2020	Early pandemic	UK	Isolation	Study conducted within 48 hours of UK government advising public to stop non-essential contact with others and all unnecessary travel
2	Bevan I, Stage Baxter M, Stagg HR, Street A. Knowledge, Attitudes, and Behavior Related to COVID-19 Testing: A Rapid Scoping Review. Diagnostics (Basel). 2021;11(9).	Survey, interview, and focus groups	UK population (amongst other international samples)	March 2020 - January 2021	Early- Mid pandemic	UK	Testing and isolation	Rapid scoping review found existing research at time of review was limited in depth and scope.
3	Denford S, Towler L, Ali B, Treneman-Evans G, Bloomer R, Peto TE, Young BC, Yardley L. Feasibility and acceptability of daily testing at school as an alternative to self-isolation following close contact with a confirmed case of COVID-19: a qualitative analysis. BMC Public Health. 2022;22(1):742.	Interviews	Staff, students, and parents of students (n=63)	Not stated	Mid pandemic?	England	Testing, reporting and isolation	At time of study, close contacts of the case required to self-isolate at home for 10 days.
4	French C, Denford S, Brooks-Pollock E, Wheling H, Hickman M. Low uptake of COVID-19 lateral flow testing among university students: a mixed methods evaluation. Public Health. 2022; 204:54–62.	Surveys, interviews, and focus groups	University students. A total of 436 students completed the online survey, and 20 in-depth interviews were conducted	November - December 2020	Mid pandemic	England	Isolation	Based at one university assessing LFT testing uptake and the differences between demographic variations
5	Kyle RG, Isherwood KR, Bailey JW, Davies AR. Self-isolation confidence, adherence and challenges: Behavioural insights from contacts of cases of COVID-19 starting and completing	Survey, interviews, and focus group	General population with 13,531 responses from survey and 1011 telephone surveys	November 2020 - January 2021	Mid pandemic	Wales	Isolation	Report published by NHS Public Health Wales exploring factors associated to

For peer review only - http://bmjopen.bmj.com/site/about/guidelines.xhtml

	self-isolation in Wales. Cardiff, Wales: Iechyd Cyhoeddus Cymru/Public Health Wales, 2021.							adherence to isolation with the aim to inform content of public health messaging and support.
6	Lin L, Song Y, Wang Q, Pu J, Sun FY, Zhang Y, Zhou X, Larson HJ, Hou Z. Public Attitudes and Factors of COVID-19 Testing Hesitancy in the United Kingdom and China: Comparative Infodemiology Study. JMIR Infodemiology 2021;1(1): e26895	Other- Social listening	General population - 3856 UK tweets and 9299 Chinese Sina Weibo posts	1 June - 15 July 2020	Early pandemic	International (including UK)	Testing, reporting and isolation	Comparative analysis between UK and China to assess public attitudes and uptake regarding COVID-19 testing.
7	Love N, Ready D, Turner C, Yardley L, Rubin J, Hopkins S, et al. The acceptability of testing contacts of confirmed COVID-19 cases using serial, self-administered lateral flow devices as an alternative to self-isolation. medRxiv. 2021.	Interviews	General population (n=882)	11 - 23 December 2020 And 4 - 12 January 2021	Mid pandemic	England	Testing, reporting & isolation	Investigating a 'test to enable' approach as part of England's tracing strategy dur to suboptimal adherence to self-isolation guidance.
8	Marshall GC, Skeva R, Jay C, Silva MEP, Fyles M, House T, Davis EL, Pi L, Medley GF, Quilty BJ, Dyson L, Yardley L, Fearon E. Public perceptions and interactions with UK COVID-19 Test, Trace and Isolate policies, and implications for pandemic infectious disease modelling. [version 1; peer review: awaiting peer review] F1000Research. 2022;11:1005.	Interviews	20 adults over the age of 18 living in the UK	Summer 2021	Mid pandemic	UK	Testing, reporting and isolation	Interviews took place during a policy change (19 July 2021), so the findings may be overrepresenting aspects of the interview context. Whilst efforts were made to recruit a diversity of views, participants were necessarily those who chose to take part in this study.
9	Smith LE, West R, Potts HWW, Amlot R, Fear NT, Rubin GJ, Michie S. Knowledge of Self-Isolation Rules in the UK for Those Who Have Symptoms of COVID-19: A Repeated Cross-Sectional Survey Study. Int J Environ Res Public Health. 2023;20(3).	Surveys	78,573 responses from 51,881 participants of the general population (UK adults)	November 2020 - February 2022	Mid- Late pandemic	UK	Isolation	At time of study adherence to self-isolation in people who develop key COVID-19

For peer review only - http://bmjopen.bmj.com/site/about/guidelines.xhtml

								symptoms is low in the UK.
10	Street A, Baxter MS, Christison S, Bevan I, Bauld L. Student views and experiences of asymptomatic COVID-19 testing at the University of Edinburgh. DiaDev Investigating Diagnostics in Global Health, The University of Edinburgh, 2021.	Interviews	24 University students who had completed a 10-minute survey	November - December 2020	Mid pandemic	Scotland	Testing and isolation	Main goal at the time of the mass testing programme was to minimise transmission of COVID19 when students travelled home for winter break.
**Stakeholder identified sources (UKHSA Secretariat)**								
1	ICF Consulting Services Limited. Evaluation of the Enduring Transmission Pilot in Peterborough, Fenland and South Holland. 2022.	Interviews	Untreated - 764,686<br>Treated - 228<br>Total - 764,914 - A total of 717 treated people were included in the pilot's MI (individuals supported by Rosmini from June to December 2021). Of these, 504 individuals could be linked to CTAS data based on their postcode, gender, ethnicity, and age (measured at assessment date). Among these 504 treated people, 276 could not be used for analysis for various reasons. Removing these 276 individuals from the sample of 504 treated people linked to the CTAS database resulted in a final sample of 228 treated people.<br><br>The sample of 764,686 untreated people was obtained by considering all individuals aged 18-67 who resided in LADs other than Fenland, Peterborough, and South Holland (after excluding all LADs where other Test & Trace pilots were rolled out), and with non-missing value on all the variables of interest (demographics and outcome).	23 June - 22 November 2021	Mid pandemic	England	Isolation	Evaluation was completed in two stages, a qualitative process evaluation and an impact evaluation stage.
2	Institute for Connected Communities. Newham Council welfare check-in call pilot - Learning from a rapid evaluation 2021–2022: University of East London; 2022. Available from: https://repository.uel.ac.uk/download/6d85e6855	Interviews	41 - The research participants were adult males and females, aged 20 to 75+, from Black, Asian, and ethnic minority groups and White British, from	November - December 2021	Mid -Late pandemic	England	Isolation	'Other' settings.

For peer review only

For peer review only - http://bmjopen.bmj.com/site/about/guidelines.xhtml

	9f8c6330ed94896e2165009f6a7bd6bce2324522 00857a16a7ed2b9/2819325/Welfare%20Check-In%20Call%20Evaluation%20Report.pdf.		across the Borough, and UK nationals and non-nationals. Of the 1,282 who were consented to take part in the study by the Council, 571 were eligible for screening calls. The 41 interviewed participants were clustered around seven postcode areas within the Borough and represented the general demographic characteristics and proportions of Newham. All the research participants who took part in the study had tested positive for COVID-19.					
3	UK Health Security Agency (confidential internal document), Brand perceptions tracker - wave 1. 2020.	Surveys	2,029 adult - Adults aged 18+ in England	9 -17 December 2020	Mid pandemic	England	Testing & isolation	Universal Testing.
4	UK Health Security Agency (confidential internal document).Calls (not texts) work best to encourage self-isolation – results from a field experiment in the Test & Trace programme. 2020.	Other	6,812 trial participants (2652 control, 1219 texts only, 1503 calls only, 1438 texts + calls) - close contacts of confirmed case	17 August - 10 September 2020	Early- Mid pandemic	England	Isolation	Randomised controlled field trial who'd recently been in close contact with a confirmed case. At the time of study, 14-day isolation period required.
5	UK Health Security Agency (confidential internal document). Compliance with self-isolation for those within NHS Test and Trace: Summary of the Evidence. 2021.	Surveys	Exact number hasn't been stated - Surveys were conducted internally and externally as well as analysis of data collected through NHS Test and Trace. Data collection was done from the following sources:<br>1. Internal surveys<br>2. Test and trace data on contacts<br>3. ONS compliance with self-isolation surveys<br>4. Test and Trace compliance	1. Internal Survey<br>a) August - September 2020<br>b) November 2020 - March 2021<br>2) ONS	Early – Mid pandemic	England	Isolation	The issues in obtaining an accurate estimate of compliance include considering the complexities around what is and is not

For peer review only - http://bmjopen.bmj.com/site/about/guidelines.xhtml

			check-in calls data<br>5. Test and Trace compliance check-in calls - landline data	compliance surveys – February - March 2021				permitted, including changes over time; the understanding or misunderstanding of the requirements among those who are isolating and the impact this can have on their self-reports. Due to these complexities, it is useful to compare sources of data that use different approaches and methods for obtaining estimates as this can strengthen confidence in their reliability.
6	UK Health Security Agency (confidential internal document). Compliance with self-isolation: publishing findings of ONS commissioned surveys.2021.	Other	n/a - n/a	Not stated	?	England	Isolation	Summary document of the published findings of ONS commissioned surveys to understanding of how well people isolate and what drives compliance.

For peer review only

7	UK Health Security Agency (Confidential internal document), COVID-19 Test and Trace Contacts Behavioural Insights Study - Contacts not required to self-isolate; Wave 1. 2021.	Interviews	1100 adults- aged 18 years or over Respondents were sampled through the Contact Tracing and Advice (CTAS) database, held by NHS TT.	25 – 30 October 2021	Mid pandemic	England	Testing & isolation	Survey for adults who had been in contact with someone who had tested positive for COVID-19 and were not required to self-isolate due to vaccination status.
8	UK Health Security Agency (Confidential internal document), COVID-19 Test and Trace Contacts Behavioural Insights Study - Contacts not required to self-isolate; Wave 2. 2021.	Interviews	918 adults - Respondents were sampled through the Contact Tracing and Advice (CTAS) database, held by NHS TT.	22 – 27 November 2021	Mid pandemic	England	Testing & isolation	Survey for adults who had been in contact with someone who had tested positive for COVID-19 and were not required to self-isolate due to vaccination status.
9	UK Health Security Agency (Confidential internal document), COVID-19 Test and Trace Contacts Behavioural Insights Study - Contacts not required to self-isolate; Wave 3. 2022.	Interviews	1078 - Respondents were sampled through the Contact Tracing and Advice (CTAS) database, held by NHS TT.	10 - 15 January 2022	Late pandemic	England	Testing & isolation	Survey for adults who had been in contact with someone who had tested positive for COVID-19 and were not required to self-isolate due to vaccination status. Strongly advised to take daily LFD for 7 days or until 10 days since their last contact

For peer review only - http://bmjopen.bmj.com/site/about/guidelines.xhtml

								with a positive case if earlier.
10	UK Health Security Agency (Confidential internal document). COVID-19 Test and Trace Contacts Behavioural Insights Study - Contacts not required to self-isolate; Wave 5. 2022.	Interviews	1112 - Respondents were sampled through the Contact Tracing and Advice (CTAS) database, held by NHS TT.	21 - 26 February 2022	Late pandemic	England	Testing & isolation	Survey for adults who had been in contact with someone who had tested positive for COVID-19 and were not required to self-isolate due to vaccination status. Strongly advised to take daily LFD for 7 days or until 10 days since their last contact with a positive case if earlier.
11	UK Health Security Agency (confidential internal document). Daily Testing for Contacts of COVID-19 Cases (DTCC) – Evaluation. 2022.	Surveys	c.9,838 respondents - Customers'	17 December 2021 - 19 January 2022	Late pandemic	England	Testing, reporting & isolation	Interim findings from the evaluation of the Daily Testing of contacts of COVID-19 (DTCC) Question 2: To what extent are people following the guidance to test and acting appropriately on the results of the tests?
12	UK Health Security Agency (confidential internal document). Does mass testing reduce compliance with self-isolation of confirmed positive cases of SARS-CoV-2? Evidence from the Liverpool mass testing pilot. 2021.	Surveys	In total 247 participants with a Liverpool address had participated in the mass testing pilot and 277 tested positive through normal, symptomatic channels. In addition, the study included 3 participants who had participated in the mass testing pilot but did not have a Liverpool address and 678 participants who did not have a Liverpool address and tested positive through normal,	Not stated	Unclear	England	Isolation	There are concerns that participation in asymptomatic community testing may undermine adherence to self-isolation compliance among those who test positive for

For peer review only

			symptomatic channels. - All participants had tested positive for SARS-CoV-2, had their details passed to NHS Test and Trace and had supplied a telephone contact number.					SARS-CoV-2. Regression analysis and propensity score matching was used to assess the impact of participation in the Liverpool mass testing pilot on compliance.
13	UK Health Security Agency (confidential internal document). DTCC (Daily Testing of Covid Contacts) - Voice of the Customer Insight update. 2022.	Surveys	9838 - <customers at the main stages of the test, trace, and isolate journey>	17 December 2021 - 19 January 2022	Late pandemic	England	Testing & isolation	Universal Testing.
14	UK Health Security Agency (confidential internal document). DTCC – Research Findings January 2022. 2022.	Interviews	Unclear - Online focus groups and 18-24 y/o, none following COVID regulations or only following very sporadically	10 January - 17 January 2022	Late pandemic	England	Testing, reporting & isolation	Universal Testing.
15	UK Health Security Agency (confidential internal document). Effect of test and trace support payments scheme on ease of self isolation and isolation compliance- Analysis of ONS Isolation Compliance Survey Data. 2021.	Surveys	12,486 respondents - The analysis uses data from surveys of confirmed positive cases and contacts conducted by ONS and is broken down by characteristics including age, gender, ethnicity, and receipt of any benefits.	March - July 2021	Mid pandemic	England	Isolation	12th October 2020, UK government launched Test and Trace Support Payment (TTSP) scheme (£500 support payment for those needing to isolate and on low incomes).
16	UK Health Security Agency (Confidential internal document). Evaluation of COVID-19 guidance and support for the migrant worker community delivered by voluntary and community sector organisations (VCSOs). 2022.	Interviews	23 service users and 8 stakeholders - Service users (migrant workers) and stakeholders (staff from voluntary and community sector organisations)	29 March - 7 April 2022	Late pandemic	England	Testing & isolation	Previous work by the UKHSA Evaluation team found that Voluntary and community sector organisations (VCSOs) play an important role in delivering support to 'disproportionately impacted' and 'under-served' communities

For peer review only - http://bmjopen.bmj.com/site/about/guidelines.xhtml

								such as migrant workers.
17	UK Health Security Agency (confidential internal document). Exploring Compliance with Self-Isolation among Deprived Communities: A Qualitative Research Protocol. 2021.	Interviews	1104 - Deprived communities (postcodes in bottom three deciles Index of Multiple Deprivation)	15 - 19 March 2021	Mid pandemic	England	Isolation	NHS Test and Trace refreshed its business plan in December 2020, reemphasising the importance of breaking the transmission of COVID-19 and highlighting that role contact tracing and self-isolation, of those who have COVID and their close contacts, plays in achieving this aim.
18	UK Health Security Agency (confidential internal document). Impact evaluation of a temporary raise in the wage eligibility threshold for the Test & Trace Support Payment in Bradford. 2021.	Other	Individuals who started a self-isolation period and for whom a compliance outcome is observed (postintervention)= approx. 15,000; individuals who started a self-isolation period = (postintervention) approx. 83,000; individuals who were reached by Test & Trace (postintervention) = approx. 22,000. - Residents of Bradford/other LADs (cases or contacts) who started self-isolating in the pre- or post-intervention period; or those who were reached by Test & Trace via phone in the pre- or post-intervention period; or shared the names of one/more contacts in the pre- or post-intervention period. Note: Only individuals aged between 18 and 67 (working-age population) and who reside in Bradford or one among the other 303 English LADs are included in the analysis	10 April - 19 May 2021 And 1 March - 9 April 2021 (the 40 days preceding the pilot).	Mid pandemic	England	Testing, reporting & isolation	Between 10th April and 19th May 2021, the wage eligibility threshold for the TTSP was raised in Bradford from £350 to £500 (people within this income range, defined newly eligible individuals, were the main target of the intervention). It was hoped that the intervention would help increase the proportions of individuals claiming and getting TTSP.
19	UK Health Security Agency (Confidential internal document). Isolation Survey Report. 2020.	Surveys	2783 respondents (1818 cases, 965 contacts)	16 November	Early pandemic	England	Isolation	The survey displays

For peer review only - http://bmjopen.bmj.com/site/about/guidelines.xhtml

				to 7 December 2020				difference of attitudes and risk perceptions, which were measured in 5 points scale, between self-isolation compliers and non-compliers. The variation can be seen at items how easy to self-isolate, confidence in self-isolation to reduce virus spread, trust of information about Covid-19 from government, and whether government has enacted right measures to protect public. In risk perception, there was a differing perceived risk intensity of Covid-19 to society.
20	UK Health Security Agency (confidential internal document). Kingston Hospital Tracing Pilot-Interim Findings.	Other	Baseline period (March 22nd-June 17th, 2021): 759<br><br>Pilot period (from June 18th-4th November): 2364 - Hospital patients	Baseline period: 22 March -17 June 2021<br><br>Pilot period: 18 June - 4 November 2021	Mid pandemic	England	Reporting & Isolation	In February 2021, we identified that 12% of cases (163 people) that had come through to LCT in Kingston had not completed contact tracing because they were in hospital. To address this gap in contact tracing and offer

For peer review only - http://bmjopen.bmj.com/site/about/guidelines.xhtml

								local welfare support to potentially vulnerable resident cases in hospital, Kingston started the Hospital Tracing pilot in June 2021.
21	UK Health Security Agency (confidential internal document). Kirklees Self-isolation Pilot: Evaluation Report. 2021.	Mixed methods	Not clear - Inclusion criteria for the pilot were that individuals:<br><br>1) were required to self-isolate following a positive COVID-19 result; or as a close contact of a confirmed COVID-19 case;<br>2) adequately demonstrated a loss in income due to self-isolation;<br>3) earned £26,000 or less per annum;<br>4) were unable to work from home.	27 May – 22 June 2021	Mid pandemic	England	Testing & isolation	The Kirklees self-isolation pilot is aimed at improving self-isolation outcomes, among other key metrics, using a financial mechanism. The pilot trials a revised version of the Test and Trace Support Payment Scheme (TTSP) with a broader set of eligibility criteria and an income replacement policy. The pilot ran for four weeks between 27 May and 22 June 2021.
22	UK Health Security Agency (confidential internal document). Lambeth Self-isolation Pilot: Impact Evaluation.	Other	not clear - Residents of Lambeth earning up to £30,000 per year were eligible to apply for financial support during self-isolation	Not stated	Unclear	England	Testing & isolation	Universal Testing.
23	UK Health Security Agency (confidential internal document). London Borough of Havering Isolation Outreach Pilot Evaluation.	Mixed methods	not clear - The pilot only visited index cases in the community and didn't visit close contact, those in hospital, social care provision or deceased.	7 June - 18 July 2021	Mid pandemic	England	Isolation	To support residents who were isolating due to testing positive with COVID-19, Havering Council embarked on a

For peer review only - http://bmjopen.bmj.com/site/about/guidelines.xhtml

								pilot to visit all positive cases within the community twice across the residents 10-day isolation period
24	UK Health Security Agency (confidential internal document). Office for National Statistics - COVID Test and Trace Contacts Insights Study. Wave 5. 2021.	Interviews	918 - Respondents were sampled through the Contact Tracing and Advice (CTAS) database, held by NHS TT.	4 – 8 May 2021	Mid pandemic	England	Testing & isolation	Who had been in contact with someone who has tested positive for coronavirus (COVID-19) and were at (or near) the end of their 10-day self-isolation period.
25	UK Health Security Agency (confidential internal document). Office for National Statistics - COVID Test and Trace Contacts Insights Study. Wave 6. 2021.	Interviews	968 - Respondents were sampled through the Contact Tracing and Advice (CTAS) database, held by NHS TT.	1 – 5 June 2021	Mid pandemic	England	Testing & isolation	Who had been in contact with someone who has tested positive for coronavirus (COVID-19) and were at (or near) the end of their 10-day self-isolation period.
26	UK Health Security Agency (confidential internal document). Office for National Statistics - COVID Test and Trace Contacts Insights Study. Wave 7a. 2021.	Interviews	990 - Respondents were sampled through the Contact Tracing and Advice (CTAS) database, held by NHS TT.	28 June- 3 July 2021	Mid pandemic	England	Testing & isolation	Who had been in contact with someone who has tested positive for coronavirus (COVID-19) and were at (or near) the end of their 10-day self-isolation period.
27	UK Health Security Agency (confidential internal document). Office for National Statistics – COVID Test and Trace Contacts Insights Study. Wave 7b. 2021.	Surveys	1122 - adults aged 18 or more who has tested positive for Covid-19 and were at the end of their 10-day self-isolation period.	8 - 13 March 2022	Late pandemic	England	Isolation	
28	UK Health Security Agency (confidential internal document). Office for National Statistics - COVID	Interviews	946 - Respondents were sampled through the Contact Tracing and	9 – 16 August 2021	Mid pandemic	England	Testing & isolation	Who had been in contact with someone who

For peer review only - http://bmjopen.bmj.com/site/about/guidelines.xhtml

	Test and Trace Contacts Insights Study. Wave 8. 2021.		Advice (CTAS) database, held by NHS TT.					has tested positive for coronavirus (COVID-19) and were at (or near) the end of their 10-day self-isolation period.
29	UK Health Security Agency (confidential internal document). Office for National Statistics - COVID Test and Trace Cases Insights Study: Wave 7. 2021.	Surveys	881- adults aged 18 or more who has tested positive for Covid-19 and were at the end of their 10-day self-isolation period.	27 September - 2 October 2021	Mid pandemic	England	Isolation	
30	UK Health Security Agency (confidential internal document). Office for National Statistics - COVID Test and Trace Cases Insights Study. Wave 8. 2021.	Surveys	976 - adults aged 18 or more who has tested positive for Covid-19 and were at the end of their 10-day self-isolation period.	1 - 6 November 2021	Mid pandemic	England	Isolation	Adults who had tested positive for coronavirus (COVID-19) and who were at the end of their 10-day self-isolation period.
31	UK Health Security Agency (confidential internal document). Office for National Statistics - COVID Test and Trace Self-Isolation Insights Study. Contacts required to Self-isolate. Waves 9 & 10. 2021.	Interviews	n/a - Respondents were sampled through the Contact Tracing and Advice (CTAS) database, held by NHS TT.	October - November 2021	Mid pandemic	England	Testing & isolation	COVID Test and Trace Cases Insights Study aims to understand behaviour of individuals required to self-isolate after testing positive. Universal Testing- survey aimed to exclude those who had received both doses of the vaccine or did not need to isolate.
32	UK Health Security Agency (confidential internal document). Office for National Statistics - COVID Test and Trace Contacts Insights Study. Wave 9. 2021.	Surveys	895 - adults aged 18 or more who has tested positive for Covid-19 and were at the end of their 10-day self-isolation period.	29 November - 4 December 2021	Late pandemic	England	Isolation	Understand the behaviour of individuals required to self-isolate after testing positive for coronavirus (COVID-19), as well as the effects of isolation on

For peer review only - http://bmjopen.bmj.com/site/about/guidelines.xhtml

								mental health, work, and finances.
33	UK Health Security Agency (confidential internal document). Office for National Statistics - COVID Test and Trace Cases Insights Study. Wave 11. 2022.	Surveys	1006 - adults aged 18 or more who has tested positive for Covid-19 and were at the end of their 10-day self-isolation period.	7 - 12 February 2022	Late pandemic	England	Isolation	Adults who had tested positive for coronavirus (COVID-19) and who were at the end of their self-isolation period.
34	UK Health Security Agency (confidential internal document). Office for National Statistics - COVID Test and Trace Self-Isolation Insights Study. Contacts required to Self-isolate. Wave 11. 2022.	Interviews	1184 - Respondents were sampled through the Contact Tracing and Advice (CTAS) database, held by NHS TT.	1 December 2021	Late pandemic	England	Testing & isolation	Survey for adults who were required to self-isolate after being in contact with someone who tested positive for COVID-19 and were at (or near) the end of their 10-day self-isolation period. This survey aimed to exclude those who had received both doses of the vaccine or did not need to self-isolate for another reason.
35	UK Health Security Agency (confidential internal document).Office for National Statistics - Indicators of Clinically Extremely Vulnerable Individuals' Responses to COVID-19 Outbreak. COVID High Risk Group Insights. 2021.	Interviews	2979 - Clinically extremely vulnerable populations	18 - 30 January 2021	Mid pandemic	England	Testing & isolation	COVID High Risk Group Insights survey was compiled rapidly in response to policy questions on whether the population who had been advised to shield were following shielding guidance and other information.

For peer review only - http://bmjopen.bmj.com/site/about/guidelines.xhtml

36	UK Health Security Agency (confidential internal document). Office for National Statistics - International Arrivals Insights Study: Waves 4 & 5. 2021.	Interviews	1067 - The sample included adults who arrived in the UK on 8 and 11 October (Wave 4) and 5 and 8 (Wave 5).	October - November 2021	Mid pandemic	England	Testing & isolation	Adults who had arrived in the UK from countries or territories that were not on the red list and reported that they were quarantining at an address in England.
37	UK Health Security Agency (confidential internal document). Office for National Statistics - Test and Trace Cases Insights Study. Wave 1: Pilot. 2021.	Surveys	2552 - adults aged 18 or more who has tested positive for Covid-19 and were at the end of their 10-day self-isolation period.	1 - 13 February 2022	Late pandemic	England	Isolation	COVID Test and Trace Cases Insights Study aims to understand behaviour of individuals required to self-isolate after testing positive. Universal Testing.
38	UK Health Security Agency (confidential internal document). Office for National Statistics -Test and Trace Cases Insights Study. Wave 3. 2021.	Surveys	1168 - adults aged 18 or more who has tested positive for Covid-19 and were at the end of their 10-day self-isolation period.	12 - 16 April 2021	Mid pandemic	England	Isolation	
39	UK Health Security Agency (confidential internal document). Office for National Statistics- Test and Trace Cases Insights Study. Wave 4. 2021.	Surveys	1044 - adults aged 18 or more who has tested positive for Covid-19 and were at the end of their 10-day self-isolation period.	10 -15 May 2021	Mid pandemic	England	Isolation	
40	UK Health Security Agency (confidential internal document). Office for National Statistics- Test and Trace Cases Insights Study. Wave 5. 2021.	Surveys	1090- adults aged 18 or more who has tested positive for Covid-19 and were at the end of their 10-day self-isolation period.	7 -12 June 2021	Mid pandemic	England	Isolation	
41	UK Health Security Agency (confidential internal document). Office for National Statistics- Test and Trace Cases Insights Study. Wave 6. 2021.	Surveys	936- adults aged 18 or more who has tested positive for Covid-19 and were at the end of their 10-day self-isolation period.	5 -10 July 2021	Mid pandemic	England	Isolation	
42	UK Health Security Agency (confidential internal document). Serial testing of contacts in the context of institutions. 2020.	Interviews	9 - Full-time, part-time, contractors, freelancers, zero-hour contract who cannot work from home or a mix of working from home and outside.<br><br>Mixed experience with Covid-19 test, trace and self-isolation.	3 – 4 December 2020	Mid pandemic	England	Testing & isolation	Initial objectives included test to enable, improve case identification using LFDs for contacts and enable people to

For peer review only

								avoid unnecessary self-isolation.
43	UK Health Security Agency (confidential internal document). Public perceptions overview. Data from Public Perceptions Tracker 2021.	Surveys	around 1,000 each week - nationally representative sample of adults aged 18 or more	November 2021 report	Late pandemic	England	Testing & reporting	After a short increase, negative media recall of NHSTT appears to be falling. Trust in testing continues to fluctuate.
44	UK Health Security Agency (confidential internal document). Public Sector Self-Collect Evaluation – (Draft) Final Report.	Survey, interview, and focus group	not clear - Public sector employees	1st March 2021 to 30th June 2021	Mid pandemic	UK	Testing & reporting	This report evaluates the roll-out of Self-Test Collect (STC) in the public sector workplace and how it performed against the existing Assisted Test Sites (ATS) model for the public sector workplace
45	UK Health Security Agency (confidential internal document). Research exploring the barriers and drivers to following guidance. nd.	Focus Groups	6 focus groups, 6 interviews with 18–24-year-olds - X 2 groups of people most engaged with and following guidance<br>X 2 groups of people following guidance in a more Ad-hoc manner, picking and choosing<br>X2 groups of people least engaged with guidance and least likely to follow it<br><br>X6, 60-minute depth interviews with 18-24 y/Os	10th January and w/c 17th January 2022	Late pandemic	England	Testing & reporting	At time of research, long list of guidelines to consider, from when to wear a face covering, vaccinations, testing, isolation and what to do if you are a COVID contact.
46	UK Health Security Agency (confidential internal document). Serial contact testing pilots evaluation summary.	Survey, interview, and focus group	n/a – employees	January - Mach 2021	Mid pandemic	England	Testing & isolation	Evaluation summary document.
47	UK Health Security Agency (confidential internal document). Summary Evaluation Findings: Venue Alerts.	Mixed methods	n/a - Recipients of venue alerts in England	24 September 2020 - 19 July 2021	Mid pandemic	England	Isolation	Venues displayed an NHS QR code and kept a record of contacts details for customers,

For peer review only - http://bmjopen.bmj.com/site/about/guidelines.xhtml

								visitors and staff who did not sign in through the NHS Covid-19 app. These allowed people to register their attendance so that if a potential outbreak was later identified, others who were present could be notified of their potential risk with a 'venue alert'.
48	UK Health Security Agency (confidential internal document). Supporting COVID-19 testing and vaccination in a deprived local authority: A case study of Blackpool. 2021	Interviews	8 - Four members of Blackpool public health team, the programme director of a large VCFS organisation and 3 Community Champions	May and June 2021	Mid pandemic	England	Testing	Experiences of those working in Blackpool Council and its public health team in supporting testing and vaccination among vulnerable groups, as well as some of the local responses implemented to overcome challenges
49	UK Health Security Agency (confidential internal document). Test and Trace Contacts Insights Study: Wave 3. nd.	Surveys	1100 - adults aged 18 or more who has tested positive for Covid-19 and were at the end of their 10-day self-isolation period.	1 - 10 April 2021	Mid pandemic	England	Isolation	Who had been in contact with someone who has tested positive for coronavirus (COVID-19) and were at the end of their 10-day self-isolation period.
50	UK Health Security Agency (confidential internal document). Test and Trace Contacts Insights Study: Wave 4. nd.	Surveys	1194 - adults aged 18 or more who has tested positive for Covid-19 and	19 - 24 April 2021	Mid pandemic	England	Isolation	

For peer review only - http://bmjopen.bmj.com/site/about/guidelines.xhtml

			were at the end of their 10-day self-isolation period.					
51	UK Health Security Agency (Confidential internal document), Test seeking behaviours; attitudes, barriers and facilitators; Briefing Note, 22.03.21. 2021.	Other	NA: This is a literature revie	NA	NA	UK	Testing	Universal testing
52	UK Health Security Agency (confidential internal document). The effects of negative asymptomatic test result messages on understanding of residual risk and behavioural intentions. 2021.	Surveys	1200 - Online experiment (12-15th March 2021) with a representative sample of the UK population where participants imagined they had taken an LFT as part of an asymptomatic testing programme	12 - 15 March 2021	Mid pandemic	UK	Other	Some receiving a test-negative result from asymptomatic lateral flow testing may misunderstand this to mean no risk of being infectious, resulting in reduced adherence to guidelines Tested whether communicating residual risk information and behavioural implications in negative test result messages affects residual risk understanding and behavioural intentions.
53	UK Health Security Agency (confidential internal document). Understanding Compliance with Self- Isolation: Summary of Survey Findings 2021.	Surveys	42,892 contacts were invited and 6,813 responded. - Over 18 years contacts who were reached and advice by NHS Test and Trace to isolate.	25 August - 14 September 2020	Early – Mid pandemic	UK	Isolation	Universal Testing.
54	UK Health Security Agency (confidential internal document). Understanding experiences of the Test and Trace Support Payment: A Qualitative Follow-up Study. 2021.	Interviews	31 participants - ONS Cases and Contacts Insight Survey participants and were not asked to take part in further research as a result.	1 May 2021	Mid pandemic	England	Isolation	Follow-up qualitative study to the ONS Cases and Contact Insights Survey. Sampled from both confirmed cases

For peer review only - http://bmjopen.bmj.com/site/about/guidelines.xhtml

								and contacts of COVID-19.
55	UK Health Security Agency (confidential internal document), Understanding the impact of a COVID-19 outreach programme delivered by voluntary and community sector organisations (VCSOs) for migrant worker communities and the role of VCSOs in communicating public health messaging. 2022.	Interviews	23 service users and 8 staff members from 4 VCSOs - Voluntary and community sector organisation (VCSO) service users and stakeholders.	17 January - 31 March 2022	Late pandemic	England	Testing & isolation	Aimed to communicate coronavirus (COVID-19) guidance on testing, self-isolation, and vaccination to migrant worker communities to improve understanding and encourage uptake.
56	UK Health Security Agency (confidential internal document). Weekly TIEB updates. 12 March 2021. 2021.	Survey, interview, and focus group	n/a - Private, Public sectors employees, NHS employees	8 - 12 March 2021	Mid pandemic	England	Testing & isolation	Pilots' evaluation update document covering operational feasibility, behaviours, socioeconomic impact.
57	UK Health Security Agency (confidential internal document). YouGov- NHS Test and Trace W50 & W51. 2023.	Surveys	2222 adults - England adults (aged 18+)	6 - 19 December 2021	Late pandemic	England	Testing, reporting & isolation	This survey has been conducted using an online interview administered to members of the YouGov Plc UK panel of 800,000+ individuals who have agreed to take part in surveys.
**Schools setting**								
**Literature search**								
1	Chantziara S, Brigden LCA, McCallum CH, Craddock IJ. Using Digital Tools for Contact Tracing to Improve COVID-19 Safety in Schools: Qualitative Study Exploring Views and Experiences Among School Staff. JMIR Form Res. 2022;6(11):e36412.	Interviews	18 staff (4 senior management, 12 teachers, 1 teaching assistant, and 1 behaviour support manager)	4 June - 7 November 2020	Early- Mid pandemic	England	Other	Background to the study was whether schools required support to implement guidelines.

For peer review only

For peer review only - http://bmjopen.bmj.com/site/about/guidelines.xhtml

2	Woodland L, Mowbray F, Smith LE, Webster RK, Amlôt R, Rubin GJ. What influences whether parents recognise COVID-19 symptoms, request a test and self-isolate: A qualitative study. PLoS One. 2022;17(2):e0263537.	Interviews	18 parents of school-aged (4 to 18 years) children	30 November – 11 December 2020	Mid pandemic	England	Testing & isolation	Parents have additional responsibility of using test and trace systems on behalf of their children. Factors associated to use of England's NHS Test and Trace service.
**Evidence overlapping between general and schools service settings**								
1	Nyashanu M, Pfende F, Ekpenyong M. Exploring the challenges faced by frontline workers in health and social care amid the COVID-19 pandemic: experiences of frontline workers in the English Midlands region, UK. J Interprof Care. 2020;34(5):655-61.	Interviews	40 health and social care frontline workers, including 15 nurses, 10 managers and 15 support workers in the English Midlands	February 2020 to April 2020	Early pandemic	England	Testing	Data collected early in the pandemic before asymptomatic testing of healthcare workers was in place, and healthcare workers and patients were prioritised for symptomatic PCR testing
2	Watson D, Baralle NL, Alagil J, Anil K, Ciccognani S, Dewar-Haggart R, Fearn S, Groot J, Knowles K, Meagher C, McGrath C, Muir S, Musgrove J, Glyn-Owen K, Woods-Townsend K, Mortimore A, Roderick P, Baird J, Inskip H, Godfrey K, Barker M. How do we engage people in testing for COVID-19? A rapid qualitative evaluation of a testing programme in schools, GP surgeries and a university. BMC Public Health. 2022;22(1):305.	Interviews and focus groups	210 participants from 4 schools, 1 university and 2 general practices in the South East of England, participating in the Southampton COVID-19 Testing Pilot Programme: 8 general practice staff, 30 pupils, 21 school staff, 12 pupil/parent pairs, 13 parents, 12 senior school representatives, 81 university students, 28 university staff and 5 senior university representatives<br><br>In total: 77 interviews and 20 focus groups	4 June 2020 - 07 November 2020	Early Mid pandemic	England	Testing and isolation	Spoke to i) individuals who had been approached about taking part in the pilot Saliva Testing Programme including some who tested positive; ii) those who were approached but declined to take part; and iii) senior university, primary care and school representatives responsible for delivering the

For peer review only - http://bmjopen.bmj.com/site/about/guidelines.xhtml

								Saliva Testing Programme in their organisations.
**Google/ Bibliographic review- Schools**								
1	Godfrey K, Bagust L, Baird J, Barker M, Batchelor J, Bryant S. Evaluation of the expanded Southampton pilot study (Phase 2) for use of saliva-based lamp testing in asymptomatic populations: Final report, 16th November 2020. 2020.	Interviews and focus groups	Staff, students/pupils, and contractors at four schools (an infant, junior, primary, and secondary) n= 88 and at the University of Southampton were included n= 108	September 2020 - November 2020	Mid pandemic	England	Testing, reporting & Isolation	Purpose of the report is to describe the programme and provide a form of manual as guidance for groups undertaking similar testing.
**Stakeholder identified sources (UKHSA Secretariat)**								
1	UK Health Security Agency (confidential internal document). 2021. Secondary schools research - Wave 2, Birmingham 2021.	Focus groups	Mini groups of parents, teachers, and pupils from a range of schools in Birmingham which either encouraged testing or not	November 2021	Mid pandemic	England	Testing, reporting & isolation	Focus is on schools where testing is less encouraged, and parents/children are not testing regularly.
2	UK Health Security Agency (confidential internal document). 2020. School pilots experience research - User research insights and recommendations.	Survey, Interviews and focus groups	Conducted in-depth interviews with participating school staff, pupils, and parents across 9 schools. 15 in-depth interviews and 796 survey respondents	October - December 2020	Mid pandemic	England	Testing, reporting & isolation	Study conducted alongside school mass testing pilots.
**Healthcare**								
**Literature search**								
1	Martindale AM, Pilbeam C, Mableson H, Tonkin-Crine S, Atkinson P, Borek A, Lant S, Gobat N, Solomon T, Sheard S. Perspectives on COVID-19 testing policies and practices: a qualitative study with scientific advisors and NHS health care workers in England. BMC Public Health. 2021;21(1):1216.	Interviews	24 interviews with 13 participants: five senior scientific advisors (all male Caucasian) and eight healthcare workers (five male, four females, all Caucasian) including nurses, physicians, general practitioners, and allied healthcare workers	March - August 2020	Early pandemic	England	Testing, reporting and isolation	Focus on perceptions of healthcare workers and scientific advisors, with data collected early in the pandemic before asymptomatic testing of healthcare workers was in place, and

For peer review only - http://bmjopen.bmj.com/site/about/guidelines.xhtml

								healthcare workers and patients were prioritised for symptomatic PCR testing.
2	Vindrola-Padros C, Andrews L, Dowrick A, Djellouli N, Fillmore H, Bautista Gonzalez E, Javadi D, Lewis-Jackson S, Manby L, Mitchinson L, Mulcahy Symmons S, Martin S, Regenold N, Robinson H, Sumray K, Singleton G, Syversen A, Vanderslott S, Johnson G. Perceptions and experiences of healthcare workers during the COVID-19 pandemic in the UK. BMJ Open. 2020;10(11):e040503.	Mixed methods	30 interviews with healthcare staff members<br>101 newspaper articles and 146 000 social media posts to capture the direct or indirect perceptions and experiences of healthcare workers	December 2019 to the end of April 2020<br><br>Interviews took place in April 2020	Early pandemic	UK	Testing	Focus on healthcare worker perceptions and experiences during the first wave, with data collected before asymptomatic testing was available but healthcare workers and patients were prioritised for PCR testing
**Stakeholder identified sources (UKHSA Secretariat)**								
1	Bow SMA, Goddard A, Cope G, Sharp N, Schick J, Woods C, Jeffery K, Harrington D, Williams S, Rodger AJ, Finer S, Fowler T, Hopkins S, Tunkel SA. An evaluation of a pilot of daily testing of SARS-CoV-2 contacts in acute hospital and ambulance trusts in England. Public Health. 2022;209:46-51.	Surveys and interviews	60 healthcare workers completed the survey (58 who did daily contact testing and 2 who did not).<br>28 interviews included trust daily contact testing leads, a union representative and two participants per trust. Healthcare workers were from four large, multisite acute hospital trusts in London, Oxford and Lancashire and a London ambulance trust	9 January - 28 February 2021	Mid pandemic	England	Testing a nd isolation	Data collected within the evaluation period when asymptomatic testing of healthcare workers was in effect.<br><br>This paper focuses on daily contact testing rather than routine asymptomatic testing.
**ASC academic papers**								
**Literature search**								
1	Kierkegaard P, Micocci M, McLister A, Tulloch JSP, Parvulescu P, Gordon AL, Buckle P. Implementing lateral flow devices in long-term care facilities: experiences from the Liverpool	Interviews	15 staff from 9 care homes<br>~ Broad representation of staff working in a range of care homes (residential and nursing homes)	December 2020 –	Mid pandemic	England	Testing	Pilot that largely mirrors protocols

For peer review only - http://bmjopen.bmj.com/site/about/guidelines.xhtml

	COVID-19 community testing pilot in care homes- a qualitative study. BMC Health Serv Res. 2021;21(1):1153.		~ Staff who had received training on how to use the LFDs, were directly involved in working with the LFDs to administer visitor and staff testing, and had been working at the care homes prior to the first national lockdown in March 2020, with the rationale that such staff would have a longitudinal perspective from working before and during the pandemic, on how different testing regimes had influenced care home work, and vice versa.	January 2021				adopted in the UK at that time Sample focused on nursing homes Same time as initial vaccine rollout
2	Micocci M, Gordon AL, Allen AJ, Hicks T, Kierkegaard P, McLister A, Walne S, Hayward G, Buckle P. COVID-19 testing in English care homes and implications for staff and residents. Age Ageing. 2021;50(3):668-72.	Interviews	10 staff members from eight care homes in England —with more than 5 years' experience in the sector	July – August 2020	Early pandemic	England	Testing and reporting	The paper describes PCR testing and the feasibility of point of care testing to support the complexity of testing and detection of COVID-19.
3	Tulloch JSP, Micocci M, Buckle P, Lawrenson K, Kierkegaard P, McLister A, Gordon AL, García-Fiñana M, Peddie S, Ashton M, Buchan I, Parvulescu P. Enhanced lateral flow testing strategies in care homes are associated with poor adherence and were insufficient to prevent COVID-19 outbreaks: results from a mixed methods implementation study. Age and Ageing. 2021;50(6):1868-75.	Interviews	15 care home staff members (from 9 care homes)	December 2020 - January 2021	Mid pandemic	England	Testing and Isolation	Pilot that largely mirrored protocols adopted in the UK at that time. Sample focused on nursing homes Same time as initial vaccine rollout
**Google/ Bibliographic review**								
1	Nyashanu M, Pfende F, Ekpenyong MS. Triggers of mental health problems among frontline healthcare workers during the COVID-19 pandemic in private care homes and domiciliary care agencies: Lived experiences of care workers in the Midlands region, UK. Health Soc Care Community. 2022;30(2):e370-e6.	Interviews	40 semi-structured interviews with frontline workers from nursing homes and domiciliary care agencies	Not stated - but before May 2020	Early pandemic	England	Testing & Isolation	Early stages of pandemic, frontline health workers and patients adversely affected.

For peer review only - http://bmjopen.bmj.com/site/about/guidelines.xhtml

Stakeholder identified sources (UKHSA Secretariat)								
1	UK Health Security Agency (confidential internal document). 2022. Adult Social Care Vaccination & COVID-19 Testing Report.	Surveys	651 adult social care workers including those in care homes, domiciliary and supported living	27 April – 16 May 2022	Late pandemic	England	Testing, reporting, and isolating	Survey conducted following removal of vaccinations as requirement for deployment in adult social care. Further testing changes in April 2022 (outside of evaluation period).
2	UK Health Security Agency (confidential internal document). nd. Baselining the asymptomatic staff testing journey in adult social care (ASC)	Other	Spoke to stakeholders (# undefined) to create high-level and detailed end-to-end service maps<br>Reviewed existing research to identify user needs and mapped user pain points<br>Created a content inventory of adult social care testing guidance and plotted key documents onto the service map<br>Conducted a SWOT analysis (strengths, weaknesses, opportunities, and threats) to identify gaps and opportunities	Undefined	Unclear	England	Testing and reporting	Non-specific as timeline not known. Expected that due to detail, this is after introduction of twice weekly LFD + PCR testing
3	RWD002 Report: Root cause analysis of observed sensitivity of LFDs below that of pre-deployment expected baseline performance when used by Adult Social Care staff	Other	Dual tests data collected between December 2020 and December 2021<br>Reconciled and analysed to explore differences (if any) device sensitivity within the population user group<br>Reviewed Innova and Biotime LFD devices	24 December 2020 – 23 December 2021	Mid pandemic	England	Reporting and Isolation	Generally, when staff followed testing regimen, LFD sensitivity matched baseline expectation. Recognition that funding was available to support self-isolation, with acknowledgement that decisions made at LA level are not reviewed.

For peer review only - http://bmjopen.bmj.com/site/about/guidelines.xhtml

Supplemental Table 5. Definitions of the stages of the pandemic

Stage	Date range	Notable events
**1. Early stage pandemic**	February 2020 to August 2020	• First lockdown<br>• Introduction of self-isolation rules<br>• Access to PCR testing established and gradually increased<br>• Restrictions from first lockdown eased
**2. Mid-stage pandemic**	September 2020 to November 2021	• Increasing restrictions leading to two more national lockdowns<br>• NHS COVID app launches<br>• Introduction of COVID-19 vaccines<br>• Access to lateral flow device self-test kits for general population<br>• Four-stage roadmap to easing restrictions leading to most legal limits removed by end of July 2021
**3. End-stage pandemic**	December 2021 to March 2022	• Omicron variant spread leads to increase in vaccine boosters, and self-isolation rule changes<br>• February 2021 sees the removal of legal requirement to self-isolate (unvaccinated) and an end to contact tracing

For peer review only

Supplemental Figure 1. Infographic to define the stages of the pandemic, with details.

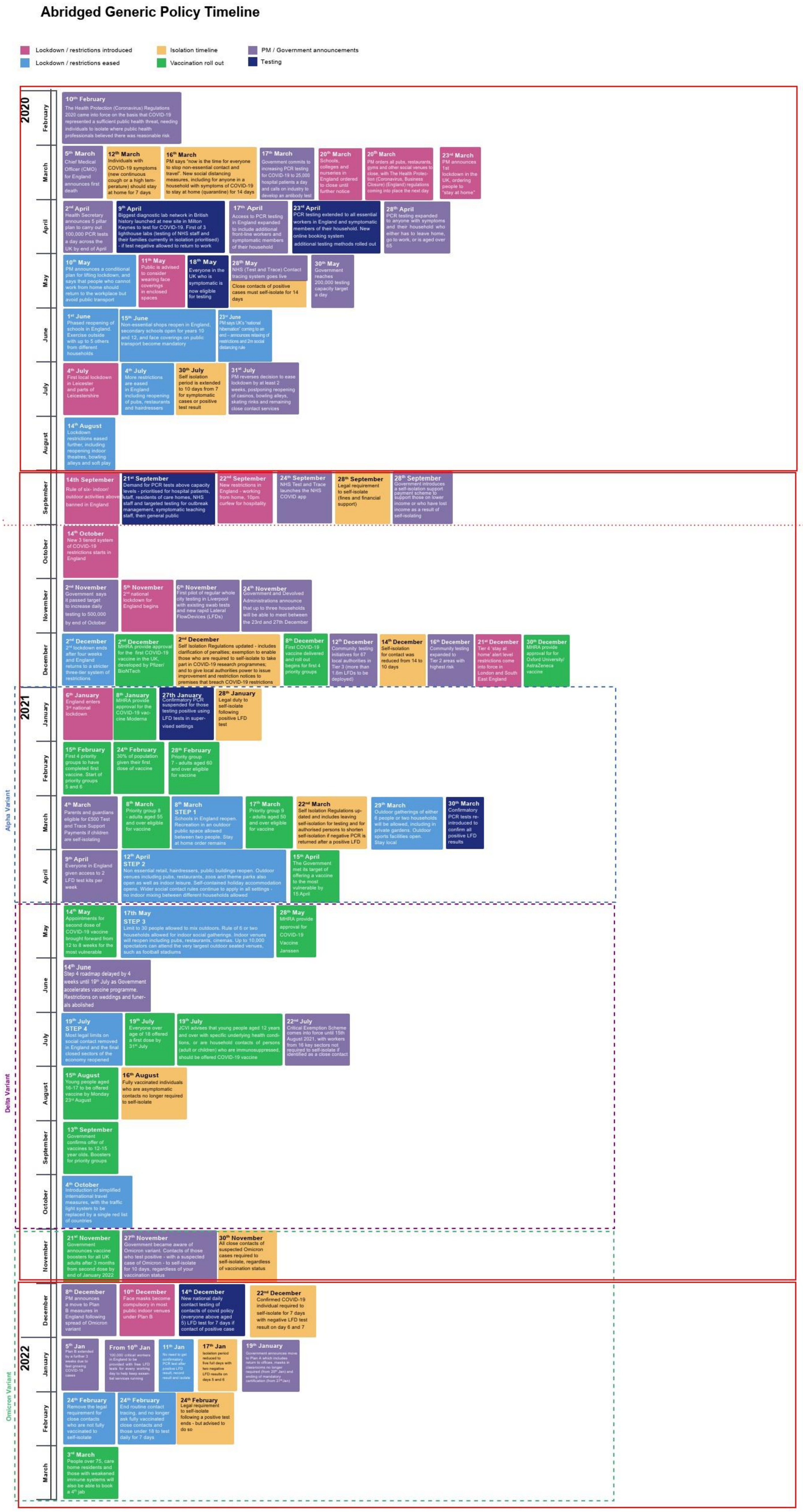

1. Early stage Pandemic

- First lockdown
- Introduction of self-isolation rules
- Access to PCR testing established and gradually increased
- Restrictions from first lockdown eased

2. Mid-stage Pandemic

- Increasing restrictions leading to two more national lockdowns
- NHS COVID app launches
- Introduction of COVID vaccines
- Access to LFD self-test kits for general population
- 4 stage roadmap to easing restrictions leading to most legal limits removed by end of July 2021

3. End stage of pandemic

- Omicron variant spread leads to increase in vaccine boosters and self isolation rule changes
- February 2021 sees the removal of legal requirement to self isolate (unvaccinated) and an end to contact tracing

Supplemental References

1. National Center for Biotechnology Information (NCBI). PubMed Overview: NCBI; 2023 [cited 2023 28 August]. Available from: https://pubmed.ncbi.nlm.nih.gov/about/.
2. Elsevier. Scopus. Your brilliance, connected: Elsevier; 2022 [cited 2023 28 August]. Available from: https://www.elsevier.com/en-gb/solutions/scopus.
3. Jose Garnica C. The WHO COVID-19 Research Database: behind the scenes of a super powerful tool! Library Science Talk2022.
4. Hanneke CR, Asada Y, Lieberman L, Neubauer LC, Fagen M. The Scoping Review Method: Mapping the Literature in " Structural Change " Public Health Interventions. SAGE Research Methods Cases Part 2. 2017. doi: https://dx.doi.org/10.4135/9781473999008.

For peer review only

For peer review only - http://bmjopen.bmj.com/site/about/guidelines.xhtml

Preferred Reporting Items for Systematic reviews and Meta-Analyses extension for Scoping Reviews (PRISMA-ScR) Checklist

SECTION	ITEM	PRISMA-ScR CHECKLIST ITEM	REPORTED ON PAGE #
**TITLE**			
Title	1	Identify the report as a scoping review.	1
**ABSTRACT**			
Structured summary	2	Provide a structured summary that includes (as applicable): background, objectives, eligibility criteria, sources of evidence, charting methods, results, and conclusions that relate to the review questions and objectives.	2
**INTRODUCTION**			
Rationale	3	Describe the rationale for the review in the context of what is already known. Explain why the review questions/objectives lend themselves to a scoping review approach.	3
Objectives	4	Provide an explicit statement of the questions and objectives being addressed with reference to their key elements (e.g., population or participants, concepts, and context) or other relevant key elements used to conceptualize the review questions and/or objectives.	3
**METHODS**			
Protocol and registration	5	Indicate whether a review protocol exists; state if and where it can be accessed (e.g., a Web address); and if available, provide registration information, including the registration number.	3
Eligibility criteria	6	Specify characteristics of the sources of evidence used as eligibility criteria (e.g., years considered, language, and publication status), and provide a rationale.	4 and supplementary material table 2
Information sources*	7	Describe all information sources in the search (e.g., databases with dates of coverage and contact with authors to identify additional sources), as well as the date the most recent search was executed.	4 and supplementary material
Search	8	Present the full electronic search strategy for at least 1 database, including any limits used, such that it could be repeated.	Supplementary material table 1
Selection of sources of evidence†	9	State the process for selecting sources of evidence (i.e., screening and eligibility) included in the scoping review.	4
Data charting process‡	10	Describe the methods of charting data from the included sources of evidence (e.g., calibrated forms or forms that have been tested by the team before their use, and whether data charting was done independently or in duplicate) and any processes for obtaining and confirming data from investigators.	4-5
Data items	11	List and define all variables for which data were sought and any assumptions and simplifications made.	4-5 and supplementary material table 3

For peer review only - http://bmjopen.bmj.com/site/about/guidelines.xhtml

SECTION	ITEM	PRISMA-ScR CHECKLIST ITEM	REPORTED ON PAGE #
Critical appraisal of individual sources of evidence§	12	If done, provide a rationale for conducting a critical appraisal of included sources of evidence; describe the methods used and how this information was used in any data synthesis (if appropriate).	NA
Synthesis of results	13	Describe the methods of handling and summarizing the data that were charted.	4-5
**RESULTS**			
Selection of sources of evidence	14	Give numbers of sources of evidence screened, assessed for eligibility, and included in the review, with reasons for exclusions at each stage, ideally using a flow diagram.	5 and figure 2
Characteristics of sources of evidence	15	For each source of evidence, present characteristics for which data were charted and provide the citations.	5 and supplementary material table 4
Critical appraisal within sources of evidence	16	If done, present data on critical appraisal of included sources of evidence (see item 12).	NA
Results of individual sources of evidence	17	For each included source of evidence, present the relevant data that were charted that relate to the review questions and objectives.	5-9
Synthesis of results	18	Summarize and/or present the charting results as they relate to the review questions and objectives.	5 and figure 3
**DISCUSSION**			
Summary of evidence	19	Summarize the main results (including an overview of concepts, themes, and types of evidence available), link to the review questions and objectives, and consider the relevance to key groups.	9-11
Limitations	20	Discuss the limitations of the scoping review process.	2 and 11
Conclusions	21	Provide a general interpretation of the results with respect to the review questions and objectives, as well as potential implications and/or next steps.	11
**FUNDING**			
Funding	22	Describe sources of funding for the included sources of evidence, as well as sources of funding for the scoping review. Describe the role of the funders of the scoping review.	12

JBI = Joanna Briggs Institute; PRISMA-ScR = Preferred Reporting Items for Systematic reviews and Meta-Analyses extension for Scoping Reviews.

* Where *sources of evidence* (see second footnote) are compiled from, such as bibliographic databases, social media platforms, and Web sites.

† A more inclusive/heterogeneous term used to account for the different types of evidence or data sources (e.g., quantitative and/or qualitative research, expert opinion, and policy documents) that may be eligible in a scoping review as opposed to only studies. This is not to be confused with *information sources* (see first footnote).

‡ The frameworks by Arksey and O'Malley (6) and Levac and colleagues (7) and the JBI guidance (4, 5) refer to the process of data extraction in a scoping review as data charting.

§ The process of systematically examining research evidence to assess its validity, results, and relevance before using it to inform a decision. This term is used for items 12 and 19 instead of "risk of bias" (which is more applicable to systematic reviews of interventions) to include and acknowledge the various sources of evidence that may be used in a scoping review (e.g., quantitative and/or qualitative research, expert opinion, and policy document).

For peer review only - http://bmjopen.bmj.com/site/about/guidelines.xhtml

From: Tricco AC, Lillie E, Zarin W, O'Brien KK, Colquhoun H, Levac D, et al. PRISMA Extension for Scoping Reviews (PRISMAScR): Checklist and Explanation. Ann Intern Med. 2018;169:467–473. doi: 10.7326/M18-0850.

For peer review only
